# An integrated linkage map of interspecific backcross 2 (BC_2_) populations reveals QTLs associated with fatty acid composition and vegetative parameters influencing compactness in oil palm

**DOI:** 10.1186/s12870-020-02563-5

**Published:** 2020-07-29

**Authors:** Zulkifli Yaakub, Katialisa Kamaruddin, Rajinder Singh, Suzana Mustafa, Marhalil Marjuni, Ngoot-Chin Ting, Mohd Din Amiruddin, Low Eng-Ti Leslie, Ooi Leslie Cheng-Li, Kandha Sritharan, Rajanaidu Nookiah, Johannes Jansen, Meilina Ong Abdullah

**Affiliations:** 1 Malaysian Palm Oil Board (MPOB), 6 Persiaran Institusi, Bandar Baru Bangi, 43000 Kajang, Selangor Malaysia; 2 United Plantations Bhd., Jendarata Estate, 36009 Teluk Intan, Perak Malaysia; 3 Wageningen University and Research Centre, P.O. Box 100, Wageningen, 6700 AC The Netherlands

**Keywords:** Oil palm, Interspecific hybrids, QTL, Fatty acid composition, Compactness

## Abstract

**Background:**

Molecular breeding has opened new avenues for crop improvement with the potential for faster progress. As oil palm is the major producer of vegetable oil in the world, its improvement, such as developing compact planting materials and altering its oils’ fatty acid composition for wider application, is important.

**Results:**

This study sought to identify the QTLs associated with fatty acid composition and vegetative traits for compactness in the crop. It integrated two interspecific backcross two (BC_2_) mapping populations to improve the genetic resolution and evaluate the consistency of the QTLs identified. A total 1963 markers (1814 SNPs and 149 SSRs) spanning a total map length of 1793 cM were integrated into a consensus map. For the first time, some QTLs associated with vegetative parameters and carotene content were identified in interspecific hybrids, apart from those associated with fatty acid composition. The analysis identified 8, 3 and 8 genomic loci significantly associated with fatty acids, carotene content and compactness, respectively.

**Conclusions:**

Major genomic region influencing the traits for compactness and fatty acid composition was identified in the same chromosomal region in the two populations using two methods for QTL detection. Several significant loci influencing compactness, carotene content and FAC were common to both populations, while others were specific to particular genetic backgrounds. It is hoped that the QTLs identified will be useful tools for marker-assisted selection and accelerate the identification of desirable genotypes for breeding.

## Background

Global palm oil production now stands at over 65 million tonnes/year, or 34% of the world vegetable oil production [1, 2]. The palm commonly planted commercially is the African oil palm (*Elaeis guineensis*). It is the most productive vegetable oil crop, with commercial oil yields of ~ 4 t/ha/yr [3], and up to 13 t/ha/yr in some breeding trials [4]. Although yield is the primary target, there is also need for disease resistance and tailored fatty acid composition (FAC) for the multivaried uses of the oil.

The oil palm, unfortunately, has only a single growing point, and continually grows taller making it more and more difficult to harvest, until well-nigh impossible. Having shorter (dwarf) palms will extend its economic life, while ensuing a lower (labour) cost of harvesting. The height increment of current commercial *Dura* x *Pisifera* (DxP) palms is 40–75 cm/yr [5], and planted palms can reach 15–18 m before replanting, but wild palms in the forest up to 30 m [6]. Breeding for dwarf palms started with Elmina Estate in Malaysia selfing the famous short Malayan Dumpy dura E206 [7]. More recently, MPOB identified Population 12 from its Nigerian prospection, not only for its dwarfness, but also for its high bunch number, good yield and desirable fruit characteristics [8]. These palms, when crossed with elite materials, are 5–10% shorter than the standard crosses [9]. In improving the palm, progress can be speeded up by biotechnology, using molecular markers to screen for the desired traits. Recently, quantitative trait loci (QTL) associated with trunk height and bunch weight were identified using a linkage map containing 1085 single nucleotide polymorphism (SNPs) [10]. A study [11] also constructed a consensus linkage map for a population of oil palm using simple sequence repeats (SSRs) and SNPs, and identified a major QTL for height on LG5. In another study using association mapping, a SNP marker, SNPG00006 *Fatl*, was observed to be significantly associated with height (*P* ≤ 0.05) [12].

All the above work was carried out on *E. guineensis*, the African oil palm. There is, however, a second oil palm, *Elaeis oleifera* – the American oil palm. Although not much commercially planted because of its very low yields, it has several interesting characteristics, such as shortness, less saturated oil and disease resistance, which may be introgressed to improve *E. guineensis*. Interspecific hybrids of *E. guineensis* and *E. oleifera* have already been made (F1) – they are shorter, but their yield still very low [13]. They also produce little pollen, and assisted pollination is required to produce even their low yield. Backcrossing to *E. guineensis* will quickly improve the yield, but just as quickly lose the desirable *E. oleifera* characteristics. In other words, the improvement of *E. guineensis* by introgressing *E. oleifera* traits has largely come to nought in a painstakingly slow and costly process.

But that was the past using conventional breeding. Now, with DNA-based markers there is promise of more efficient crop improvement by introgressing only the specific genes wanted, rather than half the whole genome of donor palms just for the few required genes. The availability of dense genetic maps for both *E. guineensis* and interspecific hybrids [14–17], as well as markers linked to important quantitative traits, such as yield, vegetative characters and FAC [10, 15, 18–20], provide the groundwork for this work. However, and interestingly, no QTL for height has yet been reported for the interspecific hybrid.

Compact palms with shorter trunks and fronds can be planted at a higher density than the current 148/ha. If the individual palm yields can be maintained, then the yield per unit area will increase [21, 22]. In South America, hybrid compact palms [23] have already been developed by multiple backcrossing of OxG hybrids to *E. guineensis* [24], and the outstanding palms cloned for planting [25]. In 2012, an OxG hybrid, known as COMPACT palms, was developed with low height increment (below 40 cm/year) and short fronds (~ 6.5 m), allowing high density planting (180–200 /ha) [26]. Backcrossing COMPACT palms to Deli, Ghana and Nigeria *E. guineensis* produced fronds of 6.6–6.9 m which reduced the plantable density to 170 palms/ha, but still higher than the current 148 palms/ha.

Interspecific hybrids and their backcrosses have desirable FACs in their oils. The genomic regions associated with various FAC traits in an OxG interspecific hybrid [14, 20] and interspecific backcross one (BC_1_) [16] mapping populations were identified via conventional QTL analysis. A number of these QTLs were validated across interspecific backcross two (BC_2_) mapping populations [20]. One of the major restrictions in associating markers to traits in oil palm is the size of the mapping populations used. Because of the sheer palm size, oil palm breeding trials generally consist of 64 palms per progeny, small for effective genetic mapping and QTL analysis. However, it is possible to develop high quality integrated maps of multi parental populations, which can enhance QTL discovery [15]. This study searched for the QTLs associated with vegetative traits and FAC in two BC_2_ mapping populations - characters important for developing compact palms with higher unsaturated mesocarp oil. It also sought to validate QTLs linked to FAC identified earlier. It integrated two BC_2_ populations to enhance the genetic resolution and assess the consistency of the QTLs detected, apart from identifying population-specific genomic regions influencing the traits.

## Results

### Traits of interest

The vegetative parameters, and mesocarp oil iodine value (IV), carotene and fatty acid contents in the 2.6–1 and 2.6–5 families are summarized in Table 1 and Additional file 1. All the traits showed wide segregation and more importantly, they showed continuous variation, suggesting quantitative inheritance. The means for rachis length and petiole cross-section area (referred to as petiole cross section in the text) were slightly higher in 2.6–1, but height increment and carotene content higher in 2.6–5. However, both rachis length and height increment were considerably lower in both families than in commercial DxP*,* where they are generally higher than 5 and 0.45 m, respectively [27]. For FAC, 2.6–1 had higher stearic (C18:0), oleic (C18:1), linoleic (C18:2) acid content and iodine value, whereas palmitic acid (C16:0) content was slightly higher (35.3%) in 2.6–5.

**Table 1:** Summary of vegetative parameters, iodine value, and carotene and fatty acid contents in 2.6–1 and 2.6–5 BC_2_ populations

Population		2.6–1					2.6–5				
Category	Variable	Mean	SD	CV	N	Range	Mean	SD	CV	N	Range
Vegetative	RL (m)	5.28	0.67	12.8	72	2.45–6.80	5.17	0.61	11.8	69	3.53–6.56
	HI (m)	0.34	0.07	19.8	72	0.21–0.60	0.38	0.06	16.2	69	0.27–0.53
	PCS (cm^2^)	26.6	5.58	20.9	72	10.8–40.8	26.4	5.20	19.6	69	15.6–40.5
FAC	Carotene (ppm)	1047	327	31.2	53	417–1966	1134	421	37.1	57	441–2661
	IV (%)	65.0	2.51	3.86	53	60.2–69.9	63.4	3.22	5.07	58	57.1–71.6
	C16:0 (%)	31.1	2.47	7.94	54	24.7–36.7	35.3	2.85	8.06	58	26.9–41.7
	C18:0 (%)	6.15	1.31	21.4	54	3.29–9.43	3.79	0.92	24.3	58	2.11–6.48
	C18:1 (%)	48.5	2.64	5.45	53	40.9–53.7	47.0	3.67	7.80	58	37.6–54.5
	C18:2 (%)	12.9	1.41	10.9	54	9.6–16.3	12.7	2.05	16.2	58	8.15–17.7

*RL* Rachis length, *HI* Height increment, *PCS* Petiole cross section, *Carotene* Carotene content, *IV* Iodine value, *C16:0* Palmitic acid content, *C18:0* Stearic acid content, *C18:1* Oleic acid content, *C18:2* Linoleic acid content

The relationships between the individual fatty acids were evaluated using Pearson’s correlation and consistent results obtained for both families (Tables 2 and 3). The most abundant saturated fatty acid, C16:0, was negatively correlated with the unsaturated fatty acids (C18:1 and C18:2). A negative correlation was also observed between C18:0 and C16:0 content. As expected, iodine value, as an indicator for the oil unsaturation, was positively correlated with C18:1 and C18:2 content, and negatively with C16:0 and C18:0 content. In addition, the correlations were positive between C18:0 and C18:2 content and negative between C18:1 and C18:2 content. Correlation trends for the vegetative parameters were similar in both populations (Tables 2 and 3). Petiole cross section was positively correlated with height increment and rachis length, while height increment and rachis length appeared negatively correlated, although not significant at *P* ≤ 0.05. Generally, the vegetative parameters related to compactness did not show significant correlation with the fatty acids. The exceptions were the positive correlations between height increment and petiole cross section with C18:2 content, and also between height increment and iodine value.

**Table 2:** Pearson’s correlations between individual fatty acid contents and iodine value with vegetative parameters in 2.6–1 population

Population	2.6–1							
	HI	PCS	C16:0	C18:0	C18:1	C18:2	IV	Carotene
**RL**	−0.21	0.45*	−0.11	0.04	0.03	0.07	0.10	−0.07
**HI**		0.10	−0.16	0.05	−0.09	0.38*	0.29*	−0.24
**PCS**			−0.13	0.24	−0.23	0.40*	0.18	−0.11
**C16:0**				−0.28*	−0.71*	− 0.10	−0.74*	− 0.30*
**C18:0**					−0.26	0.01	−0.22	−0.21
**C18:1**						−0.45*	0.47*	0.38*
**C18:2**							0.57*	−0.27
**IV**								0.17

*RL* Rachis length, *HI* Height increment, *PCS* Petiole cross section, *C16:0* Palmitic acid content, *C18:0* Stearic acid content, *C18:1* Oleic acid content, *C18:2* Linoleic acid content, *IV* Iodine value, *Carotene* Carotene content

*significant at *p* ≤ 0.05

**Table 3:** Pearson’s correlations between individual fatty acid contents and IV with vegetative parameters in 2.6–5 population

Population	2.6–5							
	HI	PCS	C16:0	C18:0	C18:1	C18:2	IV	Carotene
**RL**	−0.08	0.28*	−0.04	− 0.06	0.02	− 0.03	−0.02	0.31*
**HI**		0.45*	−0.19	0.37	−0.19	0.42*	0.32*	−0.04
**PCS**			−0.08	0.20	−0.12	0.28*	0.16	0.04
**C16:0**				−0.31*	− 0.71*	−0.05	− 0.71*	0.02
**C18:0**					−0.20	0.31*	−0.17	−0.07
**C18:1**						−0.62*	0.20	0.08
**C18:2**							0.56*	−0.12
**IV**								0.03

*RL* Rachis length, *HI* Height increment, *PCS* Petiole cross section, *C16:0* Palmitic acid content, *C18:0* Stearic acid content, *C18:1* Oleic acid content, *C18:2* Linoleic acid content, *IV* Iodine value, *Carotene* Carotene content

*significant at *p* ≤ 0.05

### BC_2_ consensus genetic map

**Fig. 1:**
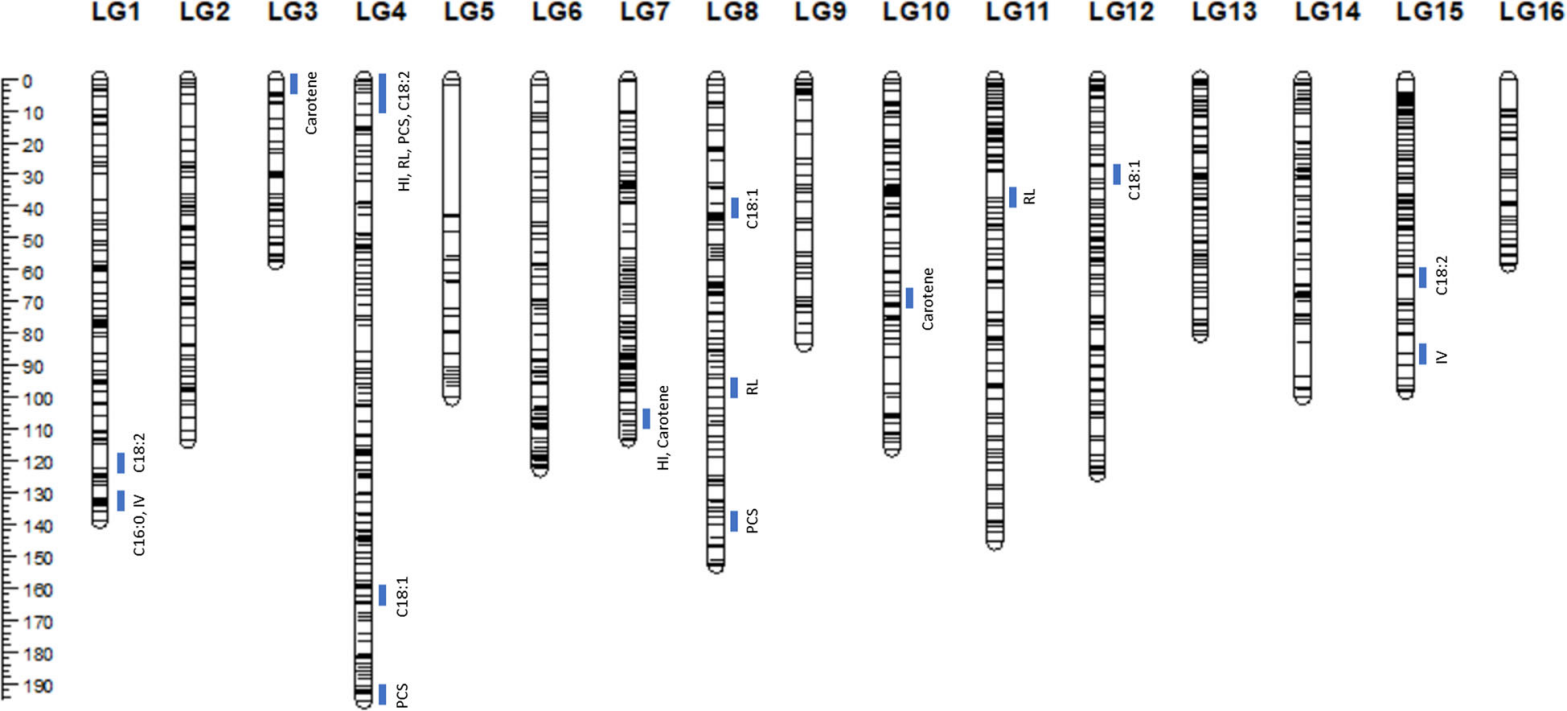
The integrated genetic map of the backcross 2 populations and distribution of QTLs associated with compactness traits and FAC

A total 4491 SNP markers were tested for polymorphism in both populations, and 515 and 715 SSR markers screened for informativeness in populations 2.6–1 and 2.6–5, respectively. Polymorphic markers with all segregation profiles (see Additional file 2) originating from both parental palms (BC_1_ and *E. guineensis*), which met the expected segregation ratios at *P* ≤ 0.05 and which had a nearest-neighbor stress value of less than 3 cM were considered suitable for use in constructing the genetic map (see Additional file 3). The consensus genetic map for both families is shown in Fig. 1, while Table 4 and Additional file 4 summarize the marker compositions and lengths of the individual linkage groups (LGs). The 2.6–1 and 2.6–5 genetic maps had 1744 and 1254 markers covering 1505 and 1564 cM, respectively. Both maps were integrated into the consensus map (see Additional file 5) of 16 LGs with 1963 markers (1814 SNPs and 149 SSRs), spanning a total map length of 1793 cM. The lengths of the individual LGs on the consensus map were 57–195 cM, with a mean of 112 cM. The average distance between markers was 0.91 cM. Initially, LG5 in population 2.6–1 and LGs 1 and 15 in population 2.6–5 had two sub-groups. However, they were successfully integrated into a consensus linkage group. The largest intervals between two loci were 9.62 cM (LG7), 14.86 cM (LG5) and 17.83 cM (LG6) for the consensus, 2.6–1 and 2.6–5 maps, respectively.

**Table 4:** Distribution of markers on the 16 linkage groups (LG) in the individual and integrated BC_2_ genetic maps

LG	Population 2.6–1			Population 2.6–5			Integrated		
	Map Length (cM)	No. Markers	Average Interval (cM)	Map Length (cM)	No. Markers	Average Interval(cM)	Map Length (cM)	No. Markers	Average Interval(cM)
**1**	115	121	0.95	88 + 23^a^	95 + 9^a^	1.06	138	146	0.95
**2**	101	87	1.16	97	62	1.56	113	107	1.06
**3**	55	48	1.15	58	42	1.38	57	54	1.06
**4**	174	178	0.98	212	117	1.81	195	202	0.97
**5**	25.2 + 14.8^a^	21 + 17^a^	1.05	53	22	2.41	100	44	2.27
**6**	106	129	0.82	126	82	1.54	122	135	0.90
**7**	78	173	0.45	85	117	0.73	113	189	0.60
**8**	122	132	0.92	175	97	1.80	152	154	0.99
**9**	82	56	1.46	37	15	2.47	83	57	0.53
**10**	88	129	0.68	93	93	1.00	116	138	0.84
**11**	128	166	0.77	133	125	1.06	145	179	0.81
**12**	108	130	0.83	124	91	1.36	124	144	0.86
**13**	77	127	0.61	57	96	0.59	80	135	0.59
**14**	98	77	1.27	88	69	1.28	99	94	1.05
**15**	69	119	0.58	52 + 46^a^	79 + 10^a^	1.10	98	142	0.69
**16**	50	34	1.47	63	33	1.91	58	43	1.35
**Total**	1505	1744	0.86	1564	1254	1.25	1793	1963	0.91

^a^Sub-groups

### QTLs associated with vegetative parameters, carotene and FAC

The results of the QTL analysis by Genstat are summarized in Table 5 and Additional file 6, and distribution of the QTLs on the respective LGs shown in Fig. 1. There were eight significant QTLs associated with height increment, rachis length and petiole cross section in the consensus map. The three traits in combination contribute to shorter and more compact palms. Interestingly, a single genomic locus in LG4 was associated with two of the traits – height increment and petiole cross section, with the QTL for rachis length in close proximity in the same LG. An additional QTL for petiole cross section was identified on the other end of LG4 for population 2.6–5 (Additional file 6), and it also appeared on the integrated LG4. The two QTLs for petiole cross section on LG4 were more than 150 cM apart, so they are likely not linked.

**Table 5:** QTLs associated with compactness traits and FAC identified via Genstat in BC_2_ integrated map

	BC_**2**_ Integrated Map				
	Closest marker to QTL peak	LG	Position (cM)	LOD	QTL Interval (cM)
**HI**	SNPM00563	4	4.3	7.77	0–17.4
	SNPM00010	7	111.9	3.76	101.5–113.3
**RL**	SNPM03201	4	11.3	3.79	8.1–17.4
	SNPM03772	8	92.6	3.17	81.6–99.7
	SNPM03676	11	38.7	4.17	37.4–47.4
**PCS**	SNPM00563	4	4.3	7.70	0–17.4
	SNPM02535	4	193.1	3.20	187.4–193.1
	sEg00213	8	139.9	3.81	137.9–147.2
**C16:0**	SNPM00796	1	133.4	3.59	132.5–133.9
**C18:1**	SNPM02507	4	169.2	3.44	169.2
	SNPM03249	8	44.0	3.19	44.0–47.5
	SNPM00274	12	31.4	5.97	22.0–34.2
**C18:2**	SNPM01602	1	124.9	4.62	122.0–138.8
	SNPM00249	4	3.4	8.19	0–17.4
	SNPM01190	15	70.3	5.14	58.2–98.1
**IV**	SNPM01452	1	132.5	13.02	122.2–138.8
	SNPM03285	15	98.1	5.98	77.1–98.1
**Carotene**	SNPM02349	3	4.4	5.12	0–22.9
	SNPM03960	7	108.5	3.73	107.4–113.3
	SNPM03921	10	77.4	3.63	63.7–77.4

*HI* Height increment, *RL* Rachis length, *PCS* Petiole cross section, *C16:0* Palmitic acid content, *C18:0* Stearic acid content, *C18:1* Oleic acid content, *C18:2* Linoleic acid content, *IV* Iodine value, *Carotene* Carotene content

In addition, nine QTLs were identified for iodine value and FAC, namely C16:0, C18:1 and C18:2 content across five LGs. Single QTLs for iodine value and C16:0 content were located around the same locus in LG1. Relatively high LOD levels were detected for some of the compactness and FAC traits – height increment (7.77), petiole cross section (7.70), C18:2 content (8.19) and iodine value (13.02). Interestingly, this study also revealed, for the first time, three QTLs associated with carotene content across three LGs.

### Common and population specific QTLs

**Fig. 2:**
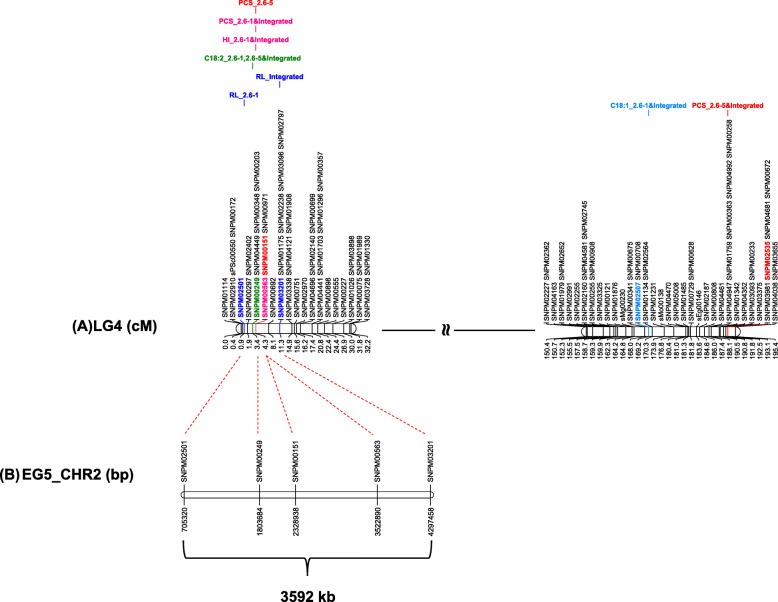
Corresponding regions of LG4 and pseudochromosome 2, showing a major genomic region influencing compactness and fatty acid composition in LG4

Figure 2a shows a major genomic region in LG4 influencing the vegetative parameters namely height increment, petiole cross section and rachis length in the populations. The closest marker to the QTL peaks for height increment and petiole cross section was the same, SNPM00563, both in the population 2.6–1 and consensus maps. Genstat revealed that the QTL peaks related to rachis length the consensus maps were detected in the same region, about 7 cM away from the QTL peaks for height increment and petiole cross section. Interestingly, the QTL for C18:2 content, one of the most abundant unsaturated fatty acids in *oleifera* and interspecific hybrids, also mapped around the same region for all the three maps. The SNP markers corresponding to the QTLs for height increment, petiole cross section, C18:2 content and rachis length were also physically positioned on the genome build, spanning about 3600 kb (Fig. 2b). This confirmed that the QTLs influencing the traits were in close proximity, suggesting a major genomic region influencing both compactness and oil unsaturation.

Apart from the population-specific QTL for petiole cross section in LG4, there were other population-specific QTLs (see Additional file 6), for example, a significant QTL for rachis length in LG8 for the population 2.6–1 and also detected in the consensus map. Similarly, there were also population-specific QTLs for height increment in LG7 and FAC (C18:1 and C18:2 content) reflecting the diversity of the two populations. The QTLs linked to carotene content were also specific to population 2–6-5 (and also detected in the consensus map), most likely because the variation for the trait was higher in population 2–6-5 (37.1%) than in 2–6-1 (31.2%) (Table 1).

The integrated consensus map proved useful in detecting QTLs not detected in the individual populations. An example was the QTL for C18:2 content on LG1 (confirmed as a minor QTL in the MapQTL analysis in Table 6), about 9 cM away from the QTLs for C16:0 content and iodine value. Interestingly, the consensus map, while maintaining the population-specific QTL for rachis length in LG4 and LG8, revealed a new QTL for it in LG11 not detected in the individual populations. The QTL in LG11 was also confirmed as to be minor by MapQTL analysis (Table 6). However, there was also a QTL detected in the genetic map of an individual population but not in the consensus map. This was the QTL associated with rachis length in LG13 in population 2.6–5 (Additional file 6), but it was not reproducible in the consensus map.

**Table 6:** QTLs detected using both Genstat and MapQTL

QTL	Marker	Segregation Type	Genestat^**a**^			MapQTL		
			LG	Position (cM)	LOD Score for Genestat	LOD threshold for MapQTL	LOD score	Adjusted Phenotypic variation^d^
HI	SNPM00563	aa x ab	4	4.3	7.77	3.4	5.69	30.7
	SNPM00010	aa x ab	7	111.9	3.76		4.36	29.7
RL	SNPM03201	aa x ab	4	11.3	3.79	3.3	3.0^b^	14.4
	SNPM03772	aa x ab	8	92.6	3.17		4.28	22.8
	SNPM03676	ab x aa	11	38.7	4.17		3.05^b^	16.4
PCS	SNPM00563	aa x ab	4	4.3	7.70	3.6	4.61	24.3
	SNPM02535	ab x ab	4	193.1	3.20		3.81	21.1
	sEg00213	ab x aa	8	139.9	3.81		3.94	24.2
C16:0	SNPM00796	aa x ab	1	133.4	3.59	3.6	4.20	28.7
C18:1	SNPM02507	aa x ab	4	169.2	3.44	3.3	4.33	29.8
	SNPM03249	ab x ab	8	44.0	3.19		4.25	29.3
	SNPM00274^c^	aa x ab	12	31.4	5.97		–	–
C18:2	SNPM01602	ab x ab	1	124.9	4.62	3.6	3.31^b^	21.6
	SNPM00249	ab x aa	4	3.4	8.19		5.55	34.2
	SNPM01190	ab x ab	15	70.3	5.14		3.80	26.1
IV	SNPM01452	ab x ab	1	132.5	13.02	3.5	8.93	49.5
	SNPM03285	aa x ab	15	98.1	5.98		8.51	39.8
Carotene	SNPM02349	ab x ab	3	4.4	5.12	3.7	3.9	26.0
	SNPM03960	ab x aa	7	108.5	3.73		3.75	31.7
	SNPM03921	ab x aa	10	77.4	3.63		4.20	27.7

*HI* Height increment, *RL* Rachis length, *PCS* Petiole cross section, *C16:0* Palmitic acid content, *C18:0* Stearic acid content, *C18:1* Oleic acid content, *C18:2* Linoleic acid content, *IV* Iodine value, *Carotene* Carotene content

^a^ Threshold for Genstat was LOD 3

^b^Significant chromosome-wide

^c^SNPM00274 considered not significant, as association with C18:1 not reproducible in MapQTL

^d^Percentage phenotypic variation for observed QTL corrected as in Methods

### Detection of QTLs via interval mapping (IM)

Interval mapping by MapQTL was done to corroborate the QTLs identified. The analysis was only on the integrated map and the results presented in Table 6 and Additional file 7. Generally, all the QTLs detected earlier (with only one exception) were also revealed as influencing the specific traits but with the added advantage that they were divisible into major (significant genome-wide) and minor QTLs (significant chromosome-wide). The QTLs for height increment in LG4 and LG7 as well as those for petiole cross section in LG4 and LG8 were major. The genomic region associated with rachis length in LG8 was also major. However, the QTLs for rachis length observed via Genstat in LG4 and LG11 were minor. For FAC, the QTLs linked to C18:1content (LG4 and LG8), C18:2 content (LG4 and LG15), C16:0 content (LG1) and iodine value (LG1 and LG15) were also major, and so too the QTLs linked to carotene content in LG3, LG7 and LG10. However, the QTL linked to C18:2 content in LG1 was only minor, pointing to a region that may be regulating the trait. The QTL linked to C18:1 content in LG12 was not reproducible in the MapQTL analysis and so not considered a locus associated with the trait. A point to note is that the QTLs revealed in the consensus, but not individual, maps – for C18:2 content (LG1) and rachis length (LG11) - were only minor. This clearly demonstrates that the detection power for minor QTLs is significantly enhanced in the integrated map.

The total phenotypic variation explained (PVE), adjusted for the small population size, is also indicated in Table 6. The major QTLs for vegetative parameters - petiole cross section, height increment and rachis length - were generally of intermediate to high effect, with PVE of 23–70%. The PVEs were 60% for height increment (two major QTLs), 23% for rachis length (one major QTL) and 70% for petiole cross section (three major QTLs). The combined effect of the three major QTLs for carotene content was also high with PVE of 85%. The PVEs for the major QTLs associated with FAC were medium to high (of 29–89%). The single major QTL for C16:0 content had 29%, while the two major QTLs for C18:1 and C18:2 content a cumulative effect of ~ 60%. The highest effect was for iodine value with the two major QTLs cumulatively giving 89%.

### Assessment of genotypes of markers associated with FAC, compactness and carotene content

**Fig. 3:**
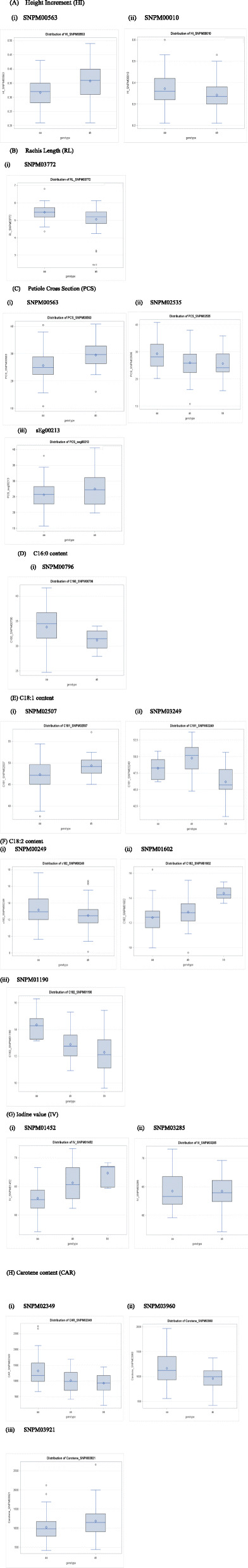
Boxplot showing means of phenotypes for major QTL were compared using the independent t-test for a marker with two genotypes, and Duncan’s test for markers with three genotypes (SAS 9.3 statistical package). Means of the different genotypes were significantly different at P ≤ 0.05. ((A) Height increment, (B) Rachis length, (C) Petiole cross section, (D) C16:0 content, (E) C18:1 content, (F) C18:2 content, (G) Iodine value, (H) Carotene content)

The genotypes of the closest markers to the QTL peak for the traits were examined to determine their allelic inheritance and effect on the traits (Table 6 and Fig. 3). The compactness traits - petiole cross section and height increment- have the same marker, SNPM00563, linked to them in LG4. The heterozygous allele was contributed by the male parent (a BC_1_ hybrid), and resulted, on average, in a larger petiole cross section and greater height increment (Fig. 3). The polymorphic allele for the second major QTL for height increment (LG7) was also contributed by the male parent but this resulted in lower height. The marker (SNPM02535) associated with the second major QTL for petiole cross section (also in LG4) was polymorphic in both parents, while marker SNPM03772, associated with the only major QTL for rachis length (LG8), was inherited from the interspecific male parent and resulted in lower rachis length.

Interestingly, three of the polymorphic alleles linked to major QTLs associated with FAC were also contributed by the male parent, while three markers were informative in both parents, and one polymorphic allele (C18:2 content in LG4) contributed by the female *E. guineensis* parent. The markers associated with the major QTL peaks for C16:0 content in LG1 (SNPM00796) and C18:1 content in LG4 (SNPM02507) were heterozygous in the male parent, where they caused, on average, higher C18:1 and lower C16:0 content. The marker associated with the QTL for iodine value in LG15 was also polymorphic in the male parent and it resulted in higher iodine value. The marker (SNPM00249) associated with C18:2 content in a separate region in LG4 was informative in the female *E. guineensis* parent, resulting in lower C18:2 content. The markers linked to carotene content in LG were either inherited from the female parent (*E.guineensis*) (LG7 and 10) or heterozygous in both parents (LG3).

### Candidate genes within QTL intervals

Candidate genes residing within the QTL intervals were identified using the existing oil palm genome assembly [28]. Blast results to the genome build identified 21 candidate genes associated with fatty acid synthesis and vegetative parameters within the QTL confidence intervals. The *ERECTA* gene [GenBank: XM_010910431] was found in the QTL interval linked to petiole cross section on LG4. In addition, the QTL region for height increment in LG7 also revealed an interesting gene with high similarity to the auxin transport protein *BIG* [GenBank: XM_010943964] in oil palm. Similarly, we identified *BAM1* [GenBank: XM_010914345] which co-localized with markers in the QTL region associated with rachis length on LG11. For FAC, two 3-ketoacyl-CoA synthase genes in Arabidopsis, *CUT1* [GenBank: XM_010917870] and *KCS11* [GenBank: XM_010916640] flanked the QTLs for iodine value and C18:2 on LG1. Details of all 21 genes identified are provided in Additional file 8. There were, however, no significant candidate genes corresponding to the genomic region associated with carotene content.

## Discussion

Oil palm interspecific hybrid breeding worldwide aims to develop compact palms with higher unsaturated oil without sacrificing yield. Higher yield can then be obtained by planting more of the palms per unit land area [29]. The breeding efficiency can be improved by linking markers to the desired traits and accumulating favorable alleles for them. This is a far better approach than directly targeting the yield parameters which are generally under polygenic control and strongly influenced by the environment, making it quite a difficult forest to navigate through with the current technology [30].

As expected, all the traits examined had values between *E. oleifera* and *E. guineensis*, similar to the observations in other studies [16, 31]. This adds confidence to the phenotypes observed. The wide distribution for all the traits measured suggests that both the BC_2_ populations are ideal for QTL mapping and selection and improvement in oil palm. Rachis length and height increment in both populations were considerably lower than in commercial DxP [27], signifying their potential in the development of compact palms.

Correlations between the traits are important information for breeding. The correlations between the different FAC parameters corroborate those from other studies [14, 16, 20], and suggest, as may be expected, that any increase in the oil unsaturation (IV) will reduce the saturated fatty acids, namely, C16:0 and C18:0 content. The inverse relationship between the two most abundant unsaturated fatty acids C18:1 and C18:2 content further implies that any increase in C18:1 content will likely overflow to C18:2 content. Similarly, the positive correlations of petiole cross section with rachis length and height increment indicate that reducing petiole cross section will give shorter and more compact palms. This fits with the assumption that a larger petiole cross section supports a larger/longer rachis. Interestingly, and as also observed in a previous study [32], there was generally no correlation between the vegetative measurements and FAC components. The exceptions were the positive correlations between height increment and petiole cross section with C18:2 content, and between height increment and iodine value. The latter correlation between height increment and iodine value was likely caused by C18:2 content which strongly impacts iodine value, especially in interspecific hybrids. Montoya et al. [32] did not observe any correlation between iodine value and C18:2 content with height in *E. guineensis*, although [15] had a positive correlation between iodine value and percentage pulp (a yield parameter) in selected *E. guineensis* families. Interestingly, in Jatorpha, [33] found a positive correlation between C18:2 content and seed weight. The results in this study generally indicate that breeding for compactness in oil palm interspecific hybrids will not affect most of the fatty acid contents, although the relationships between C18:2 content and some of the compactness parameters need to be further investigated. Interestingly, C18:2 differs from the other fatty acids in that it is assembled in the endoplasmic reticulum, while the rest (C16:0, C18:0 and C18:1) are assembled in the plastid [34].

**Fig. 4:**
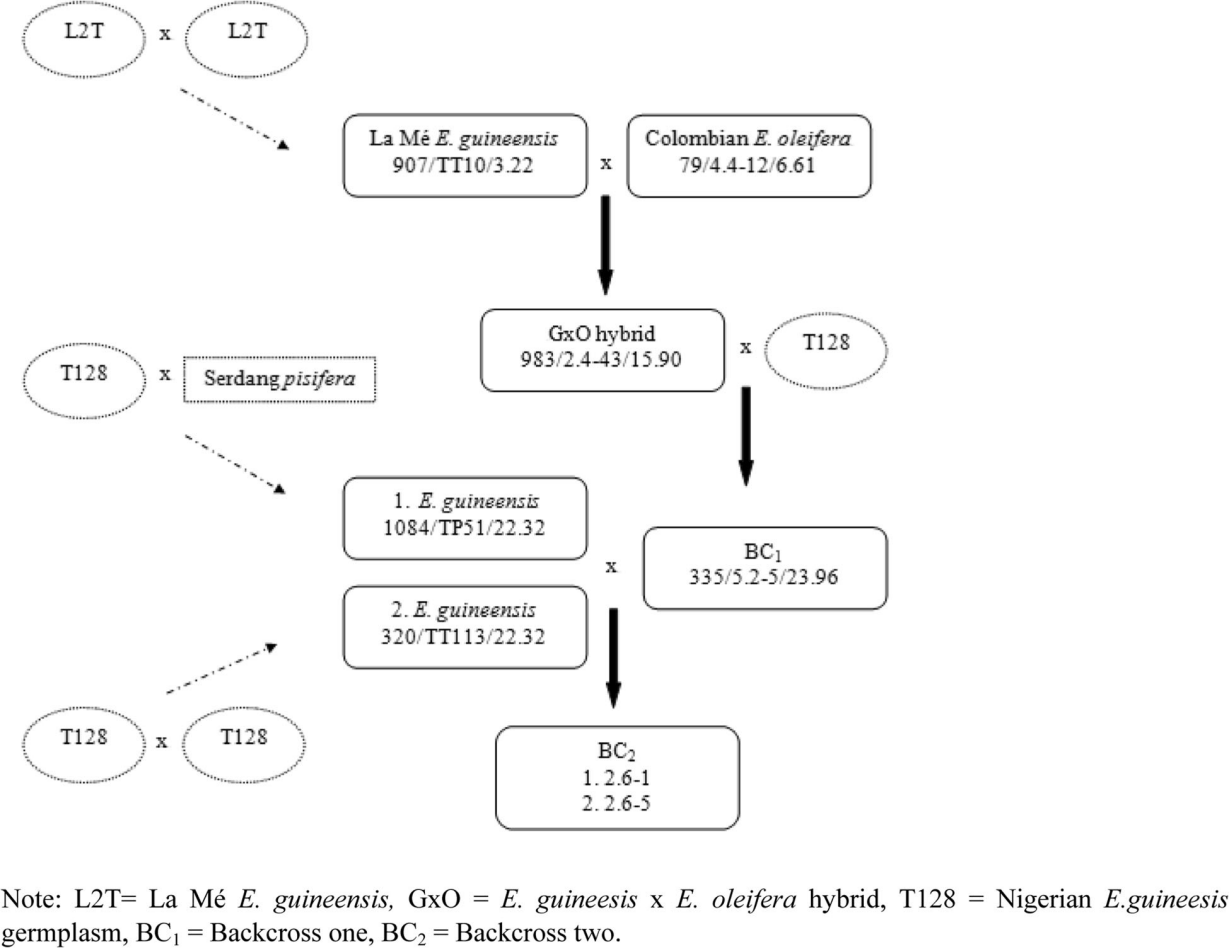
Breeding scheme of the two interspecific backcross two (BC_2_) mapping populations

The number of palms in an individual breeding trial are almost always smaller than in other crops, such being the quid pro quo for the size of the palm. The small number could have caused some of the QTLs to be missed and only allowed those with the most prominent effects to be detected. Vales et al. [35] found that the number of QTLs detected increased with the population size. Other factors, such as the phenotypic measurement accuracy and marker density, also contribute to QTL detection and localization [36]. To obviate the limitation of the small population, the map resolution and, hence, QTL detection power was improved by integrating the two individual BC_2_ genetic maps. This strategy was recommended by [16] for increasing the detection of QTLs in oil palm interspecific hybrids, building on the successful approach by [15] for *E. guineensis*. A total 149 SSR and 1814 SNP markers (1963 altogether) that met the expected segregation ratios and had a near-neighbor stress value below 3 cM, generated 16 LGs in the consensus map, consistent with the 16 chromosome-pairs in oil palm [37]. The genome length (1793 cM) was close to those reported by [14, 17] of 1815–1867 cM for *E. guineensis*. The average length of the LGs was 112 cM, in the range of most agricultural crops [38]. More importantly, there was generally a high collinearity among the common markers in the individual and consensus maps (Additional file 5). Some minor discrepancies in the marker order were to be expected due to differences in calculating the independent genetic maps [39]. The genome coverage of the integrated consensus map was much better than those by the independent maps in terms of the number of markers mapped. The average gap observed on the consensus map, 0.91 cM, was also much smaller than those previously reported on oil palm interspecific hybrids of 1.2–7.2 cM [14, 16, 17]. This was as expected, since the use of multiple parents increases the chances of identifying polymorphic markers at a specific genomic region, which, in turn, increases the effectiveness of uncovering the marker-trait association. The fact that the largest gap between markers did not exceed 20 cM further suggests that the markers were well distributed across the 16 linkage groups. The selfing of palm T128 to generate one of the two female parents used in this study (Fig. 4) also likely did not result in large homozygous blocks. The reason is because oil palm, being an outcrossing species, has high heterozygosity in its genome, especially palms like T128, which was from the wild in Nigeria [40].

To ensure the robustness of the genomic region linked to the traits, two independent QTL analyses (Genstat and MapQTL) were done, and only the QTLs detected by both were considered significant. Generally, a majority of the QTLs were detected by both methods, and they could be classified as major or minor. In the vegetative traits, the QTLs associated with petiole cross section and height increment were located at the same genomic region on LG4, in the independent genetic maps of both populations and consensus map, likely representing a major and stable locus influencing compactness. A major QTL peak associated with C18:2 content was located very close to the QTLs for petiole cross section and height increment (~ 1 cm away). Co-localization of the QTLs for different traits was not surprising, especially if the traits are correlated, and suggest pleiotropy with the genomic region containing a number of genes that influence several traits [41]. Population-specific QTLs were also identified, but only those reproducible in the consensus map by both the detection methods were considered significant. A case in point are the QTLs for petiole cross section and rachis length which were in close proximity in LG8 for population 2.6–1 and in the consensus map. Similarly, a minor QTL for rachis length was also found in LG4 of population 2.6–1 and the consensus map, close to the QTLs for height and petiole cross section in both populations. This provides further supporting evidence that the QTLs in LG4 are in a major region influencing all these three traits for compactness. The markers in the QTL intervals are useful for identifying favorable alleles for the characteristics desired. The population-specific QTLs were most likely due to the female parent, where in 2.6–1 it was a cross between an advanced breeding line (Serdang *pisifera*) and a germplasm from Nigeria (*tenera* palm, T128). The female parent in 2.6–5 was a self of T128. The slight difference in the genetic background of the female parents likely caused the phenotypic variability for the three traits in both populations, which contributed to some of the differences in QTL results from both populations. This explanation is also likely for the variability in the other population-specific QTLs, such as those for carotene content and FAC (C18:1, C18:2). Nevertheless, the population-specific QTLs can still be used to accumulate favorable alleles for oil unsaturation and compactness in crosses involving the populations. Similarly, population-specific QTLs were reported for pod-dehiscence in two families of soybean linked by a common parent [42]. Such specific QTLs are generally common in QTL analyses of bi-parental populations [43]. This suggests that the QTLs detected consistently across separate populations, as is the case in this study for petiole cross section, height increment, C18:2 content and iodine value, can be used with high confidence in marker-assisted breeding.

The marker SNPM00563 is significantly linked to two traits - petiole cross section and height increment - in LG4. From Table 6 and Fig. 3, it is obvious that it was inherited from the male interspecific parent and the polymorphic allele gave larger petiole cross section. This is expected as *oleifera* has larger fronds and petioles. Since the male parent also has variability in height increment, the marker also points to a genomic region contributing to increased height increment. Furthermore, since both traits are positively correlated, it is to be expected that the genomic region that influences petiole cross section will also influence height increment. The marker can thus potentially identify the individuals (in this case, those not carrying the heterozygous alleles) for optimizing the crosses to be made. Interestingly, the marker linked to the second major QTL for height increment in LG7 (SNPM00010) was also inherited from the male parent, but the polymorphic allele resulted, on average, in lower height. As lower height is also expected from the hybrid (due to the *oleifera*), the marker could be used to accumulate favorable genes for height increment.

To date, there are no QTL analysis of vegetative traits in interspecific hybrids. The QTLs detected in this study were compared to those described previously [15] for a segregating *E. guineensis* population. Most of the QTLs we detected were on different chromosomes from those by [15], with the exception of those for rachis length on LG11. This suggests that separate genomic regions influence compactness in *E. guineensis* and interspecific hybrids [18]. reported two QTLs related to rachis length and petiole cross section in *E. guineensis*. However, a comparison for similarity between the linkage groups could not be made as the sequence for restriction fragment length polymorphism (RFLP) markers linked to the traits were not published. More specifically on height increment, recent reports revealed QTLs and candidate genes influencing it in *E. guineensis* [10, 11]. However, the genomic region linked to height increment in this study was different from these two reports. Our results are consistent with an earlier study that compared the genetic architecture of FAC in both species of oil palm, where apart from some limited QTL loci that were common, the major QTL regions influencing FAC did not overlap and were species specific [32]. This suggests that the compactness and FAC traits are under the influence of different genomic regions in the two species.

Eight significant QTLs (seven majors, one minor) were detected for iodine value as well as C16:0, C18:1 and C18:2 content in four LGs in this study. Fewer were found than in previous reports on interspecific hybrids [16, 20] with 19 and 12, respectively. Five of the identified QTLs by us were similar to those in both the previous reports. The QTL for iodine value on LG15 agrees with that by [16]. The major QTLs for iodine value and C16:0 content on LG1 overlap with those by [20] which shows their potential to be used in breeding, at least on their genetic backgrounds. The consistency of our QTLs detected with those reported previously is proof of the quality of the FAC phenotypic data in this study. The detection of strong QTLs linked to C16:0 content, the most abundant saturated fatty acid, and iodine value, similar to in previous studies [16, 20], further supports the observation that the same genomic region has a major influence on the unsaturation (IV) and saturation (C16:0 content) of palm oil.

Three of the seven major QTLs associated with FAC resulted from markers informative in the male hybrid parent. Since higher unsaturation is to be expected from *oleifera*, it is interesting that the parental QTL marker alleles from the hybrid male parent (which has an *oleifera* component) - SNPM02507 (LG4) and SNPM03285 (LG15) - predicted on average for higher C18:1 content and iodine value, respectively. The third QTL peak denoted by marker SNPM00796 (LG1), also inherited from the male parent, predicted for lower C16:0 content (Fig. 3). Generally, the higher unsaturation of *E. oleifera* oil is also due to its alleviated C18:2 content, almost twice that in *E. guineensis* [38] oil. Interestingly, a major QTL for C18:2 content was located about 165 cM away from the C18:1 content QTL in LG4, suggesting that they are separate QTLs. The marker closest to the QTL peak for C18:2 content in LG4 (SNPM00249) was contributed by the female *E. guineensis* parent and resulted in lower C18:2 content. As the female parental lines only involved *E. guineensis*, it is fairly obvious that this particular QTL was responsible for the phenoytpic variation of the *E. guineensis* palms used (intra-phenotypic variation). The directional effect of the QTL is also consistent with the dominant effect observed for the *E. guineensis* alleles for C18:2 content [32]. As such, enriching the hybrid parental allele for markers related to C18:1 content and iodine value, and with the alternative allele for C18:2 content, will give palms with higher oil unsaturation.

Interestingly, markers for three of the major QTLs linked to FAC were polymorphic in both the female (*E. guineensis*) and hybrid male parent. These QTLs were specifically linked to the unsaturated fatty acids (C18:1 and C18:2) and iodine value (Table 6 and Fig. 3). It is thus impossible for these markers to accurately determine the source of the parental alleles in the offspring. However, as both parental lines (the female *E. guineensis* and hybrid male parent) had inherited alleles from palm T128, a Nigerian *E. guineensis* with less saturated oil (because of higher C18:1 content) [40], it is possible that even the *E. guineensis* contributed to the variability in FAC. As such, these QTLs could also likely account for the intra *E. guineensis* variation.

This study also detected the QTLs for carotene content. As such work has not been done before, no comparison could be made with other studies. Higher carotene is desirable because its pro-vitamin A and antioxidant activities enhance the nutritional attributes of palm oil [44]. The current palm oil has 500–700 ppm carotene content, while oleifera oil can have as much as 3000 ppm [45]. The individual BC_2_ palms had carotene content as high as 2600 ppm (Table 1). Of the three QTLs we detected, two were from the female parent (LG7 and 10), and the third heterozygous in both parents (LG3). The markers from the female parent predicted both for increased (SNPM03921, LG10) and reduced carotene content (SNPM03960, LG7), reflecting the variability of carotene content in the *E. guinnensis* oil. These markers can help select favorable alleles in future breeding for higher carotene content, apart from for compactness and more desirable FAC. Carotene content in the interspecific hybrids also appears to be under independent genetic regulation as there was a general lack of correlation with the compactness traits and FAC. Thus, breeders can select for higher carotene content and unsaturation without compromising compactness.

The phenotypic variation explained by the QTLs were corrected for the small population size as described by [32]. After the correction, the phenotypic variation explained by the major QTLs linked to individual fatty acids was 30–60%, similar to that reported by [32] who also adjusted for their limited population size. The highest PVE was for iodine value (89%), higher than in other studies [16, 32], but in line with [16] who found that the QTLs linked to iodine value gave the highest PVE among the traits evaluated. The generally medium to high PVEs for the QTLs associated with the individual fatty acids and iodine value, respectively, is also consistent with the high broad sense heritabilities reported for these traits in a BC_1_ hybrid, which ranged from 80% (C18:0, C18:1 and C18:2 content) to 90% (C16:0 content and iodine value) [32]. The high PVE for carotene content (85%) is similar to in the potato, where the QTLs had 71% PVE [46]. This is also consistent with the high broad sense heritability (78%) for carotene content in *E. guineensis* oil [47]. Since the heritabilities of several traits are similar in both species of oil palm, it is likely that the heritabilities for carotene content in *E. oleifera* and the hybrid oils are also high.

Although broad sense heritability can vary depending on the breeding material and environment in which it is evaluated, high values are consistently reported for the individual fatty acids and iodine value in both species of oil palm [48, 49]. Similarly, the PVE for markers linked to FAC in maize was as high as 83% [50], and the cumulative evidence thus far clearly suggest that the FAC traits in oil palm are under simpler genetic control and less polygenic than the yield traits. Similarly, high broad sense heritabilities of 30–80% have also been reported for the compactness traits (petiole cross section, height increment and rachis length) in both interspecific hybrids [51] and *E. guineensis* [52] oil palm. As such, the compactness traits are also likely influenced by fewer genes than the more complex yield traits. Basically, there is clear support for our observation that a few loci with large effects control FAC, compactness and carotene content in oil palm. As natural selection tends to fix the alleles of large effect during the adaptation process, presumably the traits evaluated will assist oil palm in its reproductive success [53, 54].

The medium to high heritabilities and PVEs for the QTLs in this study (for oil quality and compactness) are proof of their attractiveness for QTL analysis, as well as their amenability to selection via conventional and molecular breeding. However, PVE is often overestimated in most studies and may not be true in any breeding programme [55]. In fact, [56] cautioned that a small population can over- or underestimate PVE by as much as 19%, even with the correction for small populations (see Methods), as there is still the Beavis’ effect, which remains unadjusted [16, 57]. As such, caution must be exercised in expecting the gains from breeding.

Our small populations also explain why only the QTLs with medium to high effects were detected. Further, as demonstrated by [56], the chances of false negatives, i.e., not detecting the QTLs, increases the smaller the population below than 194, with the added risk that the QTLs detected may be as much as ~ 20 centimorgans (cM) from their actual positions. Another point of caution is that false positives increase with smaller populations, although they are more likely detected as minor QTLs, significant only chromosome-wide [56]. As such, the number of minor QTLs detected have to be treated with caution until validated in larger or other populations. The minor QTL detected for C18:2 content in LG1 was less than10cM away from the major QTLs associated with C16:0 content and iodine value, and it corresponded to almost the same genomic region previously linked to FAC in other studies [17], and so is likely a true QTL. As this study has demonstrated it possible to combine two families for marker-trait analysis, the integration can be easily extended to more BC_2_ families to increase the population size further for yet better detection of the QTLs and their positions. More importantly, as simulated by [58], increasing the number of palms will increase the detection of significant QTLs, especially those with small effects with PVEs less than 5%, which were missed in this study.

Knowledge of the oil palm genome [28] has allowed the underlying QTL intervals in the integrated map to be positioned on the EG5 physical map to identify potential candidate genes influencing the traits of interest. Even a 10 cM region in a genetic linkage map can contain hundreds of genes [28, 59], and this study only focussed on the selected genes (21of them) that had high similarity to known genes that impact fatty acid synthesis or have been associated in the literature with vegetative development.

The auxin transport protein, *BIG*, located in the QTL region for height, is required for auxin efflux and polar auxin transport (PAT) and could influence auxin-mediated developmental responses, e.g., cell elongation, apical dominance, lateral root production, inflorescence architecture, general growth and development [60]. Generally, *BIG* controls elongation of the pedicel and stem internodes through auxin action, which supports its role in regulating height in oil palm. In addition, *BIG* also plays a role in Arabidopsis in regulating its response to phytohormones, such as auxins, cytokinins, ethylene and gibberellic acid (GA), particularly during light-mediated stimuli, e.g., shade avoidance and etiolation [61, 62]. *BAM1*, found in the QTL region for rachis length, encodes a leucine-rich repeat receptor-like serine/threonine-protein kinase and is known to regulate cell division and differentiation, such as in the formation of shape, size and symmetry of leaves [63], suggesting its possible influence on rachis length. *ERECTA*, linked to petiole cross section on LG4, regulates the aerial architecture (including inflorescence), e.g. shoot apical meristem-originating organ shape, elongation of internodes and pedicels, adaxial-abaxial polarity and stomatal patterning, probably by tuning cell division and expansion [64], which explains how it may control the petiole size in oil palm.

In FAC, the *KCS11* gene on LG1 is associated with the QTLs for iodine value and C18:2 content. It is involved in fatty acid biosynthesis in both the saturated and mono-unsaturated acyl chains, C16 - C20 [65]. As such, it is interesting that the gene is located in the QTL interval regulating both saturated and unsaturated fatty acids in oil palm. The genomic region corresponding to the QTL intervals associated with carotene content did not contain any candidate genes associated with the carotenoid pathway, which is well characterized in plants [66]. A similar observation was made by [46, 67], where the QTL intervals associated with carotene content did not contain any known carotenoid gene, suggesting the complexity of the mechanism regulating carotenoid biosynthesis in plants.

Although the candidate genes in the QTL intervals are interesting, it is, however, important to keep in mind that their involvement and influence in controlling compactness and FAC of oil palm are still speculative. Further studies are necessary to determine their functions in regulating the traits.

## Conclusion

This study developed a SNP- and SSR-based dense integrated consensus genetic map of two oil palm BC_2_ populations, where the marker order was generally consistent with those in the independent maps. This is an important resource for future genetic mapping, QTL and molecular breeding applications, especially in oil palm interspecific hybrids. The 1814 SNPs and 149 SSRs in the consensus map are easily transferable for use by other research groups, indicating their potential in oil palm genetics research. A major genomic region influencing compactness was identified in LG4, while other QTLs linked to the compactness traits were identified in LG7 and LG8. These are the first QTLs reported for vegetative parameters in interspecific oil palm hybrids, and can facilitate better understanding of the genetic control of diverse palm traits. A multi population analysis is an effective approach to improve the statistical power for detecting QTLs. The detection of major QTL regions for FAC, similar to those reported previously, and the uncovering of new QTLs for FAC are testament to the usefulness of the multi population approach. Admittedly, due to the small populations studied, only the QTLs with medium/high effects were uncovered. Nevertheless, the traits studied were those with high heritabilities, and, together with the medium/high PVEs obtained for the QTLs, suggests that they are under strong genetic influence and, as such, amenable to improvement by breeding. The identification of favourable alleles related to compactness and FAC in the interspecific hybrid male parent, most likely from the *oleifera* ‘blood’, is an important step in the selective introgression of *oleifera* genes into *E. guineensis*. The QTL-containing linkage groups were successfully aligned with the oil palm genome build, and the high collinearity between the positions of the markers in the genetic and physical maps provided further confidence on the quality of the consensus map. Interesting genes were subsequently identified in the QTL intervals for the compactness traits and FAC, and they will be good candidates for future research and validation by gene expression analysis. The QTL markers and candidate genes in the QTL intervals for height increment, rachis length and petiole cross section can facilitate the breeding for compact palms, at least by using the genetic backgrounds employed in this study. This can prolong the economic lifespan of the palms, and allow higher planting density to increase the yield per area. This could be the answer to the limited land available for further expansion.

Furthermore, lower height, shorter rachis and smaller petiole cross section are preferred since more nutrients can be channeled into FFB production instead of vegetative growth [27]. In fact, compact palms at a density of 180/ha are being touted [68] for a possible 20% increase in yield. In addition, reducing saturated and increasing unsaturated FAs will open up the prospects for oil palm to compete more effectively with other oil crops, such as soybean, rapeseed and sunflower, in the liquid oil sector [69]. There are already efforts to alter the FAC of palm oil both through traditional breeding [70] and genetic engineering [71], but these approaches are still very much in their infancy. Identifying the QTLs associated with FAC and the resulting candidate genes, can prove useful for selection or genetic manipulation in the quest. The value of the palms will be even more significantly enhanced if higher levels of carotene content are also incorporated into compact palms with higher unsaturated oil, creating a new generation of oil palm planting material.

## Methods

### Mapping populations

The two mapping families studied were, BC_2_, 2.6–1 and 2.6–5, of 74 and 80 palms, respectively. They were in a trial (one location) with random block design (16 palms/plot) at United Plantations, Teluk Intan, Perak, Malaysia, planted in the year 2000. Population 2.6–1 was a cross between an *E. guineensis* female parent (Code 1084/TP51/22.32) (palm T128, Nigerian germplasm x a Serdang *pisifera*) anda BC_1_ palm (Code 335/5.2–5/23.96). Population 2.6–5′ was also a cross between a female *E. guineensis* (Code 320/TT113/22.32) (self of palm T128), with the same BC_1_ palm (Code 335/5.2–5/23.96). The BC_1_ palm itself was a cross between a GxO F1 hybrid (Code 983/2.4–43/15.90) and T128. The GxO F1 hybrid was a interspecific cross of La Mé *E. guineensis* (Code 907/TT10/3.22) and Colombian *E. oleifera* (Code 79/4.4–12/6.61). Their genesis is illustrated in Fig. 4. The mapping families were established and maintained by United Plantations Bhd, and were used in this study with permission from the company.

### Vegetative measurements and fatty acid composition

One-time measurement of the vegetative traits was done on all the individual palms in both populations at 8 years after planting. Determining the chronological frond order (or number, Frond 1 is the youngest fully-opened frond, Frond 2 the second youngest, etc.) is essential for measuring the traits – rachis length, petiole cross section and height increment. The fronds of an oil palm are produced in spirals, the more obvious (and steeper) one having eight parastichies (rows). It can run in any direction, clockwise or anti-clockwise, and is used in identifying the frond number. The second, less obvious (and shallower) one has five parastichis and runs in the opposite direction. It is not used in frond sampling, so irrelevant here. The parastichis produce a new frond in turn. If, say, Parastichy A produces a new frond today (Frond 1 today), and in time produces a second (after all the other parastiches have had their turn), the new frond would be the current Frond 1 and the previous one Frond 9, i.e., 1 + 8 = 9 [72].

The vegetative traits are measured on Frond 17, roughly the middling frond in the canopy of ~ 35 fronds, so representative of the crown physiological functions. The frond is easily identified by identifying Frond 1 and then running down its parastichy two notches, i.e., 1 + 8 + 8 = 17 [73, 74]. For convenience, height is measured to the base of Frond 41 – lower and easier to reach than Frond 17 – as a palm can be very tall. It is also easier to access, being below the bunches borned. Frond 41 is easy to identify, by following Frond 17 a further three notches down the parastichy, i.e., 17 + 8 + 8 + 8 = 41. It really does not matter which frond is used to measure height has it per se is of little interest. The data sought is height increment, and as palm trunks only show growth 2–3 years after planting, height increment (HI) is determined as follows:

- HI = Palm height at year T/T-2, where T is age of trial when the measurement is made.

Thirteen palms from both populations had died before the vegetative measurement. FAC and carotene content of the mesocarp oil was determined using the MPOB Test Method [75], where 20 fruits, randomly selected from a ripe bunch are analysed. A total 112 palms from both populations were analysed for their oil FAC and carotene content, the others having died or had no bunches. The FAC and carotene content analyses started when the palms were 12 years after planting (at optimal production). About 20% of the palms (16 for 2.6–1 and 18 for 2.6–5) had more than one bunch analysed to assess repeatability of the analyses. The data from three palms were omitted from the final QTL analysis on FAC and carotene content as they had seriously declined in yield (data not shown) due to a severe infection by the fungus, *Ganoderma*, which may have affected their oil quality. Their vegetative measurements were, however, used, being taken 4 years earlier when they were likely not yet afflicted.

### DNA extraction, PCR programme and genotyping

Spear leaves (the unopened fronds, just before becoming Frond 1) were harvested from each palm, including the parental palms, for DNA extraction by the modified CTAB method [76]. SSR analysis was carried out using the primers described in [77, 78], with the following PCR parameters: pre-denaturation at 95 °C for 1 min, denaturation at 95 °C for 30 s, annealing (temperature depending on primer) for 30 s, and extension at 72 °C for 30 s. The programme was run 35 cycles, followed by a final extension at 72 °C for 5 min. SNP analysis was also as described by [77, 78]. The SNP and SSR data were analysed according to [20, 77, 78]. A total 515 and 715 SSR markers were genotyped on populations 2.6–1 and 2.6–5, respectively, and 4451 SNPs and an additional 40 candidate SNPs flanking various fatty acid and oil biosynthesis-related genes also genotyped them using the Illumina Infinium assay and iPLEX respectively.

### Development of linkage map

As both the populations were not true BC_2_, the double pseudo test cross strategy proposed by [79] was used to analyse the segregating markers. The genetic linkage maps were first constructed separately for each population using JoinMap 4.1 [80]. Data sets in the independent populations were coded according to the cross-pollinator (CP) format. Only markers with less than 5% missing data and following the Mendellian ratios (*P* ≤ 0.05) were used in the map construction. For each population, the two parental maps and the integrated map were constructed concurrently using the maximum likelihood (MLM) method [81]. Default parameters (CP population, minimum LOD threshold of 1.0, recombination fraction threshold of 0.4, ripple value of 1.0 and jump threshold of 5.0) were used to assign markers to the individual linkage groups. Haldane’s mapping function was then used to translate the recombination frequencies between markers into centimorgan (cM) map distances. The markers showing nearest-neighbour stress of more than 3 cM were omitted after map construction, as they could have represented unlikely genotypes, such as those with double recombination within a short genetic distance [17]. Subsequently, the 2–6-1 and 2–6-5 genetic maps were integrated into a single consensus map using the common markers, by extending to multiple populations, the method used to order markers in a single population [81]. Calculation of map distance was done using the multipoint maximum likelihood in GenStat 18 [82].

### QTL analysis

The QTL analysis was by Genstat 18th edition [82] with the threshold set as LOD 3, and using the default parameters (cross-pollinator population and simple interval mapping) with effective number of independent test sets defined by [83]. The QTL analysis for the integrated map was also carried out using MapQTL 5 [84]. Interval mapping was used for QTL detection using the maximum 5 neighboring markers and 1 cM mapping size. The analysis for the individual populations assumed four QTL alleles (two parents per population). Markers associated with the QTLs heterozygous in the two female parents (*E. guineensis*) and/or the common male parent (BC_1_ interspecific hybrid) were considered informative in both populations. The QTLs had to be detected on the same genomic location, denoted by the same marker across the integrated map and two independent populations, with the genetic effect in same direction, to be considered the same. Markers linked to QTLs heterozygous in only one of the female *E. guineensis* parent was considered population-specific.

The QTL thresholds (genome-wide and chromosome-wide) were determined with 1000 permutation tests. Confidence interval for a QTL was determined using the standard method of LOD-1, while the phenotypic variation was corrected, as described by [32], by multiplying the explained variance with:

$$ 1-\left[\ \frac{1}{2 xLn\ (10)x\  LOD}\right] $$

The correction was to overcome the overestimation of phenotypic variance in small populations [35, 56].

### Assessing phenotype value for markers linked to QTLs and search for candidate genes in QTL intervals

Using SAS version 9.3, the t-test and Duncan’s analysis were carried out to compare the phenotypic values of the different genotypes. The palms were grouped by their genotypes, and their phenotypic values averaged for each genotype. The genomic region corresponding to the QTL confidence interval on the integrated map was extracted from the oil palm genome build (EG5) [28] and searched for sequence similarity (BLASTN and BLASTX) against the NCBI databases. Sequences with significant similarity (BLASTN e-value <1e-25 and 90% identity over total sequence length) to the genes of interest were shortlisted for further analysis. Putative biological function of the selected genes were derived from *UniProt,* a freely accessible database of protein sequence and functional information, and the literature.

## Supplementary information

**Additional file 1.**

**Additional file 2.** Segregation profiles of SSR and SNP markers in BC_2_ mapping populations. Horizontal bars are marker profiles observed.

**Additional file 3.** No. SSR and SNP markers used and excluded from construction of genetic map for populations 2.6–1 and 2.6–5.

**Additional file 4.** Marker positions on individual linkage groups in the consensus genetic map of populations 2.6–1 and 2.6–5.

**Additional file 5.** Map integration (consensus map) of populations 2.6–1 and 2.6–5 for genetic linkage groups 1–16.

**Additional file 6.** List of QTL associated with the compactness traits and FAC in the independent and the integrated maps determined via Genstat. For a particular trait (Ex. RL and C16:0), a number of markers are present in the QTL interval. Although almost the same genomic region (determined via position on LG) is linked to the QTL in independent populations, a different marker is at times located closest to the QTL peak, in the integrated map. The original marker identified in the independent populations remains significant.

**Additional file 7.** Significant major QTLs detected for respective traits using MapQTL. Horizontal line indicates the 95% genome wide significant threshold value for declaring a QTL.

**Additional file 8.** Blast results to the genome build identified 21 candidate genes within the QTL confidence intervals affecting the vegetative traits and FAC.

**Additional file 9.** List of the 1814 SNP markers on the integrated BC2 map, with their relevant sequence and variant call information.

**Additional file 10.** Detailed information on the 149 SSR markers on the integrated BC2 maps.

## Data Availability

All data generated or analysed in this study are included in this article [and its supplementary information files]. Relevant information on the SNP and SSR markers is also provided in Additional files 9 and 10 respectively. The SNP information has also been deposited at European Variation Archive (EVA) (Project: PRJEB39400Analyses: ERZ1466909). Sequences for the SSRs developed in this study has been deposited in GenBank (accession number MT682145- MT682200, as indicated in Additional file 10). The sequence information for the SNP and SSR markers is also available at http://genomsawit.mpob.gov.my (in download section).

## Abbreviations

BC_2_: Interspecific backcross two
FAC: Fatty acid composition
MPOB: Malaysian Palm Oil Board
QTL: Quantitative trait loci
BC_1_: Interspecific backcross one
RL: Rachis length
HI: Height increment
IV: Iodine value
PCS: Petiole cross section
C18:1: Oleic acid
C18:0: Stearic acid
C18:2: Linoleic acid
C16:0: Palmitic acid
LG: Linkage group
SNP: Single nucleotide polymorphism
SSR: Simple sequence repeat
MAS: Marker-assisted selection
LOD: Logarithm of odds ratio
EG5: Oil palm genome build
RFLP: Restriction fragment length polymorphism
PAT: Polar auxin transport
GA: Gibberellic acid
VLCFAs: Very-long-chain fatty acids
FFB: Fresh Fruit Bunches
