# Supplementary material for: An integrated linkage map of interspecific backcross 2 (BC2) populations reveals QTLs associated with fatty acid composition and vegetative parameters influencing compactness in oil palm
Source: BMC Plant Biol. 2020 Jul 29;20:356. doi: 10.1186/s12870-020-02563-5 (PMC7391521; doi:10.1186/s12870-020-02563-5)
Supplement: Supplementary file 1 — Additional file 1. [file 12870_2020_2563_MOESM1_ESM.docx]

(A) Rachis Length

(i) Population 2.6-1


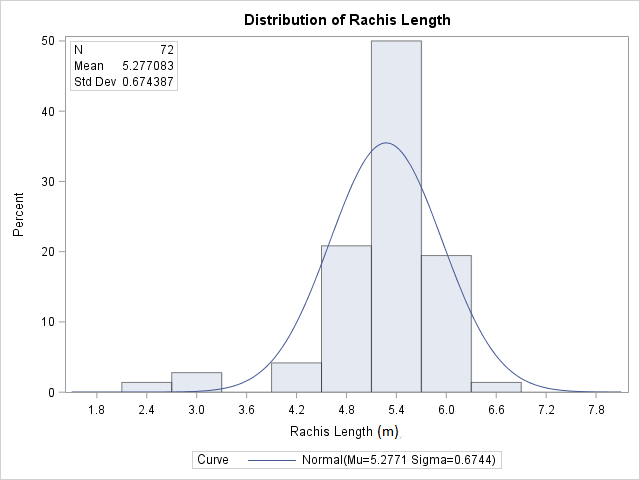


(ii) Population 2.6-5


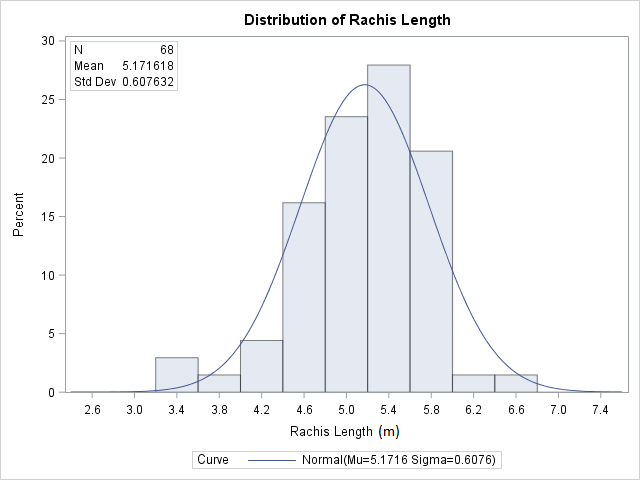


(B) Height Increment

(i) Population 2.6-1


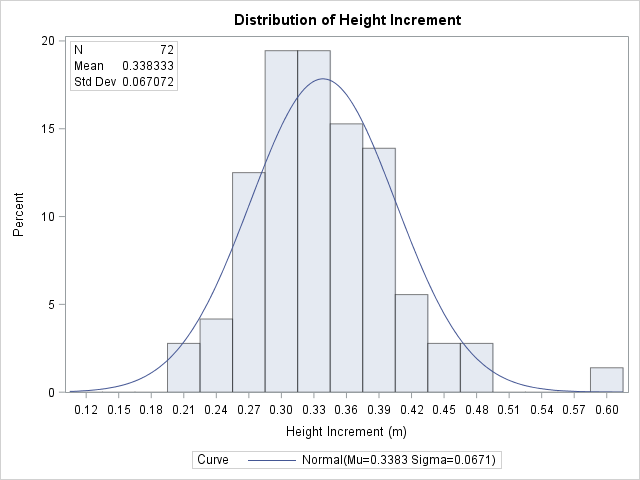


(ii) Population 2.6-5


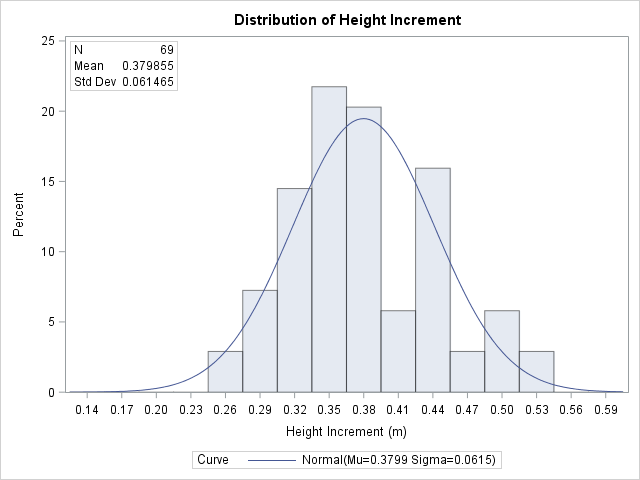


(C) Petiole Cross Section

(i) Population 2.6-1


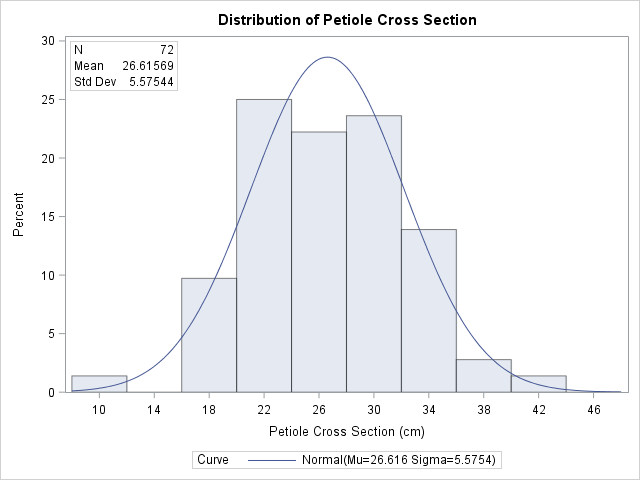


(ii) Population 2.6-5


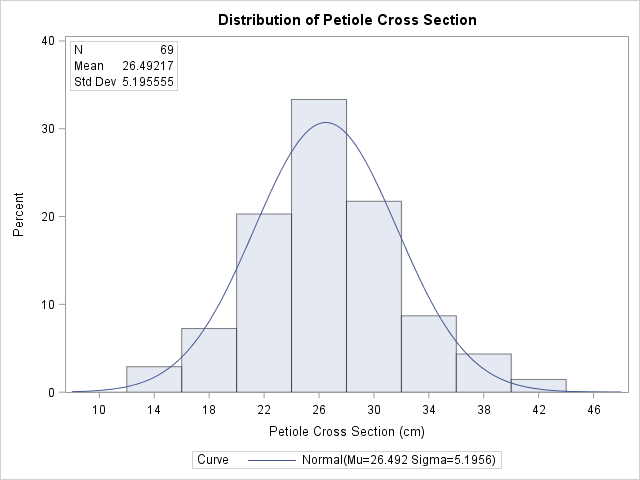


(D) C16:0 content

(i) Population 2.6-1


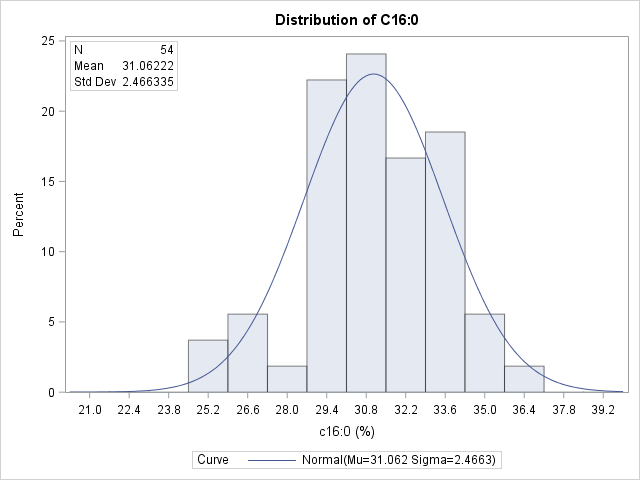


(ii) Population 2.6-5


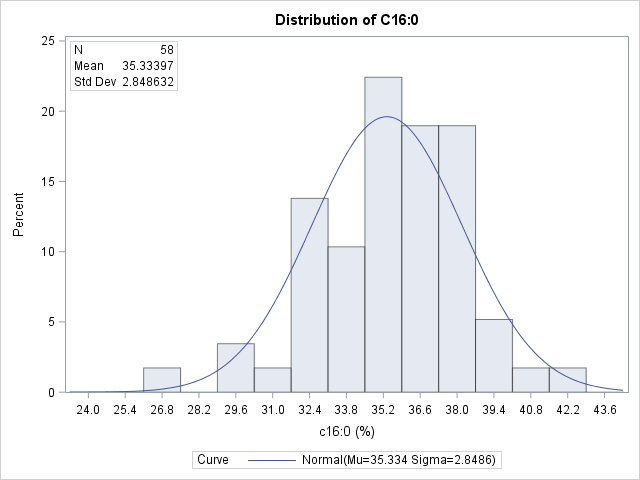


(E) C18:0 content

(i) Population 2.6-1


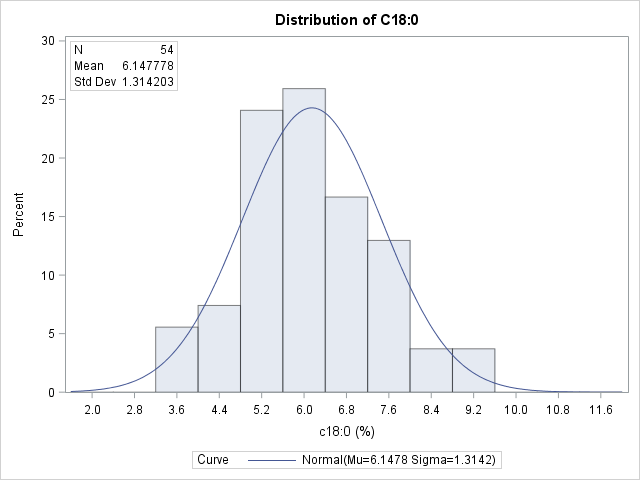


(ii) Population 2.6-5


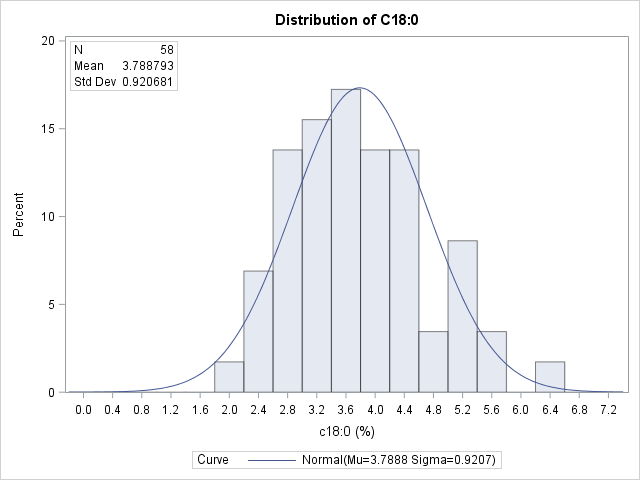


(F) C18:1 content

(i) Population 2.6-1


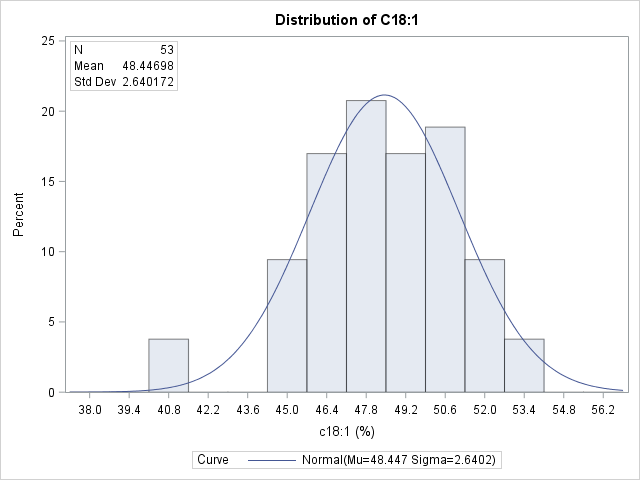


(ii) Population 2.6-5


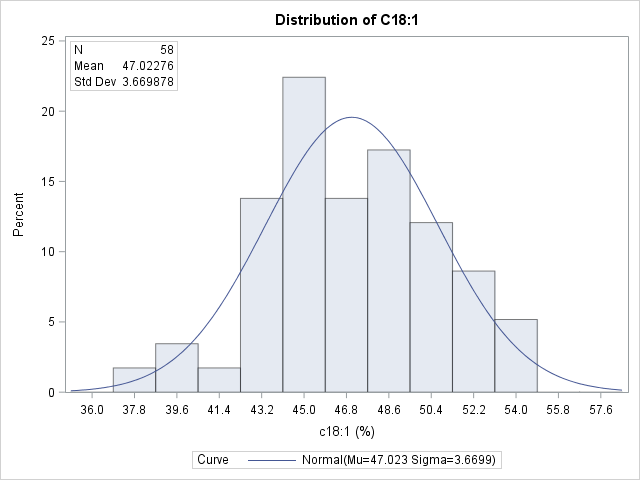


(G) C18:2 content

(i) Population 2.6-1


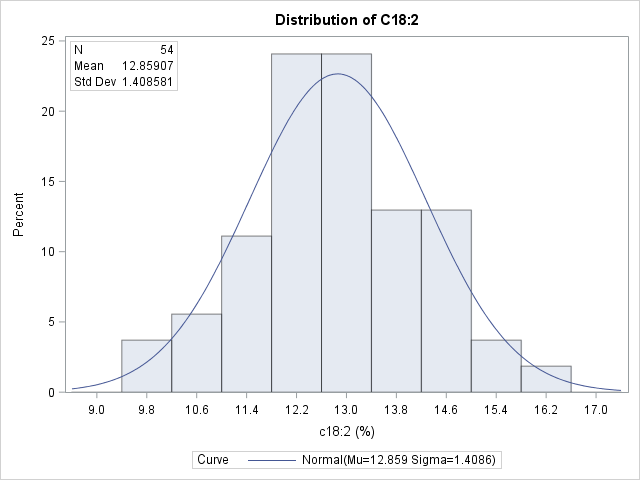


(ii) Population 2.6-5


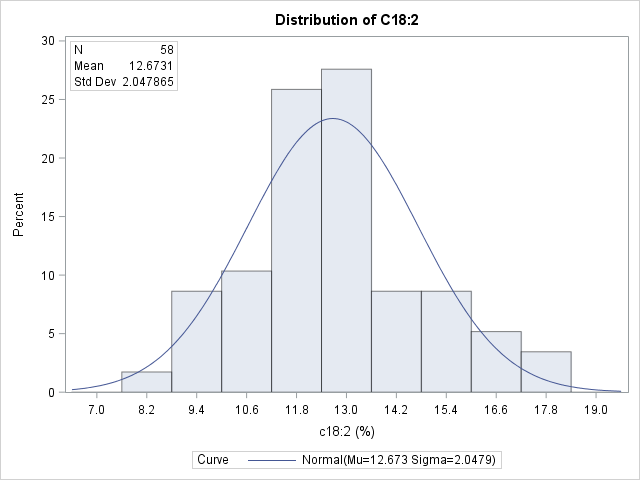


(H) Iodine Value

(i) Population 2.6-1


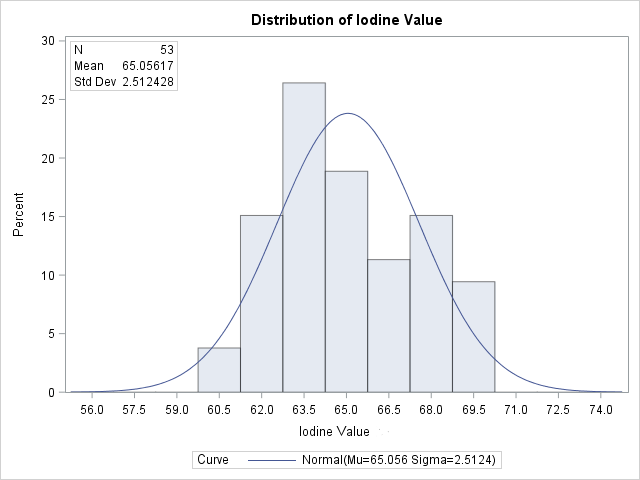


(ii) Population 2.6-5


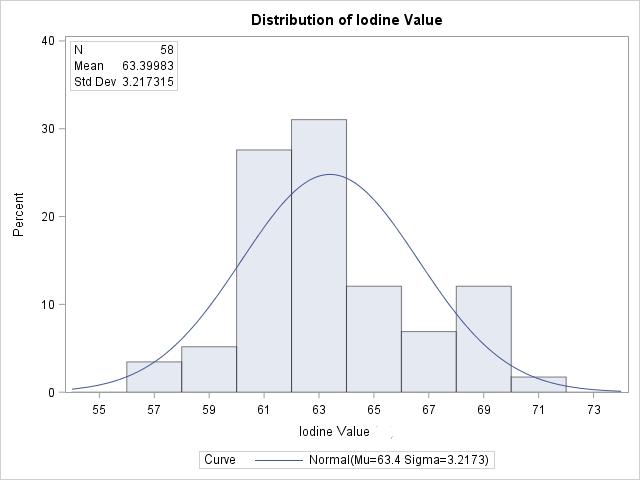


(I) Carotene Content

(i) Population 2.6-1


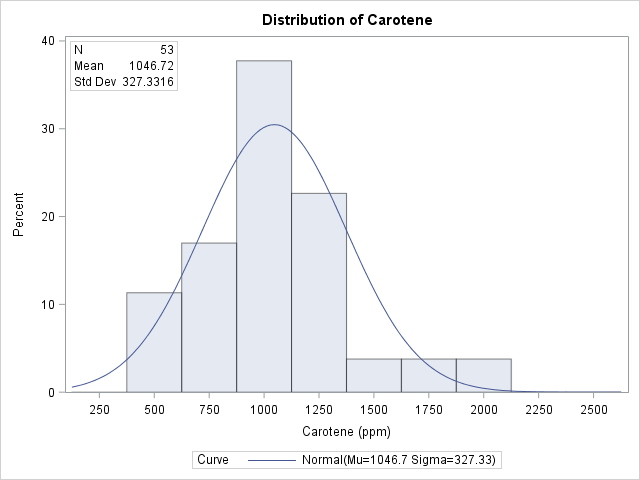


(ii) Population 2.6-5


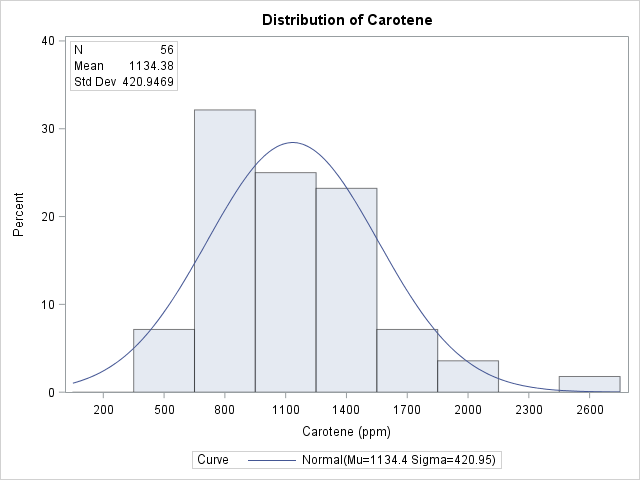


Additional File 1 : Histogram showing distribution of traits in 2.6-1 and 2.6-5 mapping populations: (A) Rachis Length, (B) Height Increment/year, (C) Petiole Cross Section, (D) C16:0 content, (E) C18:0 content, (F) C18:1 content, (G) C18:2 content, (H) Iodine Value (I) Carotene Content.
