# Supplementary material for: An integrated linkage map of interspecific backcross 2 (BC2) populations reveals QTLs associated with fatty acid composition and vegetative parameters influencing compactness in oil palm
Source: BMC Plant Biol. 2020 Jul 29;20:356. doi: 10.1186/s12870-020-02563-5 (PMC7391521; doi:10.1186/s12870-020-02563-5)
Supplement: Supplementary file 2 — Additional file 2. Segregation profiles of SSR and SNP markers in BC2 mapping populations. Horizontal bars are marker profiles observed. [file 12870_2020_2563_MOESM2_ESM.docx]

Additional file 2: Segregation profiles of SSR and SNP markers in BC_2_ mapping populations. Horizontal bars are marker profiles observed.

|  | **No. alleles** | **Parental genotypes** | | **Progeny genotypes** | **Expected segregation ratio** | **No. segregating markers** | |
| --- | --- | --- | --- | --- | --- | --- | --- |
|  |  | **Female** | **Male** |  |  |  |  |
|  |  |  |  | **1 2 3 4** |  | **SSR** | **SNP** |
| **1** | **Single allele (Dominant)** |  |  |  | **1:1** | **30** | **939** |
| **2** | **Single allele (Dominant)** |  |  |  | **1:1** | **83** | **1275** |
| **3** | **Two alleles (Codominant)** |  |  |  | **1:2:1** | **11** | **629** |
| **4** | **Three alleles (Codominant)** |  |  |  | **1:1:1:1** | **27** | **-** |
| **5** | **Four alleles (Codominant)** |  |  |  | **1:1:1:1** | **3** | **-** |
