# Supplementary material for: An integrated linkage map of interspecific backcross 2 (BC2) populations reveals QTLs associated with fatty acid composition and vegetative parameters influencing compactness in oil palm
Source: BMC Plant Biol. 2020 Jul 29;20:356. doi: 10.1186/s12870-020-02563-5 (PMC7391521; doi:10.1186/s12870-020-02563-5)
Supplement: Supplementary file 3 — Additional file 3. No. SSR and SNP markers used and excluded from construction of genetic map for populations 2.6–1 and 2.6–5. [file 12870_2020_2563_MOESM3_ESM.docx]

Additional file 3: No. SSR and SNP markers used and excluded from construction of genetic map for populations 2.6-1 and 2.6-5

|  | **2.6-1** | | | **2.6-5** | |
| --- | --- | --- | --- | --- | --- |
| **Markers used** | **SSR** | | **SNP** | **SSR** | **SNP** |
| No. markers | 61 | | 1683 | 93 | 1160 |
| **Markers excluded** | **SSR** | **SNP** | | **SSR** | **SNP** |
| No. monomorphic markers | 299 | | 2572 | 532 | 3052 |
| No. markers showing segregation distortion | 37 | | 227 | 82 | 282 |
| No. markers with nearest-neighbour stress >3cM | 118 | | 9 | 18 | 9 |
| **Total** | **515** | | **4491** | **715** | **4491** |
