# Supplementary material for: An integrated linkage map of interspecific backcross 2 (BC2) populations reveals QTLs associated with fatty acid composition and vegetative parameters influencing compactness in oil palm
Source: BMC Plant Biol. 2020 Jul 29;20:356. doi: 10.1186/s12870-020-02563-5 (PMC7391521; doi:10.1186/s12870-020-02563-5)
Supplement: Supplementary file 4 — Additional file 4. Marker positions on individual linkage groups in the consensus genetic map of populations 2.6–1 and 2.6–5. [file 12870_2020_2563_MOESM4_ESM.docx]

Additional file 4: Marker positions on individual linkage groups in the consensus genetic map of populations 2.6-1 and 2.6-5.

| Marker | Linkage Group | Position (cM) |
| --- | --- | --- |
| SNPM00153 | LG1 | 0.0 |
| SNPM01597 | LG1 | 0.0 |
| SNPM02398 | LG1 | 0.4 |
| SNPM03948 | LG1 | 2.2 |
| SNPM00932 | LG1 | 3.1 |
| SNPM00618 | LG1 | 3.1 |
| SNPM00165 | LG1 | 3.5 |
| SNPM01086 | LG1 | 5.3 |
| SNPM02612 | LG1 | 9.6 |
| SNPM00333 | LG1 | 11.6 |
| SNPM01028 | LG1 | 12.2 |
| SNPM00243 | LG1 | 14.0 |
| SNPM03737 | LG1 | 14.0 |
| SNPM02330 | LG1 | 14.5 |
| SNPM04153 | LG1 | 14.5 |
| SNPM00624 | LG1 | 14.5 |
| SNPM04152 | LG1 | 14.5 |
| SNPM02389 | LG1 | 17.1 |
| SNPM02787 | LG1 | 21.1 |
| SNPM03980 | LG1 | 21.1 |
| SNPM00566 | LG1 | 24.3 |
| SNPM00995 | LG1 | 26.0 |
| SNPM04828 | LG1 | 27.3 |
| SNPM04321 | LG1 | 29.5 |
| SNPM00780 | LG1 | 29.5 |
| SNPM04623 | LG1 | 29.5 |
| SNPM02102 | LG1 | 29.5 |
| SNPM01903 | LG1 | 29.5 |
| SNPM00858 | LG1 | 29.5 |
| SNPM04269 | LG1 | 29.8 |
| SNPM03929 | LG1 | 37.9 |
| SNPM03795 | LG1 | 37.9 |
| SNPM01495 | LG1 | 41.9 |
| sMo00092 | LG1 | 42.3 |
| SNPM01700 | LG1 | 42.3 |
| SNPM02866 | LG1 | 44.5 |
| SNPM04224 | LG1 | 45.9 |
| SNPM00277 | LG1 | 47.3 |
| SNPM02980 | LG1 | 47.3 |
| SNPM00277b | LG1 | 47.3 |
| SNPM00206 | LG1 | 47.3 |
| SNPM02981 | LG1 | 47.3 |
| SNPM03197 | LG1 | 51.0 |
| SNPM03482 | LG1 | 52.4 |
| SNPM01226 | LG1 | 53.9 |
| SNPM04771 | LG1 | 53.9 |
| SNPM03589 | LG1 | 57.7 |
| SNPM04651 | LG1 | 57.7 |
| SNPM03588 | LG1 | 57.7 |
| SNPM02328 | LG1 | 57.7 |
| sMo00071A | LG1 | 58.5 |
| SNPM02097 | LG1 | 58.5 |
| SNPM04728 | LG1 | 59.3 |
| SNPM00798 | LG1 | 59.3 |
| SNPM02437 | LG1 | 60.1 |
| Hght10_oSSR | LG1 | 60.1 |
| SNPM02031 | LG1 | 60.1 |
| SNPM00494 | LG1 | 60.1 |
| SNPM04246 | LG1 | 60.1 |
| SNPM02068 | LG1 | 60.1 |
| KA3_oSSR | LG1 | 60.1 |
| SNPM00138 | LG1 | 60.1 |
| SNPM02055 | LG1 | 60.4 |
| SNPM00599 | LG1 | 60.6 |
| SNPM03129 | LG1 | 60.6 |
| SNPM03128 | LG1 | 60.6 |
| KA1_oSSR | LG1 | 60.6 |
| SNPM04079 | LG1 | 60.6 |
| SNPM00894 | LG1 | 60.6 |
| SNPM04525 | LG1 | 60.6 |
| SNPM02077 | LG1 | 63.9 |
| SNPM01442 | LG1 | 63.9 |
| SNPM03343 | LG1 | 63.9 |
| SNPM04936 | LG1 | 63.9 |
| SNPM00480 | LG1 | 67.3 |
| SNPM02896 | LG1 | 67.3 |
| SNPM04544 | LG1 | 69.6 |
| SNPM01455 | LG1 | 69.6 |
| sMo00188 | LG1 | 72.0 |
| SNPM03299 | LG1 | 74.4 |
| SNPM03831 | LG1 | 74.4 |
| SNPM02870 | LG1 | 74.4 |
| SNPM00301 | LG1 | 75.6 |
| SNPM01283 | LG1 | 76.6 |
| SNPM00965 | LG1 | 76.8 |
| SNPM02337 | LG1 | 76.8 |
| SNPM04876 | LG1 | 77.2 |
| SNPM02883 | LG1 | 77.2 |
| SNPM02506 | LG1 | 77.7 |
| SNPM04202 | LG1 | 77.7 |
| SNPM03064 | LG1 | 77.7 |
| sMo00151 | LG1 | 77.8 |
| SNPM00735 | LG1 | 78.0 |
| SNPM02786 | LG1 | 78.0 |
| SNPM00232 | LG1 | 78.3 |
| SNPM02830 | LG1 | 78.3 |
| SNPM02249 | LG1 | 78.3 |
| SNPM01335 | LG1 | 79.7 |
| SNPM00207 | LG1 | 81.0 |
| SNPM00294 | LG1 | 81.0 |
| SNPM03716 | LG1 | 81.0 |
| SNPM04280 | LG1 | 81.0 |
| SNPM00336 | LG1 | 86.5 |
| SNPM00245 | LG1 | 86.5 |
| SNPM01854 | LG1 | 88.5 |
| SNPM02519 | LG1 | 88.5 |
| sPSc00488b | LG1 | 91.8 |
| SNPM04772 | LG1 | 92.6 |
| sPSc00481 | LG1 | 94.7 |
| SNPM04797 | LG1 | 95.2 |
| sPSc00490 | LG1 | 95.7 |
| SNPM01252 | LG1 | 98.3 |
| SNPM00665 | LG1 | 101.4 |
| SNPM01803 | LG1 | 101.4 |
| SNPM01789 | LG1 | 101.4 |
| SNPM00570 | LG1 | 101.4 |
| SNPM02063 | LG1 | 102.4 |
| SNPM00531 | LG1 | 105.6 |
| SNPM00591 | LG1 | 110.7 |
| sPSc00282 | LG1 | 111.0 |
| sPSc00306 | LG1 | 112.8 |
| SNPM01040 | LG1 | 113.2 |
| SNPE00441 | LG1 | 114.7 |
| SNPM04130 | LG1 | 122.2 |
| sMg00197 | LG1 | 123.9 |
| SNPM01602 | LG1 | 124.9 |
| sMo00292B | LG1 | 124.9 |
| SNPM01136 | LG1 | 124.9 |
| SNPM03869 | LG1 | 124.9 |
| SNPM01508 | LG1 | 125.5 |
| SNPM00262 | LG1 | 126.2 |
| SNPM00516 | LG1 | 126.2 |
| SNPM02158 | LG1 | 126.2 |
| SNPM00401 | LG1 | 126.2 |
| SNPM00286 | LG1 | 126.2 |
| SNPM01535 | LG1 | 127.6 |
| SNPM02305 | LG1 | 131.8 |
| SNPM04484 | LG1 | 131.8 |
| SNPM01452 | LG1 | 132.5 |
| SNPM01034 | LG1 | 133.2 |
| SNPM00796 | LG1 | 133.4 |
| SNPM04501 | LG1 | 133.5 |
| SNPM02759 | LG1 | 133.7 |
| SNPM03159 | LG1 | 133.9 |
| SNPM00450 | LG1 | 136.0 |
| SNPM03786 | LG1 | 138.8 |
| SNPM00078 | LG2 | 0.0 |
| SNPM03771 | LG2 | 1.4 |
| SNPM00704 | LG2 | 1.4 |
| SNPM03865 | LG2 | 1.4 |
| SNPM01104 | LG2 | 1.4 |
| SNPM02938 | LG2 | 2.6 |
| SNPM02085 | LG2 | 2.6 |
| SNPM04486 | LG2 | 4.7 |
| SNPM00622 | LG2 | 7.8 |
| SNPM03844 | LG2 | 7.8 |
| SNPM03828 | LG2 | 7.9 |
| SNPM03862 | LG2 | 8.0 |
| SNPM01522 | LG2 | 8.1 |
| SNPM01149 | LG2 | 8.1 |
| SNPM00122 | LG2 | 8.1 |
| SNPM02370 | LG2 | 15.1 |
| SNPM00260 | LG2 | 15.1 |
| SNPM04338 | LG2 | 15.1 |
| SNPM03099 | LG2 | 15.1 |
| SNPM00799 | LG2 | 15.1 |
| SNPM05021 | LG2 | 19.0 |
| SNPM00491 | LG2 | 22.8 |
| SNPM03845 | LG2 | 26.1 |
| SNPM02665 | LG2 | 27.2 |
| SNPM04909 | LG2 | 27.2 |
| SNPM00325 | LG2 | 27.2 |
| SNPM02130 | LG2 | 27.2 |
| SNPM02666 | LG2 | 27.3 |
| SNPM03435 | LG2 | 27.4 |
| SNPE00427 | LG2 | 27.8 |
| SNPM03297 | LG2 | 28.9 |
| SNPM04244 | LG2 | 30.6 |
| SNPM04243 | LG2 | 30.6 |
| SNPM04511 | LG2 | 35.9 |
| SNPM04410 | LG2 | 37.4 |
| SNPM02350 | LG2 | 37.4 |
| SNPM02736 | LG2 | 38.6 |
| SNPM00962 | LG2 | 39.5 |
| SNPM01889 | LG2 | 40.4 |
| SNPM01052 | LG2 | 40.4 |
| SNPM04068 | LG2 | 41.8 |
| SNPM01969 | LG2 | 42.4 |
| SNPM01906 | LG2 | 46.1 |
| SNPM00425 | LG2 | 46.8 |
| SNPM02334 | LG2 | 47.4 |
| sMo00222 | LG2 | 49.5 |
| SNPM02641 | LG2 | 49.5 |
| SNPM04480 | LG2 | 49.5 |
| sMg00235 | LG2 | 49.5 |
| SNPM02701 | LG2 | 49.5 |
| SNPM04235 | LG2 | 52.2 |
| SNPM00177 | LG2 | 52.2 |
| SNPM04542 | LG2 | 57.3 |
| SNPM02818 | LG2 | 57.8 |
| SNPM02750 | LG2 | 57.8 |
| SNPM01453 | LG2 | 57.8 |
| SNPM00493 | LG2 | 57.8 |
| SNPM02214 | LG2 | 59.0 |
| SNPM01911 | LG2 | 59.0 |
| SNPM02314 | LG2 | 59.4 |
| SNPM02213 | LG2 | 59.5 |
| SNPM00288 | LG2 | 62.7 |
| SNPM02740 | LG2 | 62.7 |
| SNPM02895 | LG2 | 62.7 |
| sMg00192 | LG2 | 65.1 |
| sMo00126 | LG2 | 68.4 |
| sMo00020 | LG2 | 69.0 |
| SNPM04355 | LG2 | 69.0 |
| SNPM00476b | LG2 | 69.5 |
| SNPM02693 | LG2 | 70.3 |
| SNPM01341 | LG2 | 71.3 |
| SNPM01089 | LG2 | 74.9 |
| SNPE00415 | LG2 | 74.9 |
| SNPE00416 | LG2 | 74.9 |
| SNPM00402 | LG2 | 74.9 |
| SNPM02298 | LG2 | 74.9 |
| SNPM01392 | LG2 | 77.8 |
| sEg00072 | LG2 | 77.8 |
| SNPM04869 | LG2 | 77.8 |
| SNPM02111 | LG2 | 83.6 |
| SNPM03170 | LG2 | 83.7 |
| SNPM04812 | LG2 | 86.7 |
| SNPM03231 | LG2 | 88.3 |
| SNPM02594 | LG2 | 88.3 |
| SNPM00037 | LG2 | 90.7 |
| SNPM01439 | LG2 | 91.9 |
| SNPM03846 | LG2 | 93.2 |
| SNPM00195 | LG2 | 95.6 |
| SNPM02689 | LG2 | 95.6 |
| SNPM00540 | LG2 | 96.9 |
| SNPM02941 | LG2 | 97.5 |
| SNPM04109 | LG2 | 98.2 |
| SNPM04275 | LG2 | 98.2 |
| SNPM04619 | LG2 | 98.2 |
| SNPM04762 | LG2 | 101.1 |
| SNPM04028 | LG2 | 102.3 |
| SNPM02390 | LG2 | 102.3 |
| SNPM03595 | LG2 | 102.4 |
| SNPM01194 | LG2 | 102.4 |
| SNPM00988 | LG2 | 106.6 |
| SNPM00746 | LG2 | 106.6 |
| SNPM00987 | LG2 | 106.6 |
| SNPM02999 | LG2 | 110.8 |
| SNPM01138 | LG2 | 110.8 |
| SNPM02729 | LG2 | 113.6 |
| SNPM00437 | LG2 | 113.6 |
| SNPM03680 | LG2 | 113.6 |
| sMg00011 | LG3 | 0.0 |
| SNPM02349 | LG3 | 4.4 |
| SNPM02639 | LG3 | 4.7 |
| SNPM00748 | LG3 | 4.7 |
| SNPM02316 | LG3 | 4.7 |
| SNPM04971 | LG3 | 4.7 |
| SNPM03909 | LG3 | 4.7 |
| SNPM01461 | LG3 | 5.7 |
| SNPM01423 | LG3 | 7.6 |
| SNPM00550 | LG3 | 7.9 |
| SNPM00362 | LG3 | 12.4 |
| SNPM02983 | LG3 | 12.4 |
| SNPM01899 | LG3 | 12.5 |
| SNPM01211 | LG3 | 12.8 |
| SNPM04197 | LG3 | 15.8 |
| SNPM04340 | LG3 | 15.8 |
| SNPM02899 | LG3 | 19.9 |
| SNPM01030 | LG3 | 22.0 |
| SNPM02137 | LG3 | 23.0 |
| SNPM00823 | LG3 | 29.3 |
| SNPM05073 | LG3 | 29.3 |
| SNPM02046 | LG3 | 29.3 |
| SNPM03647 | LG3 | 29.7 |
| SNPM02590 | LG3 | 30.2 |
| SNPM02955 | LG3 | 30.2 |
| SNPM00560 | LG3 | 30.2 |
| SNPM01170 | LG3 | 30.2 |
| SNPM02990 | LG3 | 30.7 |
| SNPM02215 | LG3 | 36.2 |
| SNPM00561 | LG3 | 37.2 |
| SNPM02538 | LG3 | 39.3 |
| SNPM03711 | LG3 | 39.8 |
| sMo00180 | LG3 | 40.7 |
| sPSc00546 | LG3 | 41.7 |
| SNPM00874 | LG3 | 41.7 |
| SNPM01446 | LG3 | 44.5 |
| SNPM04146 | LG3 | 44.5 |
| SNPM04230 | LG3 | 46.5 |
| SNPM00536 | LG3 | 46.5 |
| SNPM00541 | LG3 | 46.5 |
| SNPM00261 | LG3 | 46.5 |
| SNPM03122 | LG3 | 49.8 |
| SNPM02323 | LG3 | 51.7 |
| SNPM00118 | LG3 | 52.3 |
| SNPM00462 | LG3 | 52.3 |
| SNPM04198 | LG3 | 54.8 |
| SNPM04474 | LG3 | 54.8 |
| SNPM02100A | LG3 | 55.2 |
| SNPM01954 | LG3 | 55.5 |
| SNPM01739 | LG3 | 56.8 |
| SNPM03949A | LG3 | 57.5 |
| SNPM00731 | LG3 | 57.5 |
| SNPM01767 | LG3 | 57.5 |
| SNPM02723 | LG3 | 57.5 |
| SNPM01114 | LG4 | 0.0 |
| SNPM02910 | LG4 | 0.4 |
| sPSc00550 | LG4 | 0.4 |
| SNPM00172 | LG4 | 0.4 |
| SNPM02501 | LG4 | 0.9 |
| SNPM00297 | LG4 | 1.9 |
| SNPM02402 | LG4 | 1.9 |
| SNPM00249 | LG4 | 3.4 |
| SNPM04449 | LG4 | 3.4 |
| SNPM00348 | LG4 | 3.4 |
| SNPM00203 | LG4 | 3.4 |
| SNPM00563 | LG4 | 4.3 |
| SNPM00151 | LG4 | 4.3 |
| SNPM00971 | LG4 | 4.3 |
| SNPM00692 | LG4 | 8.1 |
| SNPM03201 | LG4 | 11.3 |
| SNPM01175 | LG4 | 11.3 |
| SNPM02238 | LG4 | 11.3 |
| SNPM03096 | LG4 | 11.3 |
| SNPM02797 | LG4 | 11.3 |
| SNPM03338 | LG4 | 14.9 |
| SNPM04121 | LG4 | 14.9 |
| SNPM01908 | LG4 | 14.9 |
| SNPM00751 | LG4 | 15.6 |
| SNPM02970 | LG4 | 16.2 |
| SNPM04696 | LG4 | 17.4 |
| SNPM02140 | LG4 | 17.4 |
| SNPM00699 | LG4 | 17.4 |
| SNPM04441 | LG4 | 20.8 |
| SNPM01703 | LG4 | 20.8 |
| SNPM01296 | LG4 | 20.8 |
| SNPM00357 | LG4 | 20.8 |
| SNPM00898 | LG4 | 22.4 |
| SNPM00555 | LG4 | 24.4 |
| SNPM00227 | LG4 | 26.9 |
| SNPM01026 | LG4 | 30.0 |
| SNPM03898 | LG4 | 30.0 |
| SNPM00075 | LG4 | 31.8 |
| SNPM01989 | LG4 | 31.8 |
| SNPM03728 | LG4 | 32.2 |
| SNPM01330 | LG4 | 32.2 |
| SNPM01910 | LG4 | 38.8 |
| SNPM02110 | LG4 | 39.0 |
| SNPM03640 | LG4 | 39.2 |
| SNPM00669 | LG4 | 39.2 |
| sEg00135 | LG4 | 40.6 |
| sMg00191 | LG4 | 42.9 |
| sMg00209 | LG4 | 42.9 |
| SNPM04796 | LG4 | 48.5 |
| SNPM01126 | LG4 | 48.9 |
| SNPM02905 | LG4 | 50.5 |
| SNPM03154 | LG4 | 52.1 |
| sMg00027 | LG4 | 52.3 |
| SNPM03433 | LG4 | 52.5 |
| sEg00069 | LG4 | 52.5 |
| SNPM02081 | LG4 | 52.8 |
| SNPM01760 | LG4 | 53.0 |
| SNPM03992 | LG4 | 53.0 |
| SNPM03697 | LG4 | 53.4 |
| SNPM01023 | LG4 | 54.6 |
| SNPM01359 | LG4 | 54.6 |
| SNPM05078 | LG4 | 57.0 |
| SNPM03337 | LG4 | 57.0 |
| SNPM00185 | LG4 | 57.0 |
| SNPM00682 | LG4 | 57.0 |
| SNPM03163 | LG4 | 58.4 |
| SNPM03164 | LG4 | 58.4 |
| SNPM01555 | LG4 | 61.1 |
| SNPM01128 | LG4 | 62.9 |
| SNPM02397 | LG4 | 64.7 |
| SNPM00365 | LG4 | 66.0 |
| SNPM00216 | LG4 | 66.0 |
| SNPM04680 | LG4 | 66.0 |
| SNPM00614 | LG4 | 66.0 |
| SNPM04118 | LG4 | 66.0 |
| SNPM04385 | LG4 | 68.2 |
| SNPM04705 | LG4 | 71.3 |
| SNPM05062 | LG4 | 71.3 |
| sMg00041 | LG4 | 74.6 |
| SNPM04569 | LG4 | 75.7 |
| SNPM00790 | LG4 | 75.7 |
| SNPM00342 | LG4 | 77.2 |
| SNPM03470 | LG4 | 77.2 |
| SNPM03900 | LG4 | 85.8 |
| SNPM04211A | LG4 | 85.8 |
| SNPE00405 | LG4 | 85.8 |
| SNPM04580 | LG4 | 85.8 |
| SNPM00186 | LG4 | 88.7 |
| SNPM00653 | LG4 | 90.9 |
| SNPM00375 | LG4 | 92.2 |
| SNPM03523 | LG4 | 92.2 |
| SNPM04395 | LG4 | 93.9 |
| SNPM04136 | LG4 | 93.9 |
| SNPM02681 | LG4 | 93.9 |
| SNPM00359 | LG4 | 93.9 |
| SNPM03326 | LG4 | 95.8 |
| SNPM02577 | LG4 | 96.7 |
| SNPM02193 | LG4 | 98.5 |
| SNPM02023A | LG4 | 98.5 |
| SNPM03746 | LG4 | 98.5 |
| SNPE00436 | LG4 | 98.5 |
| SNPM02783 | LG4 | 98.5 |
| SNPM00918 | LG4 | 100.8 |
| SNPM02190 | LG4 | 102.2 |
| SNPM02065 | LG4 | 102.7 |
| SNPM02604 | LG4 | 102.7 |
| SNPM02064 | LG4 | 102.7 |
| SNPM00496 | LG4 | 107.8 |
| SNPM01229 | LG4 | 111.6 |
| sEg00094 | LG4 | 112.1 |
| SNPM01258 | LG4 | 112.1 |
| SNPM00957 | LG4 | 115.2 |
| SNPM02461 | LG4 | 115.2 |
| SNPM00958 | LG4 | 115.2 |
| SNPM02460 | LG4 | 115.2 |
| SNPM01193 | LG4 | 116.3 |
| SNPM04731 | LG4 | 116.9 |
| SNPM03958 | LG4 | 116.9 |
| SNPM03087 | LG4 | 117.4 |
| SNPM03631 | LG4 | 117.4 |
| SNPM00785 | LG4 | 118.0 |
| SNPM00464 | LG4 | 118.0 |
| SNPM03573 | LG4 | 120.5 |
| SNPM00427 | LG4 | 123.0 |
| SNPM00275 | LG4 | 124.3 |
| SNPM00929 | LG4 | 124.3 |
| SNPM00568 | LG4 | 124.4 |
| SNPM02852 | LG4 | 125.5 |
| SNPM03990 | LG4 | 129.7 |
| SNPM00772 | LG4 | 130.7 |
| SNPM03116 | LG4 | 132.8 |
| SNPM04819 | LG4 | 132.8 |
| SNPE00432 | LG4 | 132.8 |
| SNPM04724 | LG4 | 136.3 |
| SNPM00183 | LG4 | 137.3 |
| sMo00012 | LG4 | 139.5 |
| SNPM03463 | LG4 | 139.5 |
| SNPM03753 | LG4 | 139.5 |
| SNPM03806 | LG4 | 141.9 |
| SNPM00059b | LG4 | 142.6 |
| SNPM02155 | LG4 | 143.7 |
| SNPM00163 | LG4 | 143.7 |
| SNPM03867 | LG4 | 143.7 |
| SNPM04745 | LG4 | 143.7 |
| sEg00166 | LG4 | 144.0 |
| SNPM02278 | LG4 | 144.7 |
| SNPM01355 | LG4 | 145.6 |
| SNPM01362 | LG4 | 145.6 |
| SNPM00296 | LG4 | 146.2 |
| SNPM04986 | LG4 | 146.2 |
| SNPM00059 | LG4 | 146.2 |
| SNPM02403 | LG4 | 148.9 |
| sMg00155 | LG4 | 148.9 |
| SNPM02227 | LG4 | 150.4 |
| SNPM02362 | LG4 | 150.4 |
| SNPM04163 | LG4 | 150.7 |
| SNPM01979 | LG4 | 152.3 |
| SNPM02652 | LG4 | 152.3 |
| SNPM02991 | LG4 | 155.5 |
| SNPM02255 | LG4 | 157.5 |
| SNPM02160 | LG4 | 158.7 |
| SNPM04581 | LG4 | 158.7 |
| SNPM02745 | LG4 | 158.7 |
| SNPM00255 | LG4 | 159.3 |
| SNPM00908 | LG4 | 159.3 |
| SNPM03325 | LG4 | 159.9 |
| SNPM00121 | LG4 | 162.3 |
| SNPM01876 | LG4 | 164.2 |
| sMg00230 | LG4 | 164.8 |
| SNPM00341 | LG4 | 168.0 |
| SNPM00675 | LG4 | 168.0 |
| SNPM02507 | LG4 | 169.2 |
| SNPM00708 | LG4 | 169.2 |
| SNPM01134 | LG4 | 170.3 |
| SNPM02564 | LG4 | 170.3 |
| SNPM01231 | LG4 | 173.9 |
| sMo00138 | LG4 | 176.8 |
| SNPM04470 | LG4 | 180.4 |
| SNPM05008 | LG4 | 181.0 |
| SNPM01485 | LG4 | 181.3 |
| SNPM00729 | LG4 | 181.8 |
| SNPM00628 | LG4 | 181.8 |
| sEg00146 | LG4 | 183.6 |
| SNPM02187 | LG4 | 184.6 |
| SNPM00808 | LG4 | 186.0 |
| SNPM04461 | LG4 | 187.4 |
| SNPM04947 | LG4 | 188.1 |
| SNPM01759 | LG4 | 188.1 |
| SNPM00363 | LG4 | 188.1 |
| SNPM04992 | LG4 | 188.1 |
| SNPM00258 | LG4 | 188.1 |
| SNPM01342 | LG4 | 190.5 |
| SNPM04352 | LG4 | 190.8 |
| SNPM03093 | LG4 | 191.8 |
| SNPM00233 | LG4 | 191.8 |
| SNPM03375 | LG4 | 192.5 |
| SNPM03981 | LG4 | 193.1 |
| SNPM02535 | LG4 | 193.1 |
| SNPM04681 | LG4 | 193.1 |
| SNPM00672 | LG4 | 193.1 |
| SNPM04038 | LG4 | 195.4 |
| SNPM03655 | LG4 | 195.4 |
| SNPM03489 | LG5 | 0.0 |
| SNPM03308 | LG5 | 2.1 |
| SNPM00975 | LG5 | 42.5 |
| SNPM04445 | LG5 | 42.5 |
| SNPM03309 | LG5 | 43.2 |
| SNPM04225 | LG5 | 48.1 |
| SNPM03432 | LG5 | 55.9 |
| SNPM00705 | LG5 | 55.9 |
| SNPM02084 | LG5 | 56.7 |
| SNPM00409 | LG5 | 56.7 |
| SNPM03152 | LG5 | 56.7 |
| SNPM02388 | LG5 | 60.8 |
| SNPM02221 | LG5 | 60.8 |
| SNPM04889 | LG5 | 63.3 |
| SNPM04758 | LG5 | 63.3 |
| SNPM04155 | LG5 | 63.7 |
| SNPM00868 | LG5 | 63.7 |
| SNPM03235 | LG5 | 63.7 |
| sMo00130 | LG5 | 72.0 |
| SNPM00601 | LG5 | 74.6 |
| SNPM03055 | LG5 | 74.6 |
| SNPM01689 | LG5 | 74.6 |
| SNPM00253 | LG5 | 74.6 |
| SNPM00625 | LG5 | 79.2 |
| SNPM00267 | LG5 | 79.2 |
| SNPM01049 | LG5 | 79.2 |
| SNPM02826 | LG5 | 79.8 |
| SNPM02518 | LG5 | 86.5 |
| sEg00085A | LG5 | 86.5 |
| sEg00085B | LG5 | 86.5 |
| sMg00025 | LG5 | 90.3 |
| sPSc00537 | LG5 | 90.3 |
| sEg00119 | LG5 | 91.9 |
| sEg00059 | LG5 | 93.0 |
| sEg00007 | LG5 | 93.0 |
| sEg00054 | LG5 | 94.2 |
| sEg00058 | LG5 | 95.4 |
| SNPM04868 | LG5 | 96.6 |
| SNPM04490 | LG5 | 96.6 |
| SNPM03086 | LG5 | 96.6 |
| SNPM04311 | LG5 | 99.7 |
| SNPM02264 | LG5 | 99.7 |
| SNPM00270 | LG5 | 99.9 |
| SNPM01400 | LG5 | 100.2 |
| sMo00040 | LG6 | 0.0 |
| SNPM01769 | LG6 | 2.1 |
| SNPM04978 | LG6 | 2.1 |
| SNPM00422 | LG6 | 2.1 |
| SNPM04399 | LG6 | 2.1 |
| SNPM03138 | LG6 | 7.1 |
| SNPM04753 | LG6 | 7.1 |
| SNPM02635 | LG6 | 10.9 |
| SNPM02634 | LG6 | 10.9 |
| SNPM02669 | LG6 | 11.9 |
| SNPM03703 | LG6 | 13.1 |
| SNPM01063 | LG6 | 16.9 |
| SNPM00500 | LG6 | 22.2 |
| SNPM02863 | LG6 | 22.2 |
| SNPM00218 | LG6 | 25.0 |
| SNPM02011 | LG6 | 25.0 |
| SNPM05061 | LG6 | 29.2 |
| SNPM04366 | LG6 | 29.2 |
| SNPM03209 | LG6 | 29.2 |
| SNPM00198 | LG6 | 29.2 |
| SNPM04549 | LG6 | 29.2 |
| SNPM03798 | LG6 | 31.2 |
| sEg00110 | LG6 | 35.2 |
| SNPM02780 | LG6 | 37.2 |
| SNPM03192 | LG6 | 38.5 |
| SNPM02654 | LG6 | 45.0 |
| SNPM04414 | LG6 | 46.0 |
| SNPM00259 | LG6 | 48.7 |
| SNPM00534 | LG6 | 48.7 |
| SNPM05023 | LG6 | 50.5 |
| SNPM04085 | LG6 | 54.2 |
| SNPM03591 | LG6 | 54.2 |
| SNPM03488 | LG6 | 57.8 |
| SNPM00679 | LG6 | 58.7 |
| SNPM01217 | LG6 | 58.7 |
| SNPM01218 | LG6 | 58.7 |
| SNPM01948 | LG6 | 60.1 |
| SNPM00209 | LG6 | 60.1 |
| SNPM01879 | LG6 | 62.3 |
| SNPM03383 | LG6 | 64.6 |
| SNPM01143 | LG6 | 69.3 |
| SNPM01615 | LG6 | 69.5 |
| SNPM00007 | LG6 | 69.7 |
| SNPM02748A | LG6 | 69.7 |
| SNPM04810b | LG6 | 71.0 |
| SNPM03599 | LG6 | 71.1 |
| SNPM03600 | LG6 | 71.1 |
| mEgCIR3543 | LG6 | 71.1 |
| SNPM03683 | LG6 | 72.5 |
| SNPM00631 | LG6 | 73.7 |
| sEg00154 | LG6 | 73.7 |
| SNPM00603 | LG6 | 77.0 |
| SNPM05051 | LG6 | 77.0 |
| SNPM01722 | LG6 | 80.7 |
| SNPM02320 | LG6 | 80.7 |
| SNPM01863 | LG6 | 80.7 |
| sMg00071 | LG6 | 85.1 |
| SNPM04029 | LG6 | 88.0 |
| SNPM02659 | LG6 | 88.7 |
| sMg00055 | LG6 | 90.2 |
| SNPM02710 | LG6 | 91.6 |
| SNPM03414 | LG6 | 91.6 |
| SNPM01440 | LG6 | 92.3 |
| SNPM03403 | LG6 | 92.3 |
| SNPM00314 | LG6 | 93.2 |
| SNPM02417 | LG6 | 93.6 |
| SNPM02387 | LG6 | 93.6 |
| SNPM01093 | LG6 | 93.6 |
| SNPM00522 | LG6 | 93.6 |
| SNPM01584 | LG6 | 93.6 |
| SNPM03823 | LG6 | 93.6 |
| SNPM04856 | LG6 | 93.6 |
| SNPM00300 | LG6 | 95.0 |
| SNPM03904 | LG6 | 95.0 |
| SNPM03903 | LG6 | 95.0 |
| SNPM00877 | LG6 | 95.0 |
| SNPM01286 | LG6 | 95.9 |
| SNPM04857A | LG6 | 95.9 |
| SNPM01285 | LG6 | 95.9 |
| SNPM03978 | LG6 | 95.9 |
| sMg00205 | LG6 | 100.0 |
| SNPM02163 | LG6 | 103.1 |
| SNPM00843 | LG6 | 103.1 |
| SNPM01354 | LG6 | 103.1 |
| SNPM01479 | LG6 | 103.7 |
| SNPM04723 | LG6 | 103.7 |
| SNPM03081 | LG6 | 103.7 |
| SNPM00968 | LG6 | 104.2 |
| SNPM04921 | LG6 | 104.2 |
| SNPM02418 | LG6 | 105.4 |
| sMo00212B | LG6 | 105.4 |
| SNPM04261 | LG6 | 105.4 |
| SNPM01638 | LG6 | 105.4 |
| sMo00212A | LG6 | 106.2 |
| SNPM02494 | LG6 | 107.1 |
| sMo00081 | LG6 | 107.1 |
| SNPM00545 | LG6 | 107.9 |
| SNPM03570 | LG6 | 107.9 |
| SNPM02182 | LG6 | 108.2 |
| SNPM00575 | LG6 | 108.2 |
| SNPM00969 | LG6 | 108.6 |
| SNPM02735 | LG6 | 109.3 |
| SNPM01927 | LG6 | 110.2 |
| SNPM00229 | LG6 | 110.2 |
| sMo00197 | LG6 | 110.2 |
| sMo00156 | LG6 | 110.2 |
| SNPM00208 | LG6 | 110.2 |
| SNPM04545 | LG6 | 110.2 |
| SNPM00848 | LG6 | 110.2 |
| SNPM04394 | LG6 | 113.0 |
| SNPM00199 | LG6 | 113.0 |
| SNPM03689 | LG6 | 113.0 |
| sMo00132 | LG6 | 114.3 |
| SNPM04509 | LG6 | 115.6 |
| SNPM00215 | LG6 | 115.6 |
| SNPM04686 | LG6 | 115.6 |
| sMg00219B | LG6 | 115.6 |
| SNPM00663 | LG6 | 115.6 |
| SNPM01050 | LG6 | 115.6 |
| SNPM00390 | LG6 | 115.6 |
| SNPM01715 | LG6 | 116.9 |
| SNPM05057 | LG6 | 116.9 |
| SNPM04770 | LG6 | 118.3 |
| SNPM00504 | LG6 | 118.9 |
| SNPM05010 | LG6 | 119.5 |
| SNPM04170 | LG6 | 120.2 |
| SNPM03186 | LG6 | 120.9 |
| SNPM04356 | LG6 | 120.9 |
| SNPM04958 | LG6 | 121.3 |
| SNPM02016 | LG6 | 121.5 |
| SNPM04080 | LG6 | 121.7 |
| sMg00051 | LG6 | 121.9 |
| SNPM01886 | LG6 | 121.9 |
| SNPM03304 | LG6 | 121.9 |
| SNPM03198 | LG6 | 122.6 |
| SNPM04967 | LG7 | 0.0 |
| SNPM01322 | LG7 | 0.7 |
| SNPM03724b | LG7 | 0.7 |
| SNPM04990 | LG7 | 10.3 |
| SNPM02913 | LG7 | 10.3 |
| SNPM00825 | LG7 | 10.3 |
| SNPM02052 | LG7 | 10.8 |
| SNPM03344 | LG7 | 10.8 |
| SNPM02296b | LG7 | 10.8 |
| SNPM02650 | LG7 | 10.8 |
| SNPM01950 | LG7 | 10.8 |
| SNPM01701 | LG7 | 10.8 |
| SNPM02651 | LG7 | 10.8 |
| SNPM01845 | LG7 | 10.8 |
| SNPM00626 | LG7 | 10.8 |
| SNPM00779 | LG7 | 11.1 |
| SNPM03807 | LG7 | 11.1 |
| SNPM03759 | LG7 | 11.1 |
| SNPM04991 | LG7 | 11.1 |
| SNPM03427 | LG7 | 11.1 |
| SNPM00838 | LG7 | 13.1 |
| SNPM03582 | LG7 | 13.1 |
| sMo00246 | LG7 | 15.3 |
| SNPM02269 | LG7 | 16.8 |
| SNPM00371 | LG7 | 16.8 |
| SNPM00099 | LG7 | 16.8 |
| sMg00234 | LG7 | 19.4 |
| SNPM01519 | LG7 | 21.5 |
| SNPM03008 | LG7 | 21.5 |
| SNPM01518 | LG7 | 21.5 |
| SNPM00145 | LG7 | 21.5 |
| SNPM00247 | LG7 | 21.9 |
| SNPM03866 | LG7 | 21.9 |
| SNPM03560 | LG7 | 23.0 |
| SNPM04378 | LG7 | 23.0 |
| SNPM00535 | LG7 | 26.4 |
| SNPM02196 | LG7 | 26.4 |
| SNPM03822 | LG7 | 26.4 |
| SNPM02385 | LG7 | 26.4 |
| SNPM02384 | LG7 | 26.4 |
| SNPM01798 | LG7 | 26.4 |
| SNPM00175 | LG7 | 29.0 |
| SNPM04582 | LG7 | 29.0 |
| SNPM01153 | LG7 | 29.0 |
| SNPM00830 | LG7 | 29.0 |
| SNPM00829 | LG7 | 29.0 |
| SNPM03454 | LG7 | 29.0 |
| SNPM04274 | LG7 | 30.5 |
| SNPM00109 | LG7 | 32.0 |
| SNPM04131b | LG7 | 32.0 |
| SNPM04057 | LG7 | 32.1 |
| SNPM01794 | LG7 | 32.4 |
| SNPM03050 | LG7 | 33.3 |
| SNPM02626 | LG7 | 33.3 |
| SNPM02625 | LG7 | 33.3 |
| SNPM00840 | LG7 | 34.0 |
| SNPM00564 | LG7 | 34.0 |
| SNPM01315 | LG7 | 34.6 |
| SNPM00919 | LG7 | 35.4 |
| SNPM02593 | LG7 | 35.4 |
| SNPM02894 | LG7 | 37.6 |
| SNPM01524 | LG7 | 38.3 |
| SNPM03810 | LG7 | 38.3 |
| SNPM02854 | LG7 | 38.3 |
| SNPM00831 | LG7 | 38.3 |
| SNPM04776 | LG7 | 38.3 |
| sMo00102 | LG7 | 38.3 |
| SNPM03182 | LG7 | 38.3 |
| SNPM01036 | LG7 | 38.3 |
| SNPM01220 | LG7 | 38.3 |
| SNPM02184 | LG7 | 38.3 |
| SNPM02719 | LG7 | 38.3 |
| SNPM04606 | LG7 | 38.3 |
| SNPM02629 | LG7 | 38.7 |
| SNPM00770 | LG7 | 39.1 |
| SNPM05069 | LG7 | 39.4 |
| SNPM03695 | LG7 | 45.9 |
| SNPM01501A | LG7 | 48.2 |
| SNPM04625 | LG7 | 48.2 |
| SNPM00179 | LG7 | 48.2 |
| SNPM04019 | LG7 | 48.2 |
| SNPM05079 | LG7 | 48.2 |
| sMg00096 | LG7 | 53.4 |
| SNPM00938 | LG7 | 56.5 |
| SNPM03931 | LG7 | 56.5 |
| SNPM05068 | LG7 | 56.5 |
| SNPM02477 | LG7 | 57.7 |
| mEgCIR3389 | LG7 | 58.5 |
| SNPM03466 | LG7 | 60.1 |
| sMo00260 | LG7 | 60.6 |
| SNPM00355 | LG7 | 61.4 |
| SNPM02549 | LG7 | 62.5 |
| SNPM01850 | LG7 | 62.5 |
| SNPM03516 | LG7 | 63.8 |
| SNPM01157 | LG7 | 65.1 |
| SNPM04479 | LG7 | 65.8 |
| SNPM00807 | LG7 | 65.8 |
| SNPM01275 | LG7 | 67.2 |
| SNPM03362 | LG7 | 67.2 |
| SNPM04638 | LG7 | 68.3 |
| SNPM00783 | LG7 | 69.3 |
| SNPM01603 | LG7 | 70.6 |
| SNPM02782 | LG7 | 70.6 |
| SNPM04377 | LG7 | 74.6 |
| SNPM03780 | LG7 | 74.6 |
| SNPM04802 | LG7 | 74.6 |
| SNPM01512 | LG7 | 74.6 |
| SNPM00547 | LG7 | 74.6 |
| SNPM01007 | LG7 | 76.3 |
| SNPM03066 | LG7 | 76.3 |
| SNPM03957 | LG7 | 77.1 |
| SNPM01666A | LG7 | 77.1 |
| SNPM01182 | LG7 | 78.2 |
| sMo00170 | LG7 | 78.2 |
| SNPM01025 | LG7 | 79.3 |
| SNPM04201 | LG7 | 79.9 |
| SNPM01678 | LG7 | 79.9 |
| SNPM04343 | LG7 | 80.9 |
| SNPM01616 | LG7 | 81.4 |
| SNPM04065 | LG7 | 81.9 |
| SNPM04996 | LG7 | 83.7 |
| SNPM01928 | LG7 | 83.7 |
| SNPM00742 | LG7 | 85.4 |
| SNPM04997 | LG7 | 85.4 |
| SNPM01567 | LG7 | 85.4 |
| SNPM01710 | LG7 | 85.4 |
| SNPM03868 | LG7 | 86.2 |
| SNPM03126 | LG7 | 87.0 |
| SNPM04325 | LG7 | 87.0 |
| SNPM04429 | LG7 | 87.0 |
| SNPM04184 | LG7 | 87.0 |
| SNPM03859b | LG7 | 87.0 |
| SNPM00180 | LG7 | 87.0 |
| SNPM00690 | LG7 | 87.2 |
| SNPM04324 | LG7 | 87.3 |
| SNPM00842 | LG7 | 87.5 |
| SNPM00061 | LG7 | 87.6 |
| SNPM02200 | LG7 | 87.7 |
| SNPM02561 | LG7 | 87.9 |
| SNPM01728 | LG7 | 88.1 |
| SNPM01668 | LG7 | 88.1 |
| SNPM04430 | LG7 | 88.1 |
| SNPM04183 | LG7 | 88.1 |
| SNPM02562 | LG7 | 88.1 |
| SNPM02680 | LG7 | 89.2 |
| SNPM04597 | LG7 | 89.6 |
| SNPM02343 | LG7 | 89.6 |
| SNPM01729 | LG7 | 89.8 |
| SNPM01981 | LG7 | 89.9 |
| SNPM04571 | LG7 | 89.9 |
| SNPM03494 | LG7 | 89.9 |
| SNPM03401 | LG7 | 90.6 |
| SNPM04553 | LG7 | 90.6 |
| SNPM04552 | LG7 | 90.6 |
| SNPM01683 | LG7 | 91.2 |
| SNPM01032 | LG7 | 91.2 |
| SNPM02698 | LG7 | 92.6 |
| SNPM03022 | LG7 | 93.9 |
| SNPM00302 | LG7 | 93.9 |
| SNPM01484 | LG7 | 93.9 |
| SNPM00318 | LG7 | 95.0 |
| SNPM04879 | LG7 | 95.0 |
| SNPM02960 | LG7 | 95.3 |
| SNPM01162 | LG7 | 95.9 |
| SNPM01982 | LG7 | 96.4 |
| SNPM03739 | LG7 | 96.4 |
| SNPM03738 | LG7 | 96.4 |
| SNPM00872 | LG7 | 97.3 |
| SNPM02375 | LG7 | 97.4 |
| sMo00209 | LG7 | 97.9 |
| sMo00121 | LG7 | 97.9 |
| SNPM03606 | LG7 | 101.5 |
| SNPM03714 | LG7 | 101.5 |
| SNPM01350 | LG7 | 104.1 |
| SNPM00976 | LG7 | 104.9 |
| SNPM01047 | LG7 | 104.9 |
| SNPM00119 | LG7 | 105.5 |
| SNPM02560 | LG7 | 105.5 |
| SNPM02614 | LG7 | 107.4 |
| SNPM03960 | LG7 | 108.5 |
| SNPM04928 | LG7 | 110.8 |
| SNPM00010 | LG7 | 111.9 |
| SNPM00391 | LG7 | 111.9 |
| SNPM01380 | LG7 | 111.9 |
| SNPM03284 | LG7 | 112.6 |
| SNPM03657 | LG7 | 112.6 |
| SNPM02473 | LG7 | 113.2 |
| SNPM03989 | LG7 | 113.2 |
| SNPM02105 | LG7 | 113.2 |
| SNPM00200 | LG8 | 0.0 |
| SNPM00368 | LG8 | 0.0 |
| SNPM04176 | LG8 | 1.8 |
| SNPM00977 | LG8 | 1.8 |
| SNPM02743 | LG8 | 4.2 |
| SNPM00884 | LG8 | 7.2 |
| SNPM03276 | LG8 | 7.2 |
| SNPM01129 | LG8 | 7.2 |
| SNPM00157 | LG8 | 8.0 |
| SNPM04508 | LG8 | 9.1 |
| sMg00227 | LG8 | 14.4 |
| SNPM00173 | LG8 | 16.1 |
| SNPM04954 | LG8 | 16.1 |
| SNPM01388 | LG8 | 16.1 |
| SNPM01012 | LG8 | 21.6 |
| SNPM04032 | LG8 | 21.6 |
| SNPM03685 | LG8 | 21.6 |
| SNPM01041 | LG8 | 22.1 |
| SNPM02596 | LG8 | 22.1 |
| SNPM00461 | LG8 | 22.3 |
| SNPM04284 | LG8 | 22.5 |
| SNPM00353 | LG8 | 22.5 |
| SNPM00155 | LG8 | 22.5 |
| SNPM00224 | LG8 | 25.9 |
| SNPM02408 | LG8 | 25.9 |
| SNPM00719 | LG8 | 32.7 |
| SNPM04620 | LG8 | 33.8 |
| SNPM03402 | LG8 | 34.7 |
| SNPM02989 | LG8 | 34.7 |
| SNPM01670 | LG8 | 38.9 |
| SNPM05029 | LG8 | 41.9 |
| SNPM01653 | LG8 | 42.8 |
| SNPM02646 | LG8 | 43.4 |
| SNPM02591 | LG8 | 43.4 |
| SNPM04690 | LG8 | 43.5 |
| SNPM03249 | LG8 | 44.0 |
| SNPM04333 | LG8 | 44.6 |
| sMg00236 | LG8 | 45.7 |
| SNPM04707 | LG8 | 45.7 |
| SNPM01613 | LG8 | 47.5 |
| SNPM04365 | LG8 | 47.5 |
| sMg00208 | LG8 | 51.9 |
| SNPM00765 | LG8 | 53.0 |
| SNPM02234 | LG8 | 54.7 |
| SNPM01646 | LG8 | 55.9 |
| SNPM00405 | LG8 | 55.9 |
| SNPM00013 | LG8 | 55.9 |
| SNPM04251 | LG8 | 55.9 |
| SNPM02347 | LG8 | 55.9 |
| SNPM02998 | LG8 | 56.8 |
| SNPM02090 | LG8 | 62.2 |
| SNPM02755 | LG8 | 63.8 |
| SNPM04204 | LG8 | 64.5 |
| SNPM04206 | LG8 | 65.2 |
| SNPM02400 | LG8 | 65.2 |
| SNPM04989 | LG8 | 65.2 |
| SNPM00364 | LG8 | 65.9 |
| SNPM00383 | LG8 | 66.7 |
| SNPM03287 | LG8 | 67.2 |
| SNPM02425 | LG8 | 67.6 |
| SNPM02411 | LG8 | 68.0 |
| SNPM01405 | LG8 | 70.1 |
| SNPM01237 | LG8 | 70.1 |
| SNPM00520 | LG8 | 70.1 |
| SNPM01250 | LG8 | 73.3 |
| SNPM04222 | LG8 | 73.3 |
| SNPM00777 | LG8 | 73.3 |
| SNPM03010 | LG8 | 73.3 |
| SNPM02867 | LG8 | 73.6 |
| SNPM02708 | LG8 | 74.0 |
| SNPM00572 | LG8 | 76.1 |
| SNPM01672 | LG8 | 76.1 |
| SNPM04941 | LG8 | 79.0 |
| SNPM04863 | LG8 | 79.0 |
| SNPM04576 | LG8 | 81.6 |
| SNPM03620 | LG8 | 81.6 |
| SNPM02252 | LG8 | 81.6 |
| SNPM00387 | LG8 | 81.6 |
| SNPM04021 | LG8 | 81.6 |
| SNPM02655 | LG8 | 83.4 |
| sMg00223 | LG8 | 84.9 |
| SNPM01582 | LG8 | 85.6 |
| SNPM02581 | LG8 | 85.6 |
| SNPM02857 | LG8 | 87.2 |
| SNPM04345 | LG8 | 88.9 |
| SNPM00366 | LG8 | 88.9 |
| SNPM00257 | LG8 | 90.7 |
| SNPM03772 | LG8 | 92.6 |
| SNPM02774 | LG8 | 92.6 |
| mEgCIR0778 | LG8 | 92.8 |
| SNPM00608 | LG8 | 93.0 |
| SNPM03319A | LG8 | 93.0 |
| SNPM04945 | LG8 | 93.8 |
| SNPM03563 | LG8 | 96.9 |
| SNPM00326 | LG8 | 96.9 |
| SNPM02438 | LG8 | 96.9 |
| SNPM02176 | LG8 | 96.9 |
| SNPM00066 | LG8 | 99.7 |
| SNPM02530 | LG8 | 103.6 |
| sMo00238 | LG8 | 103.6 |
| SNPM04083 | LG8 | 103.6 |
| SNPM03727 | LG8 | 103.6 |
| SNPM02926 | LG8 | 103.6 |
| mEgCIR0246 | LG8 | 105.7 |
| sEg00016 | LG8 | 107.8 |
| SNPM00745 | LG8 | 107.8 |
| mEgCIR0439 | LG8 | 108.7 |
| SNPM03725 | LG8 | 112.2 |
| SNPM00235 | LG8 | 112.2 |
| SNPM01183 | LG8 | 112.2 |
| SNPM00635 | LG8 | 112.2 |
| SNPM02268 | LG8 | 112.2 |
| SNPM00549 | LG8 | 113.8 |
| SNPM00743 | LG8 | 116.2 |
| SNPM00621 | LG8 | 116.2 |
| SNPM04806 | LG8 | 116.2 |
| SNPM00130 | LG8 | 116.2 |
| SNPM05014 | LG8 | 116.2 |
| sMo00106 | LG8 | 116.2 |
| SNPM03215 | LG8 | 116.2 |
| SNPM01649 | LG8 | 116.2 |
| SNPM04010 | LG8 | 118.9 |
| SNPM01804 | LG8 | 118.9 |
| mEgCIR0555 | LG8 | 119.0 |
| SNPM01737 | LG8 | 124.4 |
| SNPM00658 | LG8 | 125.6 |
| SNPM02576 | LG8 | 126.5 |
| SNPM01551 | LG8 | 127.5 |
| SNPM02359 | LG8 | 132.5 |
| SNPM00316 | LG8 | 133.2 |
| SNPM03091 | LG8 | 133.2 |
| SNPM01687 | LG8 | 133.2 |
| SNPM03539 | LG8 | 134.6 |
| SNPM03226 | LG8 | 135.9 |
| SNPM02004 | LG8 | 137.9 |
| SNPM00217 | LG8 | 137.9 |
| sEg00213 | LG8 | 139.9 |
| SNPM01968 | LG8 | 143.9 |
| SNPM04052 | LG8 | 143.9 |
| SNPM02709 | LG8 | 143.9 |
| sMg00196 | LG8 | 143.9 |
| SNPM00339 | LG8 | 143.9 |
| SNPM02574 | LG8 | 143.9 |
| SNPM01410 | LG8 | 143.9 |
| SNPM03491 | LG8 | 146.3 |
| SNPM01310 | LG8 | 146.7 |
| SNPM03659 | LG8 | 147.2 |
| SNPM03669 | LG8 | 147.2 |
| SNPM01818 | LG8 | 151.5 |
| SNPM00906 | LG8 | 152.4 |
| SNPM04780 | LG8 | 152.4 |
| SNPM04779 | LG8 | 152.4 |
| SNPM03875 | LG8 | 152.9 |
| SNPM03135 | LG8 | 152.9 |
| SNPM02265 | LG9 | 0.0 |
| SNPM00137 | LG9 | 1.3 |
| SNPM04847 | LG9 | 1.5 |
| SNPM01923 | LG9 | 1.8 |
| SNPM02367 | LG9 | 1.8 |
| SNPM03469 | LG9 | 3.1 |
| SNPM01993 | LG9 | 3.7 |
| SNPM03993 | LG9 | 4.2 |
| SNPM03858 | LG9 | 4.2 |
| SNPM00886 | LG9 | 4.2 |
| SNPM03860 | LG9 | 4.2 |
| SNPM02982 | LG9 | 4.6 |
| SNPM03033 | LG9 | 5.1 |
| SNPM03833 | LG9 | 7.0 |
| SNPM00221 | LG9 | 13.2 |
| SNPM02035 | LG9 | 13.2 |
| SNPM01235 | LG9 | 13.2 |
| SNPM01395 | LG9 | 13.2 |
| SNPM01394 | LG9 | 13.2 |
| SNPM01493 | LG9 | 17.3 |
| SNPM02706 | LG9 | 17.3 |
| SNPM01851 | LG9 | 24.8 |
| sMg00016B | LG9 | 26.6 |
| sMg00016A | LG9 | 26.9 |
| SNPM00841 | LG9 | 30.3 |
| SNPM02570 | LG9 | 33.0 |
| SNPM01473 | LG9 | 34.4 |
| SNPM00228 | LG9 | 34.4 |
| SNPM01044 | LG9 | 35.7 |
| SNPM00961 | LG9 | 38.4 |
| SNPM00818 | LG9 | 38.4 |
| SNPM03124 | LG9 | 43.8 |
| SNPM00312 | LG9 | 45.1 |
| SNPM03518 | LG9 | 45.1 |
| SNPM00553 | LG9 | 47.2 |
| SNPM04476 | LG9 | 47.2 |
| SNPM02019 | LG9 | 54.5 |
| SNPM03731 | LG9 | 55.6 |
| SNPM02777 | LG9 | 58.0 |
| SNPM03579 | LG9 | 59.3 |
| SNPM00923 | LG9 | 60.7 |
| SNPM00922 | LG9 | 60.7 |
| SNPM02050 | LG9 | 62.8 |
| SNPM01102 | LG9 | 68.6 |
| SNPM04339 | LG9 | 68.6 |
| SNPM02042 | LG9 | 70.0 |
| SNPM00187 | LG9 | 70.0 |
| sEg00167 | LG9 | 71.1 |
| SNPM04505 | LG9 | 71.7 |
| sMg00175 | LG9 | 73.4 |
| sEg00048 | LG9 | 73.4 |
| SNPM00226 | LG9 | 77.1 |
| SNPM01471 | LG9 | 79.8 |
| SNPM02749 | LG9 | 79.8 |
| SNPM01997 | LG9 | 83.5 |
| SNPM02450 | LG9 | 83.5 |
| SNPM02230 | LG9 | 83.5 |
| SNPM04094 | LG10 | 0.0 |
| SNPM02007 | LG10 | 0.0 |
| SNPM04259 | LG10 | 0.0 |
| SNPM04578 | LG10 | 0.0 |
| SNPM02480 | LG10 | 0.0 |
| SNPM03586 | LG10 | 0.0 |
| SNPM03982 | LG10 | 0.0 |
| SNPM02914 | LG10 | 0.0 |
| SNPM04260 | LG10 | 0.0 |
| SNPM03895 | LG10 | 0.0 |
| SNPM01713 | LG10 | 0.0 |
| SNPM04137 | LG10 | 0.0 |
| SNPM02273 | LG10 | 0.0 |
| SNPM00505 | LG10 | 0.0 |
| SNPM01230 | LG10 | 1.3 |
| SNPM03971 | LG10 | 1.4 |
| sMo00088 | LG10 | 1.4 |
| SNPM01184 | LG10 | 3.9 |
| SNPM01094 | LG10 | 3.9 |
| SNPM03559 | LG10 | 3.9 |
| SNPM02468 | LG10 | 3.9 |
| SNPM04403 | LG10 | 3.9 |
| SNPM03440 | LG10 | 7.1 |
| SNPM03713A | LG10 | 7.8 |
| sMo00257 | LG10 | 8.4 |
| SNPM02414 | LG10 | 10.5 |
| SNPM03561 | LG10 | 10.5 |
| SNPM00478 | LG10 | 10.5 |
| SNPM00577 | LG10 | 10.5 |
| SNPM00397 | LG10 | 11.0 |
| SNPM01635 | LG10 | 12.2 |
| SNPM00530 | LG10 | 12.2 |
| SNPM02508 | LG10 | 12.2 |
| SNPM00345 | LG10 | 12.2 |
| SNPM03848 | LG10 | 14.4 |
| SNPM02779 | LG10 | 19.2 |
| SNPM01664 | LG10 | 19.7 |
| SNPM02671 | LG10 | 20.8 |
| SNPM00322 | LG10 | 21.4 |
| SNPM03873 | LG10 | 21.4 |
| SNPM02021 | LG10 | 21.4 |
| SNPM03656 | LG10 | 23.1 |
| sEg00010 | LG10 | 26.0 |
| SNPM01169 | LG10 | 26.9 |
| sMo00226 | LG10 | 28.8 |
| sEg00117 | LG10 | 31.7 |
| SNPM03180 | LG10 | 33.5 |
| SNPM03643 | LG10 | 33.5 |
| SNPM01430b | LG10 | 34.0 |
| SNPM03611 | LG10 | 34.4 |
| SNPM04299 | LG10 | 34.4 |
| SNPM00141 | LG10 | 34.4 |
| SNPM01539 | LG10 | 34.4 |
| SNPM01515 | LG10 | 34.4 |
| SNPM01123 | LG10 | 34.4 |
| SNPM03893 | LG10 | 34.4 |
| SNPM01336 | LG10 | 34.4 |
| SNPM03610 | LG10 | 34.4 |
| SNPM00816b | LG10 | 35.0 |
| SNPM04133 | LG10 | 35.5 |
| SNPM01432 | LG10 | 36.1 |
| SNPM03894 | LG10 | 36.6 |
| SNPM00499 | LG10 | 36.6 |
| SNPM01228 | LG10 | 38.9 |
| SNPM04162 | LG10 | 38.9 |
| SNPM00281 | LG10 | 38.9 |
| SNPM03108 | LG10 | 40.2 |
| SNPM03121 | LG10 | 40.2 |
| SNPM03901 | LG10 | 40.2 |
| SNPM02660 | LG10 | 40.7 |
| SNPM00486 | LG10 | 40.7 |
| SNPM04423 | LG10 | 40.7 |
| SNPM03218 | LG10 | 42.4 |
| SNPM01239 | LG10 | 42.4 |
| SNPM01776 | LG10 | 42.4 |
| SNPM01068 | LG10 | 43.3 |
| SNPM04575 | LG10 | 43.3 |
| SNPM01173 | LG10 | 43.3 |
| SNPM04488 | LG10 | 43.3 |
| SNPM01224 | LG10 | 43.3 |
| SNPM04633 | LG10 | 43.3 |
| SNPM04489 | LG10 | 43.3 |
| SNPM01225 | LG10 | 43.3 |
| SNPM01067 | LG10 | 43.3 |
| SNPM03509 | LG10 | 47.4 |
| SNPM01933 | LG10 | 51.5 |
| SNPM02712 | LG10 | 51.5 |
| SNPM00047 | LG10 | 53.5 |
| SNPM04279 | LG10 | 55.5 |
| SNPM02675 | LG10 | 60.3 |
| SNPM04483 | LG10 | 60.3 |
| SNPM03371 | LG10 | 60.3 |
| Hght4_oSSR | LG10 | 60.7 |
| SNPM02210 | LG10 | 63.7 |
| SNPM00442 | LG10 | 63.7 |
| SNPM00068 | LG10 | 66.7 |
| SNPM04172 | LG10 | 66.7 |
| SNPM00648 | LG10 | 67.9 |
| SNPM00819 | LG10 | 70.3 |
| SNPM04110 | LG10 | 71.0 |
| SNPM00266 | LG10 | 71.7 |
| SNPM01257 | LG10 | 71.7 |
| SNPM04550 | LG10 | 71.7 |
| SNPM04551 | LG10 | 74.5 |
| sEg00017 | LG10 | 74.5 |
| SNPM01000 | LG10 | 74.5 |
| SNPM02548 | LG10 | 74.9 |
| SNPM02558 | LG10 | 74.9 |
| SNPM03145 | LG10 | 75.7 |
| SNPM03962 | LG10 | 75.7 |
| SNPM03963 | LG10 | 75.7 |
| SNPM03921 | LG10 | 77.4 |
| SNPM01401 | LG10 | 77.4 |
| SNPM01277 | LG10 | 79.1 |
| SNPM03764 | LG10 | 81.5 |
| SNPM02563 | LG10 | 81.5 |
| SNPM00578 | LG10 | 81.5 |
| SNPM03564 | LG10 | 83.4 |
| SNPM02856 | LG10 | 87.3 |
| SNPM01360 | LG10 | 87.3 |
| SNPM02572 | LG10 | 87.3 |
| SNPM02974 | LG10 | 95.8 |
| SNPM02201 | LG10 | 98.7 |
| SNPM04650 | LG10 | 99.6 |
| SNPM04899 | LG10 | 105.4 |
| SNPM02546 | LG10 | 105.4 |
| SNPM03473 | LG10 | 105.8 |
| SNPM03638 | LG10 | 106.4 |
| SNPM02107 | LG10 | 106.4 |
| SNPM00132 | LG10 | 108.4 |
| SNPM00470 | LG10 | 108.4 |
| SNPM04820 | LG10 | 111.2 |
| SNPM03247 | LG10 | 111.8 |
| SNPM00329 | LG10 | 111.8 |
| SNPM00354 | LG10 | 111.8 |
| SNPM04450 | LG10 | 113.0 |
| SNPM02373 | LG10 | 114.0 |
| SNPM03947 | LG10 | 116.3 |
| sMg00168B | LG11 | 0.0 |
| SNPM00999 | LG11 | 1.4 |
| SNPM02837 | LG11 | 1.4 |
| SNPM02445 | LG11 | 1.4 |
| SNPM00174 | LG11 | 1.4 |
| SNPM04092 | LG11 | 1.4 |
| SNPM01732 | LG11 | 1.4 |
| SNPM04901 | LG11 | 1.4 |
| SNPM04216 | LG11 | 1.4 |
| sMg00030 | LG11 | 1.4 |
| SNPM03534 | LG11 | 1.4 |
| SNPM03840 | LG11 | 1.4 |
| SNPM04053 | LG11 | 1.4 |
| SNPM03426 | LG11 | 2.1 |
| SNPM01206 | LG11 | 2.8 |
| SNPM02851 | LG11 | 3.6 |
| SNPM03876 | LG11 | 3.6 |
| SNPM00863 | LG11 | 3.6 |
| SNPM00864 | LG11 | 3.6 |
| SNPM02738 | LG11 | 3.6 |
| sMo00077A | LG11 | 5.2 |
| SNPM03663 | LG11 | 5.9 |
| SNPM03082 | LG11 | 7.5 |
| SNPM00264 | LG11 | 8.1 |
| SNPM04209 | LG11 | 8.1 |
| SNPM00392 | LG11 | 8.1 |
| SNPM02668 | LG11 | 8.1 |
| SNPM00142 | LG11 | 8.1 |
| SNPM04418 | LG11 | 8.1 |
| sMo00077B | LG11 | 9.0 |
| SNPM02134 | LG11 | 9.9 |
| sEg00157 | LG11 | 11.8 |
| SNPM01390 | LG11 | 13.6 |
| SNPM04306 | LG11 | 14.5 |
| SNPM01738 | LG11 | 14.5 |
| SNPM03132 | LG11 | 14.5 |
| SNPM04751 | LG11 | 15.8 |
| SNPM04538 | LG11 | 15.8 |
| SNPM00126 | LG11 | 15.8 |
| SNPM03346 | LG11 | 15.8 |
| SNPM01338 | LG11 | 16.2 |
| SNPM03217 | LG11 | 16.7 |
| SNPM04389 | LG11 | 17.1 |
| SNPM02263 | LG11 | 17.6 |
| SNPM02832 | LG11 | 17.6 |
| SNPM00395 | LG11 | 18.4 |
| SNPM04455 | LG11 | 18.9 |
| SNPM02354 | LG11 | 19.8 |
| SNPM01460 | LG11 | 19.8 |
| SNPM00763 | LG11 | 19.8 |
| SNPM02231 | LG11 | 19.8 |
| SNPM00240 | LG11 | 19.8 |
| SNPM02917 | LG11 | 21.2 |
| SNPM03566 | LG11 | 21.2 |
| SNPM03038 | LG11 | 24.0 |
| SNPM00370 | LG11 | 24.7 |
| SNPM01249 | LG11 | 24.7 |
| SNPM04288 | LG11 | 24.7 |
| SNPM04693 | LG11 | 24.7 |
| SNPM04720 | LG11 | 25.4 |
| SNPM04064 | LG11 | 25.4 |
| SNPM00334 | LG11 | 26.1 |
| SNPM01326 | LG11 | 26.1 |
| SNPM00466 | LG11 | 28.7 |
| SNPM00955 | LG11 | 28.7 |
| SNPM02566 | LG11 | 28.7 |
| SNPM00920 | LG11 | 29.4 |
| SNPM01624 | LG11 | 37.4 |
| SNPM00559 | LG11 | 37.4 |
| SNPM03257 | LG11 | 37.4 |
| SNPM02315 | LG11 | 37.4 |
| SNPM03676 | LG11 | 38.7 |
| SNPM00323 | LG11 | 38.7 |
| SNPM03677 | LG11 | 40.3 |
| SNPM01781 | LG11 | 40.3 |
| SNPM02858 | LG11 | 40.3 |
| SNPM01590 | LG11 | 40.3 |
| SNPM03501 | LG11 | 40.3 |
| SNPM00457 | LG11 | 42.1 |
| SNPM04546 | LG11 | 43.7 |
| SNPM01865 | LG11 | 45.7 |
| SNPM03233 | LG11 | 45.7 |
| SNPM04463 | LG11 | 45.7 |
| SNPM02902 | LG11 | 45.7 |
| SNPM05019 | LG11 | 45.7 |
| SNPM00162 | LG11 | 45.7 |
| SNPM03431 | LG11 | 45.7 |
| SNPM03533 | LG11 | 46.4 |
| SNPM04042 | LG11 | 46.4 |
| SNPM00222 | LG11 | 46.4 |
| SNPM04049 | LG11 | 46.4 |
| SNPM04956 | LG11 | 46.4 |
| SNPM00510 | LG11 | 47.4 |
| SNPM04622 | LG11 | 47.4 |
| SNPM00201 | LG11 | 47.4 |
| SNPM03821 | LG11 | 47.4 |
| SNPM02386 | LG11 | 47.4 |
| SNPM01966 | LG11 | 47.4 |
| sEg00019 | LG11 | 47.4 |
| SNPM02353 | LG11 | 50.4 |
| SNPM01085 | LG11 | 50.4 |
| SNPM02533 | LG11 | 50.4 |
| SNPM02472 | LG11 | 50.4 |
| SNPM00211 | LG11 | 50.4 |
| SNPM04562 | LG11 | 50.4 |
| SNPM04446 | LG11 | 50.4 |
| SNPM04955 | LG11 | 50.4 |
| SNPM02161 | LG11 | 50.4 |
| SNPM03288 | LG11 | 50.4 |
| SNPM02685 | LG11 | 50.4 |
| SNPM04036 | LG11 | 53.3 |
| SNPM03650 | LG11 | 54.8 |
| SNPM02804 | LG11 | 55.0 |
| SNPM02805 | LG11 | 55.1 |
| SNPM03465 | LG11 | 55.3 |
| sMg00120 | LG11 | 57.0 |
| SNPM00925 | LG11 | 59.0 |
| SNPM04882 | LG11 | 59.3 |
| SNPM02605A | LG11 | 59.7 |
| SNPM00024 | LG11 | 59.7 |
| SNPE00437 | LG11 | 63.4 |
| SNPM03109 | LG11 | 63.4 |
| sMo00185 | LG11 | 63.4 |
| SNPM00254 | LG11 | 64.2 |
| SNPM00279 | LG11 | 65.9 |
| SNPM05063 | LG11 | 65.9 |
| SNPM01119 | LG11 | 65.9 |
| SNPM02996 | LG11 | 65.9 |
| SNPM04071 | LG11 | 73.3 |
| SNPM04957 | LG11 | 76.0 |
| SNPM01944 | LG11 | 76.0 |
| SNPM02721 | LG11 | 76.6 |
| SNPM01196 | LG11 | 76.6 |
| SNPM01681 | LG11 | 77.5 |
| SNPM01202 | LG11 | 80.8 |
| SNPM01147 | LG11 | 81.9 |
| SNPM00856 | LG11 | 82.3 |
| SNPM01059 | LG11 | 83.6 |
| SNPM00052 | LG11 | 85.0 |
| SNPM03097 | LG11 | 85.0 |
| SNPM03476 | LG11 | 89.6 |
| SNPM00638 | LG11 | 89.6 |
| SNPM01566 | LG11 | 91.4 |
| SNPM02157 | LG11 | 91.4 |
| SNPM01125 | LG11 | 95.5 |
| SNPM01499 | LG11 | 96.4 |
| SNPM00328 | LG11 | 97.2 |
| SNPM03751 | LG11 | 97.2 |
| SNPM01897 | LG11 | 97.2 |
| sMg00093 | LG11 | 100.5 |
| SNPM03871 | LG11 | 105.2 |
| SNPM00160 | LG11 | 105.2 |
| SNPM03628 | LG11 | 105.2 |
| SNPM00576 | LG11 | 105.2 |
| SNPM03547 | LG11 | 105.2 |
| SNPM03700 | LG11 | 105.2 |
| SNPM01496 | LG11 | 106.5 |
| SNPM00433 | LG11 | 107.9 |
| SNPM02148 | LG11 | 112.4 |
| SNPM02131 | LG11 | 113.5 |
| SNPM02952 | LG11 | 116.6 |
| SNPM00445 | LG11 | 117.7 |
| SNPM01533 | LG11 | 117.7 |
| SNPM04942 | LG11 | 118.9 |
| SNPM00188 | LG11 | 118.9 |
| SNPM00284 | LG11 | 120.8 |
| SNPM04811 | LG11 | 122.7 |
| SNPM02466 | LG11 | 127.6 |
| SNPM03598 | LG11 | 128.7 |
| SNPM04853 | LG11 | 133.8 |
| SNPM00526 | LG11 | 134.7 |
| SNPM02153 | LG11 | 138.8 |
| SNPM01967 | LG11 | 138.8 |
| SNPM03773 | LG11 | 139.4 |
| SNPM02423 | LG11 | 140.6 |
| SNPM00204 | LG11 | 142.6 |
| SNPM03195 | LG11 | 145.6 |
| SNPM04867 | LG11 | 145.6 |
| SNPM00518 | LG11 | 145.6 |
| SNPM01378 | LG12 | 0.0 |
| SNPM02194 | LG12 | 0.0 |
| SNPM03744 | LG12 | 0.7 |
| SNPM00082 | LG12 | 0.7 |
| SNPM03826 | LG12 | 0.7 |
| SNPM01233 | LG12 | 2.0 |
| SNPM00448 | LG12 | 2.4 |
| SNPM02542 | LG12 | 2.4 |
| SNPM04746 | LG12 | 2.9 |
| SNPM01746 | LG12 | 3.0 |
| SNPM04964b | LG12 | 3.1 |
| SNPM02877 | LG12 | 3.2 |
| SNPM01083 | LG12 | 3.8 |
| SNPM01092 | LG12 | 3.8 |
| SNPM03850 | LG12 | 3.8 |
| SNPM03851 | LG12 | 3.8 |
| SNPM00896 | LG12 | 5.6 |
| SNPM01344 | LG12 | 5.6 |
| SNPM01264 | LG12 | 5.6 |
| SNPM01385 | LG12 | 5.6 |
| SNPM01343 | LG12 | 5.6 |
| SNPM02476 | LG12 | 5.6 |
| SNPM01425 | LG12 | 5.6 |
| SNPM04950 | LG12 | 5.6 |
| SNPM01629 | LG12 | 5.6 |
| SNPM03039 | LG12 | 5.6 |
| SNPM03994 | LG12 | 5.6 |
| SNPM03327 | LG12 | 6.1 |
| SNPM01117 | LG12 | 9.2 |
| SNPM00168 | LG12 | 9.2 |
| SNPM00381 | LG12 | 9.2 |
| SNPM03500 | LG12 | 9.2 |
| SNPM03275 | LG12 | 10.0 |
| SNPM04653 | LG12 | 13.5 |
| SNPM02876 | LG12 | 14.7 |
| SNPM03341 | LG12 | 16.6 |
| SNPM02026 | LG12 | 16.7 |
| SNPM01314 | LG12 | 20.7 |
| SNPM04292 | LG12 | 22.0 |
| SNPM00311 | LG12 | 22.0 |
| SNPM01685 | LG12 | 22.0 |
| SNPM03342 | LG12 | 22.0 |
| SNPM00441 | LG12 | 22.0 |
| SNPM02746 | LG12 | 22.0 |
| SNPM02108 | LG12 | 23.8 |
| SNPM05026 | LG12 | 23.8 |
| SNPM00952 | LG12 | 23.8 |
| SNPM02891 | LG12 | 23.8 |
| SNPM02237 | LG12 | 23.8 |
| SNPM00597 | LG12 | 26.5 |
| SNPM01178 | LG12 | 27.4 |
| SNPM00274 | LG12 | 31.4 |
| SNPM01887 | LG12 | 32.8 |
| SNPM01087 | LG12 | 32.8 |
| SNPM00431 | LG12 | 32.8 |
| SNPM04208 | LG12 | 34.2 |
| SNPM04207 | LG12 | 34.2 |
| SNPM02208 | LG12 | 38.2 |
| SNPM04368b | LG12 | 39.3 |
| SNPM01426 | LG12 | 40.4 |
| SNPM04056 | LG12 | 40.4 |
| SNPM02308 | LG12 | 40.4 |
| sEg00126 | LG12 | 42.5 |
| SNPM04799 | LG12 | 43.7 |
| sEg00045 | LG12 | 43.7 |
| SNPM00237 | LG12 | 45.8 |
| SNPM02067 | LG12 | 46.3 |
| SNPM03147 | LG12 | 46.3 |
| SNPM00056 | LG12 | 46.3 |
| SNPM00571 | LG12 | 47.7 |
| SNPM01591 | LG12 | 49.8 |
| SNPM01222 | LG12 | 50.3 |
| SNPM03333 | LG12 | 50.3 |
| SNPM02044 | LG12 | 50.6 |
| SNPM00158 | LG12 | 50.8 |
| SNPM01108 | LG12 | 50.8 |
| SNPM00278 | LG12 | 51.2 |
| sMg00014 | LG12 | 51.2 |
| SNPM00459 | LG12 | 52.6 |
| sMg00046 | LG12 | 53.3 |
| SNPM01822 | LG12 | 54.7 |
| SNPM03412 | LG12 | 54.7 |
| SNPM01707 | LG12 | 56.2 |
| sMg00215 | LG12 | 56.2 |
| SNPM02222 | LG12 | 56.2 |
| SNPM00677 | LG12 | 56.2 |
| sMo00161A | LG12 | 56.2 |
| SNPM03704 | LG12 | 56.9 |
| SNPM04694 | LG12 | 57.5 |
| SNPM04695 | LG12 | 57.5 |
| SNPM04168 | LG12 | 58.5 |
| SNPM03194 | LG12 | 61.7 |
| SNPM00454 | LG12 | 62.5 |
| SNPM03755 | LG12 | 62.5 |
| SNPM03608 | LG12 | 64.3 |
| SNPM02415 | LG12 | 66.7 |
| SNPM01242 | LG12 | 66.7 |
| SNPM02327 | LG12 | 67.9 |
| sMg00066 | LG12 | 74.5 |
| SNPM03587 | LG12 | 75.2 |
| sMo00055 | LG12 | 75.2 |
| SNPM04822 | LG12 | 76.2 |
| SNPM04998 | LG12 | 76.2 |
| SNPM01743 | LG12 | 77.1 |
| SNPM04870 | LG12 | 78.4 |
| SNPM01103 | LG12 | 83.9 |
| SNPM04278 | LG12 | 84.4 |
| SNPM00762 | LG12 | 84.4 |
| SNPM01464 | LG12 | 84.4 |
| SNPM04609 | LG12 | 84.4 |
| SNPM00231 | LG12 | 84.4 |
| SNPM00011 | LG12 | 84.4 |
| SNPM01855 | LG12 | 85.2 |
| sEg00076 | LG12 | 86.8 |
| SNPM01975 | LG12 | 89.8 |
| SNPM03212 | LG12 | 89.8 |
| SNPM00313 | LG12 | 90.7 |
| SNPM03507 | LG12 | 90.7 |
| SNPM03071 | LG12 | 90.7 |
| SNPM00506 | LG12 | 90.7 |
| SNPM02306 | LG12 | 93.8 |
| SNPM03365 | LG12 | 93.8 |
| SNPM01941 | LG12 | 94.7 |
| SNPM02123 | LG12 | 97.5 |
| SNPM00501 | LG12 | 98.3 |
| SNPM01770 | LG12 | 98.3 |
| sEg00080 | LG12 | 98.3 |
| sMg00049 | LG12 | 101.1 |
| SNPM02892 | LG12 | 102.2 |
| SNPM00849 | LG12 | 104.4 |
| SNPM02133 | LG12 | 105.1 |
| SNPM04017 | LG12 | 105.1 |
| SNPM00239 | LG12 | 105.1 |
| SNPM00081 | LG12 | 106.1 |
| SNPE00401 | LG12 | 106.1 |
| SNPM04630 | LG12 | 112.2 |
| SNPM00135 | LG12 | 113.7 |
| sMg00176 | LG12 | 118.0 |
| SNPM02739 | LG12 | 119.7 |
| SNPM04608 | LG12 | 121.9 |
| SNPM04061 | LG12 | 122.5 |
| SNPM01809 | LG12 | 123.7 |
| SNPM00159 | LG12 | 123.7 |
| SNPM01367 | LG12 | 124.1 |
| SNPM04517 | LG13 | 0.0 |
| SNPM00822 | LG13 | 0.0 |
| SNPM03451 | LG13 | 0.7 |
| SNPM01311 | LG13 | 0.7 |
| SNPM03024 | LG13 | 0.7 |
| SNPM03450 | LG13 | 0.7 |
| SNPM03512 | LG13 | 0.7 |
| SNPM04040 | LG13 | 1.3 |
| SNPM01752 | LG13 | 1.3 |
| SNPM04684 | LG13 | 1.3 |
| SNPM01267 | LG13 | 1.3 |
| SNPM04041 | LG13 | 1.3 |
| SNPM03568 | LG13 | 2.0 |
| SNPM05005 | LG13 | 3.3 |
| SNPM03961 | LG13 | 3.3 |
| SNPM04655 | LG13 | 3.3 |
| SNPM04323 | LG13 | 3.3 |
| SNPM04322 | LG13 | 3.3 |
| SNPM00605 | LG13 | 3.3 |
| SNPM03380 | LG13 | 5.5 |
| SNPM04344 | LG13 | 5.5 |
| SNPM01204 | LG13 | 5.5 |
| SNPM01215 | LG13 | 6.9 |
| SNPM01955 | LG13 | 6.9 |
| sMg00063 | LG13 | 7.5 |
| sEg00151 | LG13 | 8.2 |
| SNPM02279 | LG13 | 9.5 |
| SNPM00612 | LG13 | 9.5 |
| SNPM01751 | LG13 | 9.5 |
| SNPM01761 | LG13 | 9.5 |
| SNPM01511 | LG13 | 9.5 |
| SNPM00382 | LG13 | 9.5 |
| SNPM00888 | LG13 | 9.5 |
| SNPM02511 | LG13 | 10.4 |
| SNPM02455 | LG13 | 10.4 |
| SNPM01245 | LG13 | 10.4 |
| SNPM04364 | LG13 | 11.3 |
| SNPM04143 | LG13 | 11.3 |
| SNPM03946 | LG13 | 11.3 |
| SNPM03522 | LG13 | 12.2 |
| SNPM02514 | LG13 | 12.2 |
| SNPM03530 | LG13 | 12.2 |
| SNPM00250 | LG13 | 12.2 |
| SNPM04451 | LG13 | 12.2 |
| SNPM02672 | LG13 | 12.2 |
| SNPM04452 | LG13 | 12.2 |
| SNPM01637 | LG13 | 12.2 |
| SNPM02325 | LG13 | 15.2 |
| SNPM01871 | LG13 | 15.2 |
| SNPM02842 | LG13 | 15.8 |
| SNPM02246 | LG13 | 15.8 |
| SNPM01137 | LG13 | 15.8 |
| SNPM03905 | LG13 | 18.0 |
| SNPM02173 | LG13 | 20.7 |
| SNPM04457 | LG13 | 20.7 |
| SNPM02172 | LG13 | 20.7 |
| SNPM03021 | LG13 | 21.2 |
| SNPM03544 | LG13 | 21.2 |
| SNPM04188 | LG13 | 21.2 |
| SNPM04187 | LG13 | 21.2 |
| SNPM00600 | LG13 | 21.2 |
| SNPM02977 | LG13 | 22.7 |
| SNPM02261 | LG13 | 22.7 |
| SNPM01940 | LG13 | 23.5 |
| SNPM04592 | LG13 | 28.0 |
| SNPM01155 | LG13 | 29.5 |
| SNPM02170b | LG13 | 29.5 |
| SNPM04171 | LG13 | 29.5 |
| SNPM00181 | LG13 | 30.2 |
| SNPM01514 | LG13 | 30.8 |
| SNPM02087 | LG13 | 31.5 |
| SNPM01547 | LG13 | 32.4 |
| SNPM00220 | LG13 | 34.6 |
| SNPM03897 | LG13 | 35.9 |
| SNPM03238 | LG13 | 35.9 |
| SNPM02557 | LG13 | 37.1 |
| SNPM01565 | LG13 | 37.7 |
| SNPM03550 | LG13 | 38.2 |
| SNPM03551 | LG13 | 38.2 |
| SNPM04568 | LG13 | 40.6 |
| SNPM04983 | LG13 | 40.8 |
| sMg00152 | LG13 | 43.0 |
| SNPM04890 | LG13 | 44.2 |
| SNPM04268 | LG13 | 44.2 |
| SNPM01428 | LG13 | 44.2 |
| SNPM01964 | LG13 | 44.2 |
| SNPM03445 | LG13 | 46.6 |
| SNPM01882 | LG13 | 47.1 |
| SNPM00361 | LG13 | 47.1 |
| SNPM04704 | LG13 | 49.1 |
| SNPM02964 | LG13 | 49.2 |
| SNPM00691 | LG13 | 49.2 |
| SNPM00205 | LG13 | 50.7 |
| SNPM04473 | LG13 | 51.7 |
| SNPM05009 | LG13 | 53.7 |
| SNPM00544 | LG13 | 53.7 |
| SNPM01467 | LG13 | 53.7 |
| SNPM03870 | LG13 | 54.8 |
| SNPM01843 | LG13 | 54.8 |
| SNPM00291 | LG13 | 55.6 |
| SNPM01723 | LG13 | 57.1 |
| SNPM05034 | LG13 | 57.8 |
| SNPM00252 | LG13 | 59.4 |
| SNPM04203 | LG13 | 61.6 |
| SNPM03083 | LG13 | 61.6 |
| SNPM02862 | LG13 | 61.6 |
| SNPM03191 | LG13 | 63.9 |
| SNPM03799 | LG13 | 65.7 |
| SNPM04826 | LG13 | 65.7 |
| SNPM00508 | LG13 | 66.7 |
| SNPM04607 | LG13 | 66.7 |
| SNPM00256 | LG13 | 66.7 |
| SNPM04358 | LG13 | 68.8 |
| SNPM03590 | LG13 | 68.8 |
| SNPM04739 | LG13 | 68.8 |
| SNPM00904 | LG13 | 72.0 |
| sMg00254A | LG13 | 72.0 |
| SNPM04375 | LG13 | 72.0 |
| SNPM01172 | LG13 | 75.6 |
| sEg00197 | LG13 | 75.6 |
| SNPM02640 | LG13 | 75.6 |
| mEgCIR3555 | LG13 | 75.6 |
| SNPM00276 | LG13 | 77.0 |
| SNPM02183 | LG13 | 77.0 |
| SNPM00473 | LG13 | 77.0 |
| sEg00011 | LG13 | 77.3 |
| SNPM01878 | LG13 | 77.6 |
| SNPM02147 | LG13 | 79.0 |
| SNPM01176 | LG13 | 79.0 |
| SNPM05000 | LG13 | 79.0 |
| SNPM04316 | LG13 | 79.0 |
| SNPM00921 | LG13 | 79.0 |
| sMo00196B | LG13 | 80.2 |
| sMg00254B | LG13 | 80.2 |
| sMo00166 | LG13 | 80.3 |
| SNPM01457 | LG14 | 0.0 |
| SNPM04600 | LG14 | 0.0 |
| SNPM00178 | LG14 | 0.0 |
| SNPM00964 | LG14 | 0.5 |
| SNPM00403 | LG14 | 0.5 |
| SNPM00398 | LG14 | 1.1 |
| SNPM04082 | LG14 | 1.3 |
| SNPM04376 | LG14 | 1.5 |
| SNPM01883 | LG14 | 2.0 |
| sMo00023 | LG14 | 2.0 |
| SNPM01736 | LG14 | 4.0 |
| SNPM01938 | LG14 | 4.0 |
| SNPM01111 | LG14 | 5.2 |
| SNPM01772 | LG14 | 5.2 |
| sMo00023a | LG14 | 5.9 |
| SNPM02154 | LG14 | 6.6 |
| SNPM00263 | LG14 | 6.8 |
| SNPM02431 | LG14 | 7.0 |
| sMo00187 | LG14 | 8.0 |
| sMg00074 | LG14 | 8.0 |
| SNPM00299 | LG14 | 9.8 |
| SNPM02801 | LG14 | 9.8 |
| sMo00063 | LG14 | 10.8 |
| sMo00066 | LG14 | 14.8 |
| SNPM03357 | LG14 | 19.5 |
| SNPM00891 | LG14 | 20.0 |
| SNPM00156 | LG14 | 20.0 |
| SNPM02628 | LG14 | 22.3 |
| SNPM04099 | LG14 | 24.0 |
| SNPM00786 | LG14 | 24.8 |
| SNPM01932 | LG14 | 26.5 |
| SNPM03266 | LG14 | 28.2 |
| SNPM00525 | LG14 | 28.2 |
| SNPM00225 | LG14 | 28.3 |
| SNPM01841 | LG14 | 28.7 |
| SNPM01046 | LG14 | 29.4 |
| SNPM01734 | LG14 | 29.4 |
| SNPM00389 | LG14 | 30.5 |
| SNPM02846 | LG14 | 31.1 |
| SNPM01156 | LG14 | 31.7 |
| SNPM00369 | LG14 | 33.8 |
| SNPM00136 | LG14 | 36.6 |
| SNPM00524 | LG14 | 38.2 |
| SNPM00295 | LG14 | 41.1 |
| SNPM00455 | LG14 | 41.1 |
| SNPM01914 | LG14 | 41.1 |
| sMo00065 | LG14 | 43.5 |
| SNPM02254 | LG14 | 44.8 |
| SNPM03954 | LG14 | 45.9 |
| SNPM02963 | LG14 | 48.3 |
| SNPM02209 | LG14 | 48.3 |
| SNPM00230 | LG14 | 50.1 |
| SNPM04938 | LG14 | 50.8 |
| SNPM03733 | LG14 | 51.1 |
| SNPM04522 | LG14 | 51.1 |
| sMg00221 | LG14 | 55.3 |
| SNPM04636 | LG14 | 56.9 |
| SNPM04637 | LG14 | 56.9 |
| SNPM02290 | LG14 | 60.0 |
| SNPM02086 | LG14 | 64.7 |
| SNPM03065 | LG14 | 64.7 |
| SNPM01414 | LG14 | 64.7 |
| SNPM04700 | LG14 | 65.1 |
| SNPM01907 | LG14 | 66.6 |
| SNPM02985 | LG14 | 67.4 |
| SNPM00236 | LG14 | 67.4 |
| SNPM03252 | LG14 | 67.4 |
| SNPM01287 | LG14 | 68.1 |
| SNPM03281 | LG14 | 68.5 |
| SNPM03280 | LG14 | 68.5 |
| SNPM04009 | LG14 | 69.6 |
| SNPM03612 | LG14 | 69.6 |
| SNPM00724 | LG14 | 69.6 |
| SNPM03060 | LG14 | 69.6 |
| sMg00079 | LG14 | 69.6 |
| SNPM02144 | LG14 | 69.6 |
| SNPM00697 | LG14 | 73.9 |
| SNPM00479 | LG14 | 73.9 |
| SNPM01377 | LG14 | 73.9 |
| SNPM03730 | LG14 | 74.0 |
| SNPM00192 | LG14 | 74.5 |
| SNPM01868 | LG14 | 75.7 |
| SNPM01120 | LG14 | 76.9 |
| SNPM01692 | LG14 | 76.9 |
| SNPM03120 | LG14 | 82.6 |
| SNPE00434 | LG14 | 93.3 |
| SNPM02322 | LG14 | 93.3 |
| SNPM00551 | LG14 | 93.3 |
| SNPM01874 | LG14 | 93.3 |
| SNPM03790 | LG14 | 97.0 |
| SNPM01186 | LG14 | 97.0 |
| SNPM04350 | LG14 | 97.8 |
| SNPM00044 | LG14 | 100.0 |
| SNPM02785 | LG14 | 100.0 |
| sMg00087B | LG15 | 0.0 |
| sMg00193 | LG15 | 4.2 |
| SNPM02667 | LG15 | 4.9 |
| SNPM03250 | LG15 | 5.6 |
| SNPM01282 | LG15 | 5.6 |
| SNPM01300 | LG15 | 5.6 |
| SNPM01299 | LG15 | 5.6 |
| SNPM00602 | LG15 | 5.6 |
| SNPM02358 | LG15 | 5.6 |
| SNPM04662 | LG15 | 5.6 |
| SNPM03155 | LG15 | 5.6 |
| SNPM04120 | LG15 | 5.6 |
| SNPM01417 | LG15 | 5.6 |
| SNPM02684 | LG15 | 5.6 |
| SNPM01451 | LG15 | 5.6 |
| SNPM04661 | LG15 | 5.6 |
| SNPM04421 | LG15 | 6.2 |
| SNPM04422 | LG15 | 6.2 |
| SNPM04031 | LG15 | 6.7 |
| SNPM01071 | LG15 | 6.7 |
| SNPM00330 | LG15 | 7.3 |
| SNPM00502 | LG15 | 7.3 |
| SNPM04565 | LG15 | 7.3 |
| SNPM01306 | LG15 | 7.3 |
| SNPM04564 | LG15 | 7.3 |
| SNPM02631 | LG15 | 8.0 |
| SNPM02814 | LG15 | 8.7 |
| SNPM02122 | LG15 | 8.7 |
| SNPM04924 | LG15 | 8.7 |
| SNPM01660 | LG15 | 8.7 |
| SNPM01652 | LG15 | 8.7 |
| SNPM04614 | LG15 | 8.7 |
| SNPM02117 | LG15 | 8.7 |
| SNPM03609 | LG15 | 8.7 |
| SNPM04290 | LG15 | 8.7 |
| SNPM03630 | LG15 | 8.7 |
| SNPM00767 | LG15 | 8.7 |
| SNPM03174 | LG15 | 8.7 |
| SNPM00941 | LG15 | 8.7 |
| SNPM02821 | LG15 | 8.7 |
| SNPM02810 | LG15 | 8.7 |
| SNPM01651 | LG15 | 8.7 |
| sMg00087A | LG15 | 9.6 |
| SNPM03945 | LG15 | 10.4 |
| sMo00301 | LG15 | 10.4 |
| SNPM03364 | LG15 | 10.4 |
| SNPM03084 | LG15 | 11.0 |
| SNPM03205 | LG15 | 11.6 |
| SNPM02822 | LG15 | 11.6 |
| sMo00027 | LG15 | 11.6 |
| SNPM04615 | LG15 | 11.6 |
| SNPM03407 | LG15 | 11.6 |
| SNPM02962 | LG15 | 12.7 |
| SNPM01393 | LG15 | 12.7 |
| SNPM04081 | LG15 | 12.7 |
| SNPM02587 | LG15 | 12.7 |
| SNPM02726 | LG15 | 13.8 |
| SNPM02329 | LG15 | 14.9 |
| SNPM04381b | LG15 | 14.9 |
| SNPM01745 | LG15 | 14.9 |
| SNPM00703 | LG15 | 15.4 |
| SNPM00407 | LG15 | 15.4 |
| SNPM04660 | LG15 | 15.4 |
| sMo00198 | LG15 | 17.3 |
| SNPM03090 | LG15 | 19.2 |
| SNPM00347 | LG15 | 20.8 |
| SNPM02416 | LG15 | 20.8 |
| SNPM00083 | LG15 | 20.8 |
| SNPM02292 | LG15 | 22.5 |
| SNPM02293 | LG15 | 22.5 |
| SNPM04241 | LG15 | 23.7 |
| SNPM02923 | LG15 | 23.7 |
| SNPM00784 | LG15 | 23.7 |
| SNPM02056 | LG15 | 24.8 |
| SNPM04289 | LG15 | 25.9 |
| SNPM00062 | LG15 | 27.6 |
| SNPM01853 | LG15 | 29.9 |
| SNPM04033 | LG15 | 29.9 |
| SNPM03461 | LG15 | 29.9 |
| SNPM02569 | LG15 | 30.8 |
| SNPM03997 | LG15 | 31.7 |
| SNPM01951 | LG15 | 31.7 |
| SNPM02965 | LG15 | 31.7 |
| SNPM03396 | LG15 | 32.6 |
| SNPM00837 | LG15 | 32.6 |
| SNPM04976 | LG15 | 32.6 |
| SNPM02649 | LG15 | 36.0 |
| SNPM01477 | LG15 | 36.6 |
| SNPM03447 | LG15 | 36.6 |
| SNPM01840 | LG15 | 37.9 |
| SNPM02341 | LG15 | 38.6 |
| SNPM04932 | LG15 | 39.2 |
| SNPM03009 | LG15 | 40.1 |
| SNPM03800 | LG15 | 41.0 |
| SNPM04218 | LG15 | 41.0 |
| SNPM03211 | LG15 | 42.8 |
| SNPM04016 | LG15 | 43.7 |
| SNPM01070 | LG15 | 44.6 |
| SNPM04708 | LG15 | 46.4 |
| SNPM01445 | LG15 | 46.4 |
| SNPM00956 | LG15 | 46.6 |
| SNPM02906 | LG15 | 47.4 |
| SNPM02893 | LG15 | 49.2 |
| SNPM01971 | LG15 | 49.2 |
| SNPM03243 | LG15 | 51.7 |
| SNPM04101 | LG15 | 54.1 |
| SNPM00406 | LG15 | 55.4 |
| SNPM04646 | LG15 | 55.4 |
| SNPM04692 | LG15 | 55.8 |
| SNPM03849 | LG15 | 55.8 |
| SNPM03607 | LG15 | 58.2 |
| SNPM03966 | LG15 | 59.4 |
| SNPM04855 | LG15 | 59.4 |
| sEg00009 | LG15 | 61.8 |
| SNPM03095 | LG15 | 62.0 |
| sMo00208b | LG15 | 69.5 |
| SNPM01190 | LG15 | 70.3 |
| SNPM00927 | LG15 | 70.3 |
| SNPM02853 | LG15 | 70.3 |
| SNPM04801 | LG15 | 71.0 |
| SNPM03261 | LG15 | 71.0 |
| SNPM00285 | LG15 | 71.0 |
| SNPM04642 | LG15 | 72.6 |
| SNPM04263 | LG15 | 75.8 |
| SNPM04670 | LG15 | 75.8 |
| SNPM05017 | LG15 | 77.1 |
| SNPM00895 | LG15 | 79.6 |
| SNPM00146 | LG15 | 80.2 |
| SNPM00273 | LG15 | 80.3 |
| SNPM00306 | LG15 | 80.3 |
| SNPM00984 | LG15 | 86.3 |
| SNPM00189 | LG15 | 86.3 |
| SNPM00058 | LG15 | 89.9 |
| SNPM00556 | LG15 | 89.9 |
| SNPM03045 | LG15 | 89.9 |
| SNPM02769 | LG15 | 89.9 |
| SNPM02502 | LG15 | 89.9 |
| SNPM00776 | LG15 | 94.0 |
| SNPM03720 | LG15 | 94.0 |
| SNPM04058 | LG15 | 96.5 |
| SNPM02527 | LG15 | 97.3 |
| SNPM03285 | LG15 | 98.1 |
| SNPM02704 | LG16 | 0.0 |
| SNPM02009 | LG16 | 0.0 |
| SNPM01866 | LG16 | 9.4 |
| SNPM03117 | LG16 | 10.5 |
| SNPM00304 | LG16 | 11.5 |
| SNPM01088 | LG16 | 12.1 |
| Hght7_oSSR | LG16 | 14.3 |
| SNPM02925 | LG16 | 14.3 |
| SNPM01946 | LG16 | 16.6 |
| SNPM00574 | LG16 | 18.8 |
| SNPM03531 | LG16 | 18.8 |
| SNPM00317 | LG16 | 18.8 |
| SNPM04558 | LG16 | 19.3 |
| SNPM04933 | LG16 | 23.9 |
| SNPM01288 | LG16 | 28.6 |
| SNPM00649 | LG16 | 29.6 |
| SNPM01943 | LG16 | 31.0 |
| SNPM01816 | LG16 | 35.1 |
| SNPM00804 | LG16 | 35.1 |
| SNPM00636 | LG16 | 38.8 |
| SNPM00176 | LG16 | 38.8 |
| SNPM04759 | LG16 | 38.8 |
| SNPM01254 | LG16 | 38.8 |
| SNPM01978 | LG16 | 39.4 |
| SNPM03520 | LG16 | 39.6 |
| SNPM00586 | LG16 | 43.5 |
| SNPM01057 | LG16 | 44.2 |
| SNPM00907 | LG16 | 45.7 |
| sMo00109 | LG16 | 47.8 |
| sMg00058 | LG16 | 47.8 |
| SNPM00394 | LG16 | 48.3 |
| SNPM05007 | LG16 | 48.3 |
| SNPM02036 | LG16 | 48.3 |
| SNPM01404 | LG16 | 50.5 |
| SNPM03842 | LG16 | 50.5 |
| KA2_SSRB | LG16 | 52.3 |
| SNPM04001 | LG16 | 52.3 |
| SNPM01657 | LG16 | 52.9 |
| SNPM02607 | LG16 | 54.8 |
| SNPM00298 | LG16 | 55.4 |
| SNPM02115 | LG16 | 55.4 |
| SNPM03459 | LG16 | 57.9 |
| sMg00136 | LG16 | 58.3 |
