# Supplementary material for: An integrated linkage map of interspecific backcross 2 (BC2) populations reveals QTLs associated with fatty acid composition and vegetative parameters influencing compactness in oil palm
Source: BMC Plant Biol. 2020 Jul 29;20:356. doi: 10.1186/s12870-020-02563-5 (PMC7391521; doi:10.1186/s12870-020-02563-5)
Supplement: Supplementary file 5 — Additional file 5. Map integration (consensus map) of populations 2.6–1 and 2.6–5 for genetic linkage groups 1–16. [file 12870_2020_2563_MOESM5_ESM.docx]

Additional file 5: Map integration (consensus map) of populations 2.6-1 and 2.6-5 for genetic linkage groups 1 - 16

**Linkage Group 1**

LG1_BC2 2.6-1

LG1_Integrated

LG1a_BC2 2.6-5

LG1b_BC2 2.6-5

**Linkage Group 2**

LG2_Integrated

LG2_BC2 2.6-1

LG2_BC2 2.6-5

**Linkage Group 3**

LG3_BC2 2.6-1

LG3_Integrated

LG3_BC2 2.6-5

**Linkage Group 4**

LG4_BC2 2.6-1

LG4_Integrated

LG4_BC2 2.6-5

**Linkage Group 5**

LG5_BC2 2.6-1

LG5_Integrated

LG5_BC2 2.6-5

**Linkage Group 6**

LG6_BC2 2.6-1

LG6_Integrated

LG6_BC2 2.6-5

**Linkage Group 7**

LG7_BC2 2.6-1

LG7_Integrated

LG7_BC2 2.6-5

**Linkage Group 8**

LG8_BC2 2.6-1

LG8_Integrated

LG8_BC2 2.6-5

**Linkage Group 9**

LG9_Integrated

LG9_BC2 2.6-5

LG9_BC2 2.6-1

**Linkage Group 10**

LG10_Integrated

LG10_BC2 2.6-1

LG10_BC2 2.6-5

**Linkage Group 11**

LG11_Integrated

LG11_BC2 2.6-1

LG11_BC2 2.6-5

**Linkage Group 12**

LG12_Integrated

LG12_BC2 2.6-1

LG12_BC2 2.6-5

**Linkage Group 13**

LG13_Integrated

LG13_BC2 2.6-1

LG13_BC2 2.6-5

**Linkage Group 14**

LG14_Integrated

LG14_BC2 2.6-1

LG14_BC2 2.6-5

**Linkage Group 15**

LG15_BC2 2.6-1

LG15_Integrated

LG15b_BC2 2.6-5

LG15a_BC2 2.6-5

**Linkage Group 16**

LG16_Integrated

LG16_BC2 2.6-1

LG16_BC2 2.6-5
