# Supplementary material for: An integrated linkage map of interspecific backcross 2 (BC2) populations reveals QTLs associated with fatty acid composition and vegetative parameters influencing compactness in oil palm
Source: BMC Plant Biol. 2020 Jul 29;20:356. doi: 10.1186/s12870-020-02563-5 (PMC7391521; doi:10.1186/s12870-020-02563-5)
Supplement: Supplementary file 6 — Additional file 6. List of QTL associated with the compactness traits and FAC in the independent and the integrated maps determined via Genstat. For a particular trait (Ex. RL and C16:0), a number of markers are present in the QTL interval. Although almost the same genomic region (determined via position on LG) is linked to the QTL in independent populations, a different marker is at times located closest to the QTL peak, in the integrated map. The original marker identified in the independent populations remains significant. [file 12870_2020_2563_MOESM6_ESM.docx]

| QTL | 2.6-1 | | | | 2.6-5 | | | | Integrated | | | |
| --- | --- | --- | --- | --- | --- | --- | --- | --- | --- | --- | --- | --- |
| HI | Marker | LG | Position | LOD | Marker | LG | Position | LOD | Marker | LG | Position | LOD |
|  | SNPM00563  SNPM04311 | 4  5 | 4.3  99.7 | 3.17  3.46 | SNPM00249  SNPM00010 | 4  7 | 3.4  111.9 | 5.99  4.57 | SNPM00563  SNPM00010 | 4  7 | 4.3  111.9 | 7.77  3.76 |
| RL | SNPM02501  SNPM04576 | 4  8 | 0.9  81.6 | 3.04  4.23 | SNPM01565 | 13 | 37.7 | 3.41 | SNPM03201  SNPM03772  SNPM03676 | 4  8  11 | 11.3  92.6  38.7 | 3.79  3.17  4.17 |
| PCS | SNPM00563  sEg00213 | 4  8 | 4.3  39.1 | 4.46  3.89 | SNPM00151  SNPM02535 | 4  4 | 4.3  193.1 | 4.51  3.52 | SNPM00563  SNPM02535  sEg00213 | 4  4  8 | 4.3  193.1  139.9 | 7.70  3.20  3.81 |
| C16:0 | SNPM00796 | 1 | 133.4 | 4.24 | sPSc00306 | 1 | 112.8 | 3.10 | SNPM00796 | 1 | 133.4 | 3.59 |
| C18:1 | SNPM02507  SNPM03249 | 4  8 | 169.2  44.0 | 3.94  3.18 | SNPM00274 | 12 | 31.4 | 6.24 | SNPM02507  SNPM03249  SNPM00274 | 4  8  12 | 169.2  44.0  31.4 | 3.44  3.19  5.97 |
| C18:2 | SNPM00249  SNPM04311  SNPM01190 | 4  5  15 | 3.4  99.7  70.3 | 3.64  3.39  3.59 | SNPM00249 | 4 | 3.4 | 5.84 | SNPM01602  SNPM00249  SNPM01190 | 1  4  15 | 124.9  3.4  70.3 | 4.62  8.19  5.14 |
| IV | SNPM01452  SNPM03285 | 1  15 | 132.5  98.1 | 11.60  5.47 | SNPM02063  SNPM04197 | 1  3 | 102.4  15.8 | 7.87  3.09 | SNPM01452  SNPM03285 | 1  15 | 132.5  98.1 | 13.02  5.98 |
| Carotene |  |  |  |  | SNPM02349  SNPM00729  SNPM03960  SNPM03145 | 3  4  7  10 | 4.4  181.8  108.5  4.33 | 4.11  4.27  75.7  4.87 | SNPM02349  SNPM03960  SNPM03921 | 3  7  10 | 4.4  108.5  77.4 | 5.12  3.73  3.63 |

Additional file 6: List of QTL associated with the compactness traits and FAC in the independent and the integrated maps determined via Genstat. For a particular trait (Ex. RL and C16:0), a number of markers are present in the QTL interval. Although almost the same genomic region (determined via position on LG) is linked to the QTL in independent populations, a different marker is at times located closest to the QTL peak, in the integrated map. The original marker identified in the independent populations remains significant.

Note: HI = height increment, RL= rachis length, PCS = petiole cross section, C16:0 = palmitic acid content, C18:1 = oleic acid content, C18:2 = linoleic acid content, IV = iodine value, Carotene = Carotene content
