# Supplementary material for: An integrated linkage map of interspecific backcross 2 (BC2) populations reveals QTLs associated with fatty acid composition and vegetative parameters influencing compactness in oil palm
Source: BMC Plant Biol. 2020 Jul 29;20:356. doi: 10.1186/s12870-020-02563-5 (PMC7391521; doi:10.1186/s12870-020-02563-5)
Supplement: Supplementary file 7 — Additional file 7. Significant major QTLs detected for respective traits using MapQTL. Horizontal line indicates the 95% genome wide significant threshold value for declaring a QTL. [file 12870_2020_2563_MOESM7_ESM.doc]

Additional file 7: Significant major QTLs detected for respective traits using MapQTL. Horizontal line indicates the 95% genome wide significant threshold value for declaring a QTL.

| **QTL** | **QTL Region** |
| --- | --- |
| **Height Increment** | 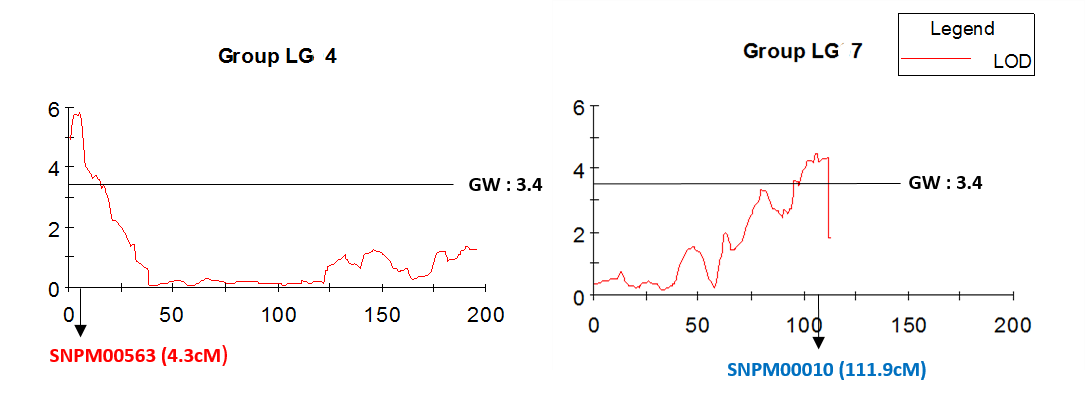 |
| **Rachis**  **Length** | 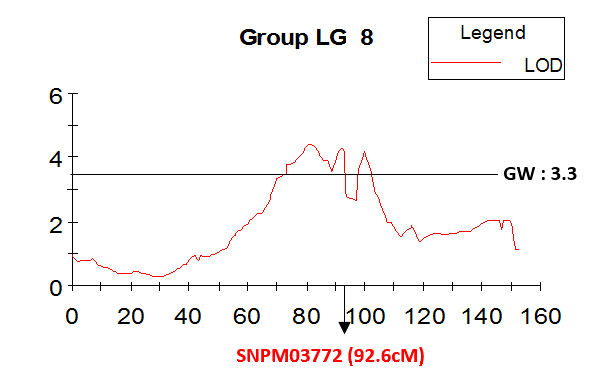 |
| **Petiole Cross Section** | 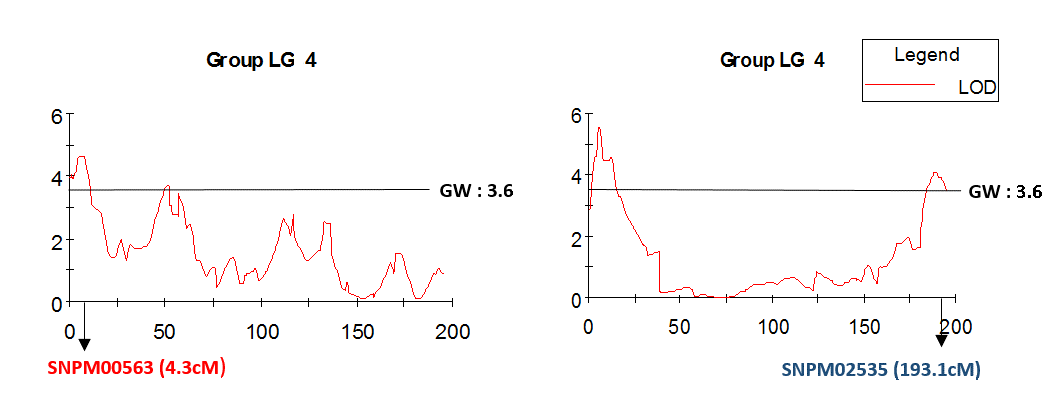  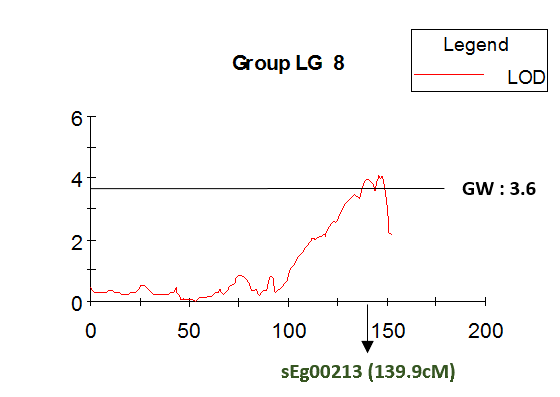 |
| **QTL** | **QTL Region** |
| **C16:0**  **Content** | 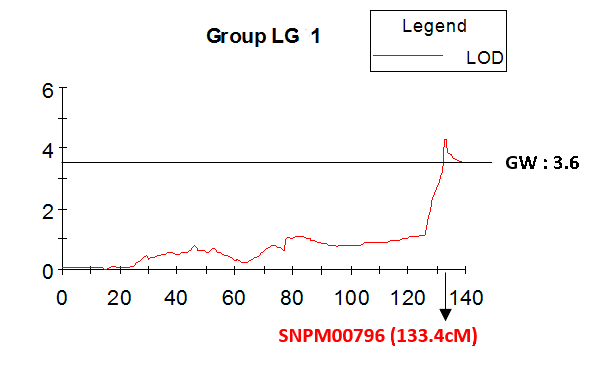 |
| **C18:1**  **Content** | 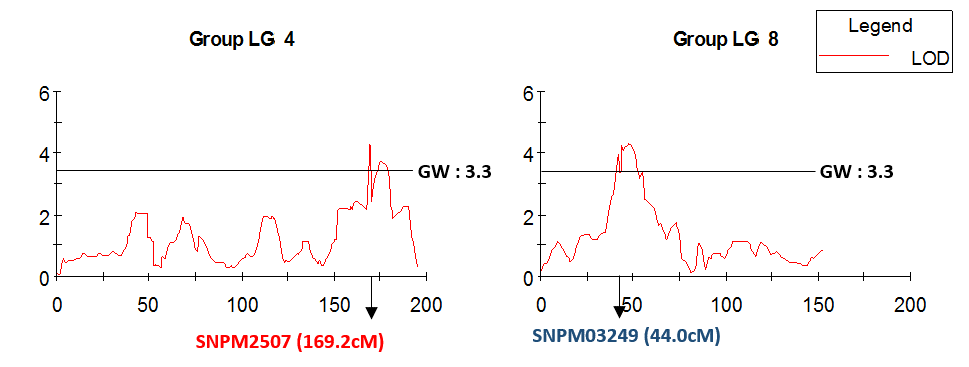 |
| **C18:2**  **Content** | 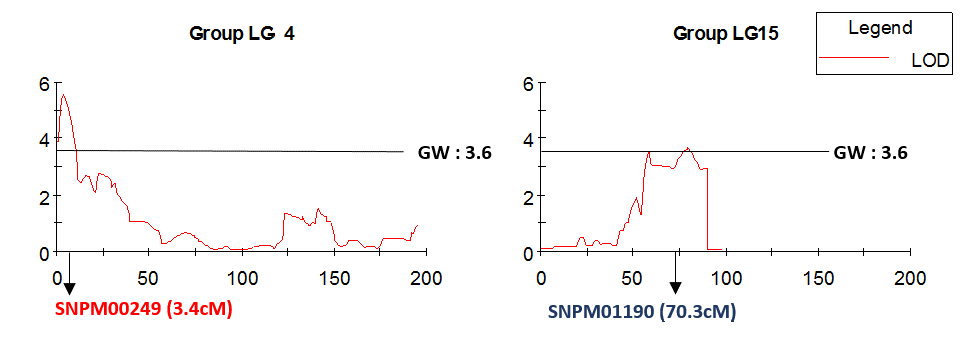 |

| **QTL** | **QTL Region** |
| --- | --- |
| **Iodine Value** | 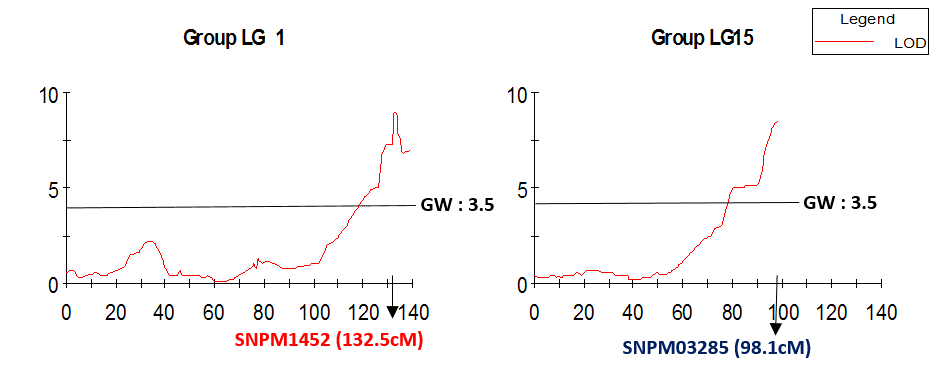 |
| **Carotene**  **Content** | 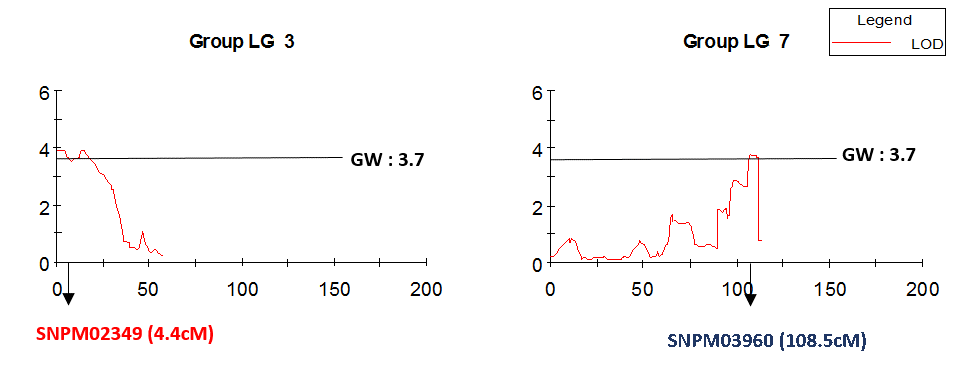  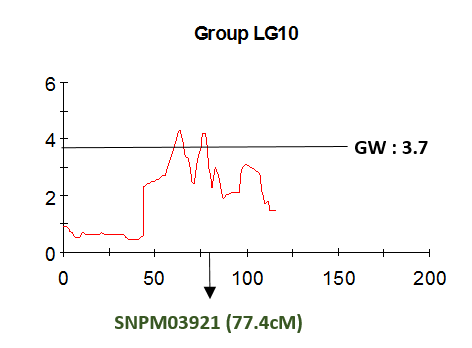 |
