# Supplementary material for: An integrated linkage map of interspecific backcross 2 (BC2) populations reveals QTLs associated with fatty acid composition and vegetative parameters influencing compactness in oil palm
Source: BMC Plant Biol. 2020 Jul 29;20:356. doi: 10.1186/s12870-020-02563-5 (PMC7391521; doi:10.1186/s12870-020-02563-5)
Supplement: Supplementary file 8 — Additional file 8. Blast results to the genome build identified 21 candidate genes within the QTL confidence intervals affecting the vegetative traits and FAC. [file 12870_2020_2563_MOESM8_ESM.docx]

Additional file 8: Blast results to the genome build identified 21 candidate genes within the QTL confidence intervals affecting the vegetative traits and FAC.

|  | **Gene: Accession Number** | **Putative Gene Function** | **QTL** | **QTL Interval (Physical length)** |
| --- | --- | --- | --- | --- |
| **1** | PX3: XM010938952 | Play a role in vascular tissues development [1]. | RL (LG8)  PCS(LG8) | 34,129,696 – 42,192,195  56,219,329 – 63,280,735 |
| **2** | DME: XM010942215 | Required for stable reproducible patterns of floral and vegetative development. [2,3,4]. | RL (LG8)  HI (LG4)  RL (LG11)  PCS(LG8) | 34,129,696 – 42,192,195  17,286 – 6,689,582  39,644,261 – 46,104,351  56,219,329 – 63,280,735 |
| **3** | SEC15B: XM010916171 | Involved in polarized cell growth and organ morphogenesis [5]. | PCS (LG4) | 59,554,972 – 63,965,353 |
| **4** | FERONIA: XM010922882 | Required for cell elongation during vegetative growth [6]. | PCS (LG4) | 59,554,972 – 63,965,353 |
| **5** | THESEUS1: XM010907290  XM010906936 | Receptor-like protein kinase required for cell elongation during vegetative growth [7]. | PCS (LG4) | 59,554,972 – 63,965,353 |
| **6** | CRRSP55: XM010909959 | Response to Karrikins, a group of plant growth regulators [8]. | PCS (LG4) | 59,554,972 – 63,965,353 |
| **7** | ERECTA: XM010910431 | Probable major trait regulating canalization (maintenance of phenotype despite varying environment) in many aspects of the plant physiology (e.g. plant morphology, light-dependent leaves number, branch number, flowering time, phytate and mineral concentrations) [9,10]. | PCS (LG4) | 59,554,972 – 63,965,353 |
| **8** | D6PK: XM019847203  XM010910470 | Protein kinase that regulates the auxin transport activity of PIN auxin efflux facilitators by direct phosphorylation [11]. | PCS (LG4) | 59,554,972 – 63,965,353 |
| **9** | PSY1R: XM010919022 | Regulates a signaling cascade involved in cellular proliferation and plant growth [12]. | HI (LG7) | 48,171 – 17,503,030 |
| **10** | EXO70A1: XM010919145 | Involved in polarized cell growth and organ morphogenesis [13]. | HI (LG7) | 48,171 – 17,503,030 |
| **11** | SYD: XM008814253 | Repressor of the meristem identity switch from vegetative to reproductive development probably by modulating chromatin state [14]. | HI (LG7) | 48,171 – 17,503,030 |
| **12** | TPS6: XM019849452  XR831474  XM010919209 | Regulates plant architecture, shape of epidermal pavement cells and branching of trichomes [15]. | HI (LG7) | 48,171 – 17,503,030 |
| **13** | CR4: XM019852706 | Controls formative cell division in meristems [16]. | HI (LG7)  RL (LG11) | 48,171 – 17,503,030  39,644,261 – 46,104,351 |
| **14** | BIG: XM010943964 | Required for auxin efflux and polar auxin transport (PAT) influencing auxin-mediated developmental responses (e.g. cell elongation, apical dominance, lateral root production, inflorescence architecture, general growth and development) [17,18,19]. | HI (LG7)  PCS(LG8) | 48,171 – 17,503,030  56,219,329 – 63,280,735 |
| **15** | RLP12: XM019846004 | May be involved in brassinosteroid-mediated plant growth and development via auxin regulation [20]. | HI (LG7) | 48,171 – 17,503,030 |
| **16** | HERK1: XM010920802 | Receptor-like protein kinase required for cell elongation during vegetative growth [7]. | RL (LG11) | 39,644,261 – 46,104,351 |
| **17** | SBT1: XM010920893 | Serine protease that cleaves the phytosulfokines, plant growth factors or peptide hormones that promotes plant cell differentiation, organogenesis and somatic embryogenesis as well as cell proliferation [21]. | RL (LG11) | 39,644,261 – 46,104,351 |
| **18** | SCL22: XM010921231  XM010921232  XR002164612  XM010921230 | Probable transcription factor involved in plant development [22]. | RL (LG11) | 39,644,261 – 46,104,351 |
| **19** | BAM1: XM010914345 | Required for the development of high-ordered vascular strands within the leaf and a correlated control of leaf shape, size and symmetry [23]. | RL (LG11) | 39,644,261 – 46,104,351 |
| **20** | KCS11: XM010916640 | Active on both saturated and mono-unsaturated acyl chains C16 to C20 [24]. | IV (LG1)  C18:2 (LG1) | 367,701 – 2,032,527  367,701 – 2,032,527 |
| **21** | CUT1: XM010917870 | Required for elongation of C24 fatty acids, an essential step of the cuticular wax production Major condensing enzyme for stem wax and pollen coat lipid biosynthesis [25]. | IV (LG1)  C18:2 (LG1) | 367,701 – 2,032,527  367,701 – 2,032,527 |

Note: RL= rachis length, HI = height increment, PCS = petiole cross section, IV = iodine value, C18:2 = linoleic acid content

Reference:

1. Wang J, Kucukoglu M, Zhang L, Chen P, Decker D, Nilsson O, Jones B, Sandberg G, Zheng B. The Arabidopsis LRR-RLK, PXC1, is a regulator of secondary wall formation correlated with the TDIF-PXY/TDR-WOX4 signaling pathway. BMC Plant Biology. 2013;13: 94.

2. Choi Y, Gehring M, Johnson L, Hannon M, Harada JJ, Goldberg RB, Jacobsen SE, Fischer RL. DEMETER, a DNA glycosylase domain protein, is required for endosperm gene imprinting and seed viability in Arabidopsis.Cell. 2002;110: 33-42.

3. Choi Y, Harada JJ, Goldberg RB, Fischer RL. An invariant aspartic acid in the DNA glycosylase domain of DEMETER is necessary for transcriptional activation of the imprinted MEDEA gene. Proc. Natl. Acad. Sci. U.S.A. 2004; 101:7481-7486.

4. Morales-Ruiz T, Ortega-Galisteo AP, Ponferrada-Marin MI, Martinez-Macias MI, Ariza RR, Roldan-Arjona T. DEMETER and REPRESSOR OF SILENCING 1 encode 5-methylcytosine DNA glycosylases. Proc. Natl. Acad. Sci. U.S.A. 2006; 103:6853-6858.

5. Fendrych M, Synek L, Pecenkova T, Toupalova H, Cole R, Drdova E, Nebesarova J, Sedinova M, Hala M, Fowler JE, Zarsky V. The Arabidopsis exocyst complex is involved in cytokinesis and cell plate maturation.Plant Cell, 2010; 22:3053-3065.

6. Haruta M, Sabat G, Stecker K, Minkoff BB, Sussman MR. A peptide hormone and its receptor protein kinase regulate plant cell expansion. Science. 2014; 343:408-411.

7. Guo H, Li L, Ye H, Yu X, Algreen A, Yin Y. Three related receptor-like kinases are required for optimal cell elongation in Arabidopsis thaliana. Proc. Natl. Acad. Sci. U.S.A. 2009; 106: 7648-7653.

8. Nelson DC, Flematti GR, Riseborough JA, Ghisalberti EL, Dixon KW, Smith SM. Karrikins enhance light responses during germination and seedling development in Arabidopsis thaliana. Proc. Natl. Acad. Sci. U.S.A. 2010; 107:7095-7100.

9. Hall MC, Dworkin I, Ungerer MC, Purugganan M. Genetics of microenvironmental canalization in Arabidopsis thaliana. Proc. Natl. Acad. Sci. U.S.A. 2007; 104:13717-13722.

10. Uchida N, Tasaka M. Regulation of plant vascular stem cells by endodermis-derived EPFL-family peptide hormones and phloem-expressed ERECTA-family receptor kinases. J. Exp. Bot. 2013; 64:5335-5343.

11. Willige BC, Ahlers S, Zourelidou M, Barbosa IC, Demarsy E, Trevisan M, Davis PA, Roelfsema MR, Hangarter R, Fankhauser C, Schwechheimer C. D6PK AGCVIII kinases are required for auxin transport and phototropic hypocotyl bending in Arabidopsis. Plant Cell. 2013; 25:1674-1688.

12. Amano Y, Tsubouchi H, Shinohara H, Ogawa M, Matsubayashi Y. Tyrosine-sulfated glycopeptide involved in cellular proliferation and expansion in Arabidopsis. Proc. Natl. Acad. Sci. U.S.A. 2007; 104:18333-18338.

13. Synek L, Schlager N, Elias M, Quentin M, Hauser MT, Zarsky V. At EXO70A1, a member of a family of putative exocyst subunits specifically expanded in land plants, is important for polar growth and plant development. Plant J. 2006; 48:54-72.

14. Wagner D, Meyerowitz EM. SPLAYED, a novel SWI/SNF ATPase homolog, controls reproductive development in Arabidopsis. Curr. Biol. 2002; 12:85-94.

15. Chary SN, Hicks GR, Choi YG, Carter D, Raikhel NV. Trehalose-6-phosphate synthase/ phosphatase regulates cell shape and plant architecture in Arabidopsis. Plant Physiol. 2008; 146:97-107.

16. Pu CX, Ma Y, Wang J, Zhang YC, Jiao XW, Hu YH, Wang LL, Zhu ZG, Sun D, Sun Y. Crinkly4 receptor-like kinase is required to maintain the interlocking of the palea and lemma, and fertility in rice, by promoting epidermal cell differentiation. Plant J. 2012; 70:940-953.

17. Li HM, Altschmied L, Chory J. Arabidopsis mutants define downstream branches in the phototransduction pathway. Genes Dev. 1994; 8:339-349.

18. Sponsel VM, Schmidt FW, Porter SG, Nakayama M, Kohlstruk S, Estelle M. Characterization of new gibberellin-responsive semidwarf mutants of arabidopsis. Plant Physiol. 1997; 115:1009-1020.

19. Lopez-Bucio J, Hernandez-Abreu E, Sanchez-Calderon L, Perez-Torres A, Rampey RA, Bartel B, Herrera-Estrella L. An auxin transport independent pathway is involved in phosphate stress-induced root architectural alterations in Arabidopsis. Identification of BIG as a mediator of auxin in pericycle cell activation. Plant Physiol. 2005; 137:681-691.

20. Kim MH, Kim Y, Kim JW, Lee HS, Lee WS, Kim SK, Wang ZY, Kim SH. Identification of Arabidopsis BAK1-associating receptor-like kinase 1 (BARK1) and characterization of its gene expression and brassinosteroid-regulated root phenotypes. Plant Cell Physiol. 2013; 54(10):1620-34.

21. Srivastava R, Liu JX, Howell SH. Proteolytic processing of a precursor protein for a growth-promoting peptide by a subtilisin serine protease in Arabidopsis.Plant J. 2008; 56:219-227.

22. Riechmann JL, Heard J, Martin G, Reuber L, Jiang C, Keddie J, Adam L, Pineda O, Ratcliffe OJ, Samaha RR, Creelman R, Pilgrim M, Broun P, Zhang JZ, Ghandehari D, Sherman BK, Yu G. Arabidopsis transcription factors: genome-wide comparative analysis among eukaryotes. Science. 2000; 290:2105-2110.

23. DeYoung BJ, Bickle KL, Schrage KJ, Muskett P, Patel K, Clark SE. The CLAVATA1-related BAM1, BAM2 and BAM3 receptor kinase-like proteins are required for meristem function in Arabidopsis. Plant J. 2006; 45:1-16.

24. Blacklock BJ, Jaworski JG. Substrate specificity of Arabidopsis 3-ketoacyl-CoA synthases. Biochem. Biophys. Res. Commun. 2006; 346:583-590.

25. Millar AA, Clemens S, Zachgo S, Giblin EM, Taylor DC, Kunst L. CUT1, an Arabidopsis gene required for cuticular wax biosynthesis and pollen fertility, encodes a very-long-chain fatty acid condensing enzyme. Plant Cell. 1999; 11:825-838.
